# Titanium(III)‐Catalyzed Reductive Decyanation of Geminal Dinitriles by a Non‐Free‐Radical Mechanism

**DOI:** 10.1002/anie.201908372

**Published:** 2019-10-23

**Authors:** Jens Weweler, Sara L. Younas, Jan Streuff

**Affiliations:** ^1^ Institut für Organische Chemie Albert-Ludwigs-Universität Freiburg Albertstr. 21 79104 Freiburg im Breisgau Germany

**Keywords:** catalysis, C−C activation, cyanides, electron transfer, titanium

## Abstract

A titanium‐catalyzed mono‐decyanation of geminal dinitriles is reported. The reaction proceeds under mild conditions, tolerates numerous functional groups, and can be applied to quaternary malononitriles. A corresponding desulfonylation is demonstrated as well. Mechanistic experiments support a catalyst‐controlled cleavage without the formation of free radicals, which is in sharp contrast to traditional stoichiometric radical decyanations. The involvement of two Ti^III^ species in the C−C cleavage is proposed, and the beneficial role of added ZnCl_2_ and 2,4,6‐collidine hydrochloride is investigated.

## Graphical abstract

**A titanium(III) complex** catalyzes the reductive C−CN cleavage of geminal dinitriles. The reaction proceeds under unusually mild reaction conditions and tolerates numerous common functional groups. Mechanistic studies support a catalyst‐controlled C−CN scission instead of a free‐radical pathway.

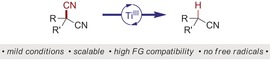

The controlled cleavage of carbon–carbon bonds is a highly topical research area and a challenge to modern transition‐metal catalysis.^1^, ^2^, ^3^ One particular type is the cleavage of C−CN bonds, which can be used either as an entry point for subsequent bond‐constructing events,^4^ or for a reductive, selective decyanation.^5^, ^6^ In this context, the reductive decyanation of geminal dinitriles provides direct access to functionalized alkylnitriles from easy‐to‐prepare malononitrile precursors, making it a powerful alternative to conventional nitrile α‐functionalizations.^7^ However, only a limited number of stoichiometric examples have been reported for this transformation to date. These comprise traditional free‐radical defunctionalizations with tin hydride, tris(trimethylsilyl)silane, and NHC–borane reagents as hydrogen radical donors,^8^ or strongly reducing conditions with stoichiometric amounts of SmI_2_/HMPA and other so‐called “super‐electron donors” (Scheme 1).^9^, ^10^ With the goal to close this methodological gap, we herein report a broadly applicable catalytic reductive decyanation that proceeds in the presence of a titanium(III) single‐electron‐transfer catalyst.^11^, ^12^ The reaction is not to be confused with free‐radical nitrile translocation reactions.^13^

**Scheme 1:**
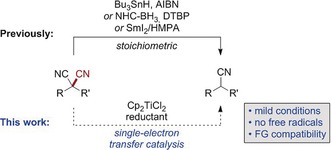
Free‐radical decyanations and the envisioned titanium(III) catalysis.

The catalytic decyanation was first investigated using 2‐benzylmalononitrile (**1 a**) as substrate, having both nitriles in a homobenzylic position (Scheme 2). An initial optimization study showed that nitrile **2 a** could be obtained in a good yield of 80 % after 48 h from a reaction with titanocene dichloride (10 mol %), zinc as reducing agent, and 2,4,6‐collidine hydrochloride (Coll⋅HCl) and chlorotrimethylsilane (TMSCl) as additives in THF at 35 °C. Without either additive, the yield of **2 a** was inferior. The reaction was highly chemoselective (spot‐to‐spot) and worked also for 2‐phenylmalononitrile (**1 b**), albeit with a significantly lower yield (30 %) of benzyl cyanide (**2 b**). Interestingly, it was found that adding **2 b** to the decyanation of **1 a** also greatly diminished the reaction outcome to 23 % yield. Based on previous reports on titanium(III)–nitrile complexes and our experience in titanium(III) catalysis involving nitriles,^14^, ^15^ product inhibition of the catalyst was concluded. Further experimentation revealed that this inhibition could be prevented and the catalyst activity even be improved by adding zinc chloride to the decyanations, giving 82 % yield for **2 a** and 74 % yield for **2 b** after only 24 h. This scenario was supported by preliminary DFT calculations, which confirmed the product inhibition and the liberation of the inhibited catalyst upon addition of ZnCl_2_.^16^ Only traces of the decyanation product were observed without the titanium catalyst.

**Scheme 2:**
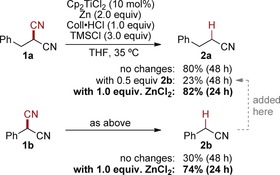
Initial optimization studies.

Several malononitriles were then decyanated accordingly on 0.5 mmol scale with a simple filtration as a sufficient workup procedure (Scheme 3). The reaction showed an unusually broad substrate scope for a C−CN cleavage method. For example, decyanation at a homobenzylic position proceeded smoothly in the presence of bromo (**2 c**), ester (**2 d**), acetoxy (**2 e**), nitrile (**2 f**), thioether (**2 g**), ether and free alcohol functions (**2 h**). The arylated malononitriles **1 i** and **1 j**, containing trifluoromethyl and methoxy groups, also underwent the decyanation to the corresponding benzyl nitriles **2 i** and **2 j** in 44 % and 70 % yield, respectively. Likewise, an *ortho*‐tolylmalononitrile was mono‐decyanated in 71 % yield (**2 k**). Symmetric and unsymmetric quaternary malononitriles could be employed as well, which led to nitriles **2 l**–**2 n** (54–88 %). Here, the increased steric hindrance led to a slower reaction, which was compensated by a prolonged reaction time of 48 h. Other structurally diverse substrates containing cyclohexyl and indole groups smoothly underwent the decyanation to the corresponding nitriles **2 o** and **2 p**. Malononitriles containing a styryl moiety or a β‐vinyl group were decyanated to give **2 q** and **2 r** in 72 % and 42 % (48 h) yield, respectively. The catalytic reductive decyanation reaction of **1 a** was also demonstrated on a 9 mmol (1.4 g) scale, resulting in a slightly lower yield (70 %).

**Scheme 3:**
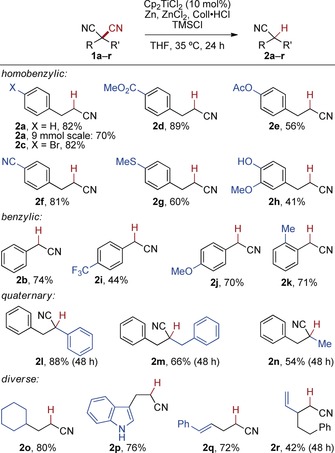
Scope of the catalytic C−CN cleavage reaction.

We also tested whether the reaction could be extended towards the removal of a different functional group, and it was found that α‐cyanosulfone **3** indeed underwent clean desulfonylation to **2 a** in 56 % yield (Scheme 4). As thiophenol was observed as a byproduct, the amounts of zinc and hydrochloride were increased to compensate for the additional sulfone reduction and thiolate protonation. Unreacted **3** accounted for the mass balance, and no background reaction took place. The further elaboration of this catalytic desulfonylation will be reported separately.

**Scheme 4:**
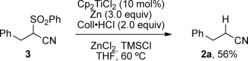
Titanium(III)‐catalyzed desulfonylation.

A series of experiments were then carried out to elucidate the decyanation mechanism. Geminal dinitriles with a tethered pent‐4‐en‐1‐yl group were previously reported to readily undergo 5‐*exo*‐trig cyclization after a homolytic C−CN cleavage under free‐radical conditions.8b, 8c The titanium(III) catalysis, however, led to exclusive decyanation of compound **4** to nitrile **5** without the generation of the cyclization product **6** (Scheme 5 a). Further proof of a non‐free‐radical mechanism was unambiguously obtained by the decyanation of radical clock substrate **7**, which only led to the desired nitrile **8** without the formation of any ring‐opened products. The decyanation of **1 a** was then carried out with 94 %‐deuterated Coll⋅DCl to confirm that the newly introduced hydrogen atom was transferred by proton transfer from the collidinium salt. A deuterium incorporation of 68 % was achieved, while a reaction with Coll⋅HCl run in [D_8_]THF as the solvent resulted in no deuterium transfer. Next, the order in catalyst was determined from two experiments run with 5.0 and 12.5 mol % of Cp_2_TiCl_2_ on a 4 mmol scale that were followed by NMR analysis of taken samples (Figure 1). Visual kinetic analysis then revealed the order in catalyst by time normalization:^17^ A plot of the yield versus *t*×[cat]^2.0^ led to an excellent overlay of the two curves, which confirmed the second order in the catalyst.

**Figure 1:**
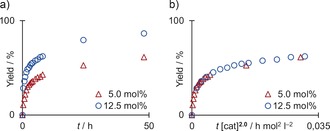
a) Reaction kinetics of experiments with 5.0 and 12.5 mol % catalyst. b) Variable time normalization analysis revealing a catalyst order of 2.0.

**Scheme 5:**
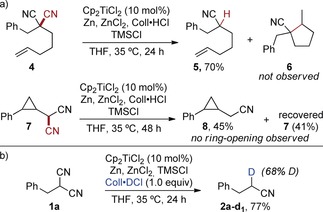
a) Negative radical clock experiments. b) Deuteration experiment confirming the introduction of the new hydrogen by proton transfer.

We also investigated the direct effect of the added ZnCl_2_ on the titanium(III) catalyst by cyclic voltammetry (Figure 2). The voltammograms of Zn‐reduced Cp_2_TiCl_2_ were recorded in the presence of Coll⋅HCl, ZnCl_2_, or Coll⋅HCl and ZnCl_2_ (each in tenfold excess to simulate the conditions of the catalysis). With added Coll⋅HCl, the ion pair [CollH]^+^[Cp_2_TiCl_2_]^−^ (*E*=−1.25 V) and the dimer [(Cp_2_TiCl)_2_] (*E*=−0.81 V) were observed.^18^, ^19^, ^20^ Usually, Zn‐reduced solutions of Cp_2_TiCl_2_ also show the monomer [Cp_2_TiCl] (*E*=−0.75 V),^20^ which appeared to be absent under these conditions. With added ZnCl_2_, however, the cation [Cp_2_Ti]^+^ (*E*=−0.43 V)^20^ became the only observable species.^21^ This was in agreement with the earlier proposal that [Cp_2_TiCl] and ZnCl_2_ form a closely bound ion pair in solution.^22^, ^23^ Based on previous studies on cationic titanium(III) species, we concluded that this ion pair was [Cp_2_Ti]^+^[ZnCl_3_]^−^.12g, 15 When Coll⋅HCl and ZnCl_2_ were added simultaneously, the oxidation peak of [Cp_2_Ti]^+^ vanished again and [Cp_2_TiCl] appeared as the only visible species.^20^, ^21^ Hence, the combination of Coll⋅HCl and ZnCl_2_ led to a shift in the equilibria connecting all species towards the catalyst monomer. We attributed this to the formation of [CollH]^+^[ZnCl_3_(THF)]^−^ from Coll⋅HCl and ZnCl_2_(THF)_2_, which was calculated to release Δ*G*=−8.5 kcal mol^−1^.^16^ Whether the addition of ZnCl_2_ also has a beneficial effect on the catalyst reduction step, as recently found for a titanium(III) catalysis with Cp*TiCl_3_,12e was not investigated at this time.

**Figure 2:**
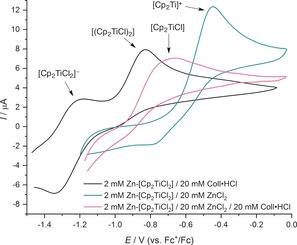
Cyclic voltammograms of Zn–Cp_2_TiCl_2_ with added Coll⋅HCl, added ZnCl_2_, and added ZnCl_2_ and Coll⋅HCl, all at a sweep rate of 0.1 V s^−1^.

A first mechanistic proposal was derived (Scheme 6), in which the geminal dinitrile coordinates two equivalents of in situ generated [Cp_2_TiCl] (**A**), giving complex **B**. Then, single electron transfer (SET) from both titanium(III) centers triggers the C−C cleavage, giving one equivalent of ketenimine–titanium(IV) complex **C** and N‐coordinated titanium(IV) cyanide complex **D** or, alternatively, its C‐coordinated isomer. The participation of two titanium(III) species in the C−CN scission is in agreement with the observed second order in catalyst and the non‐free‐radical behavior. Protonation of **C** by Coll⋅HCl then releases the nitrile product and Cp_2_TiCl_2_ (**E**). The reaction of **D** with TMSCl simultaneously liberates the second equivalent of **E** and formally one equivalent of TMSCN. The formation of cyanide [presumably TMSCN or Zn(CN)_2_] was confirmed by ion chromatographic analysis of the aqueous layer obtained from the workup. Finally, zinc regenerates the titanium(III) catalyst.

**Scheme 6:**
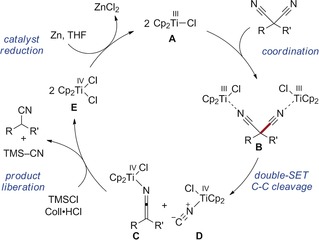
Proposed simplified catalytic cycle.

In conclusion, a titanium(III)‐catalyzed decyanation of geminal dinitriles has been developed that represents the first example of such a decyanation reaction proceeding by single‐electron‐transfer catalysis. The reaction occurs under mild conditions, features a broad substrate scope, and shows excellent chemoselectivity. It has been demonstrated that the cleavage does not proceed through a free‐radical mechanism but via a unique catalyst‐controlled C−CN scission involving two titanium species, which renders it complementary to previous decyanation protocols. Further applications towards other C−C and C−heteroatom bond cleavage reactions are currently being studied and will be reported in due course.

## Conflict of interest

The authors declare no conflict of interest.

## Supporting information

As a service to our authors and readers, this journal provides supporting information supplied by the authors. Such materials are peer reviewed and may be re‐organized for online delivery, but are not copy‐edited or typeset. Technical support issues arising from supporting information (other than missing files) should be addressed to the authors.

Supplementary
