# Supplementary material for: Titanium(III)‐Catalyzed Reductive Decyanation of Geminal Dinitriles by a Non‐Free‐Radical Mechanism
Source: Angew Chem Int Ed Engl. 2019 Oct 23;58(49):17700–3. doi: 10.1002/anie.201908372 (PMC6899653; doi:10.1002/anie.201908372)
Supplement: Supplementary file 1 — Supplementary [file ANIE-58-17700-s001.pdf]

Angewandte Chemie
Eine Zeitschrift der Gesellschaft Deutscher Chemiker

Supporting Information

Titanium(III)-Catalyzed Reductive Decyanation of Geminal Dinitriles by a Non-Free-Radical Mechanism

*Jens Weweler, Sara L. Younas, and Jan Streuff**

anie_201908372_sm_miscellaneous_information.pdf

Table of Contents

Materials and Methods **S2**
Synthesis of the Precursors 1a–q, 3, 4, and 7 .... **S3**
Titanium Catalyzed Reductive Decyanation Reactions **S9**
General Catalytic Decyanation Procedure S9
Data for the Products **2a–r** S10
Titanium Catalyzed Reductive Desulfonylation of 3 .... **S14**
Negative Radical Clock Experiments and Data for 5 and 8 .... **S14**
Deuteration Experiments **S15**
Cyclic Voltammetry **S16**
Determination of the Order in Catalyst by Visual Kinetic Analysis **S18**
Qualitative Verification of Cyanide Formation **S20**
Computational Details **S22**
Coordinates S24
References **S31**
NMR Spectra of New Compounds **S34**

Materials and Methods

All reactions were carried out in flame-dried Schlenk tubes under argon atmosphere (argon 5.0) and using absolute solvents unless noticed otherwise. Absolute THF and absolute toluene were dried over potassium under argon atmosphere and freshly distilled prior to use. Dichloromethane and diethyl ether were purchased in p.a. quality from Aldrich. Zinc powder was purchased from Merck and used without further activation. Chlorotrimethylsilane was purchased from Acros and used as received. Titanocene dichloride was purchased from Alfa Aesar and used as received. All other chemicals were purchased from Aldrich and used without further purification. An IKAmag temperature modulator in combination with an oil bath or stainless-steel heating block was used to control the reaction temperatures. Thin-layer chromatography (TLC) was performed using E. Merck silica gel 60 F254 precoated plates (0.25 mm) and visualized by UV fluorescence quenching or staining ($KMnO_4$ or anisaldehyde). In general, Macherey- Nagel Silica gel 60 (particle size 0.04–0.063 mm) was used for flash chromatography. ^{1}H, ^{19}F and ^{13}C NMR spectra were recorded on a Bruker DRX 500 (500 MHz and 125 MHz, ^{1}H, ^{13}C, 2D-spectra, measurements at varied temperature), a Bruker Avance II 400 (400.1 MHz and 100.6 MHz, ^{1}H, ^{13}C, 2D- spectra), a Bruker Avance III 300 (300 MHz, ^{1}H) and reported to $CDCl_3$ [$\delta(^{1}H)$ = 7.26 ppm and $\delta(^{13}C)$ = 77.16 ppm] and C_6D_6 [$\delta(^{1}H)$ = 7.16 ppm and $\delta(^{13}C)$ = 128.06 ppm]. The following abbreviations were used: s = singlet, d = doublet, t = triplet, q = quartet, br = broad signal. NMR spectra were recorded at room temperature (298–300 K) unless noted otherwise. IR spectra were recorded on a Thermo Scientific Nicolet iS10 FT-IR spectrometer equipped with a diamond ATR unit and are reported in frequency of absorption. Low- and high-resolution mass analyses were performed by the service department at the Institute for Organic Chemistry, University of Freiburg using a Thermo Finnigan TSQ 700 for electron impact ionization (EI) at 70 eV, 200 °C. High resolution mass analyses (HRMS) were carried out on a Thermo Exactive with Orbitrap-Analyzer using atmospheric pressure chemical ionization (APCI) or electrospray ionization (ESI). Reversed-Phase chromatography was carried out on an Interchim Puriflash system equipped with a reversed phase silica gel column (type PF-15C18H/35G, 15µm particle size) eluting with acetonitrile (CH_3CN)/water. High Performance Ion Chromatography (HPIC) was carried out on a Thermo Scientific Dionex ICS-5000^{+} RFIC system using a dual pump equipped with a 10 µL injector loop, a Dionex CarboPac AG-11 guard column (2 × 50 mm) and a Dionex CarboPac AS-11 analytical column (4 × 250 mm). Eluent: Potassium hydroxide: Electrolytically generated in-line using H_2O (18 MΩ * cm); Eluent source: KOH-EGC cartridge; 10 µl sample were injected (flow rate 0.25 ml/min). KOH isocratic: 7 mM. Suppressor: 70 mM, 44 mA. The analytes were detected by suppressed conductivity detection.

Compound **1r** (racemic) was a generous gift by Christian Grugel (workgroup of Prof. Breit, University of Freiburg).[1]

Synthesis of the Precursors 1a–q, 3, 4, and 7

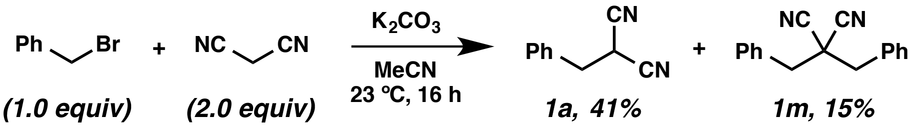

2-Benzylmalononitrile (1a)[2] **and 2,2-dibenzylmalononitrile (1m).**[3] In a 250 ml round bottom flask malononitrile (3.30 g, 50.0 mmol, 2.0 eq.) was dissolved in MeCN (120 ml). After the addition of K_2CO_3 (4.32 g, 31.3 mmol, 1.25 eq.) and benzyl bromide (25.0 mmol, 1.0 eq.) the mixture was stirred at room temperature for 16 h. Water (100 ml) was added and the mixture was transferred into a separation funnel. The aqueous layer was extracted with EtOAc (3 × 80 ml). The organic layer was dried over Na_2SO_4, filtered, and the solvent was removed under reduced pressure. The products were purified by column chromatography (*n*-pentane/Et_2O = 7:1 to 2:1). The double alkylated product (*n*-pentane /Et_2O = 1:1, R_f = 0.3), 980 mg, 3.66 mmol) was eluted first and was obtained in 15% yield. The monoalkylated product (*n*-pentane/Et_2O = 1:1, R_f = 0.2), 1.59 g, 10.2 mmol) was obtained second in 41% yield. The NMR data of **1a** and **1m** matched the literature values. **1a**: ^1H NMR ($CDCl_3$, 300 MHz): δ = 3.29 (d, *J* = 7.0 Hz, 2H), 3.91 (t, *J* = 7.0 Hz, 1H), 7.30-7.35 (m, 2H), 7.38-7.42 (m, 3H). **1m**: ^1H NMR ($CDCl_3$, 400 MHz): δ = 3.25 (s, 4H), 7.38-7.43 (m, 10H).

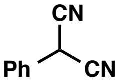

1b

2-Phenylmalononitrile.[4] Synthesized following a modified literature procedure.[5] In a flame-dried and argon filled 100 ml Schlenk flask K_2CO_3 (5.53 g, 40.0 mmol, 4.0 eq.), CuI (190 mg, 1.0 mmol, 10 mol%), L-proline (0.23 g, 2.0 mmol, 20 mol%) and malononitrile (1.98 g, 30.0 mmol, 3.0 eq.) were suspended in dry DMSO (40 ml). After the addition of iodobenzene (1.12 ml, 10.0 mmol, 1.0 eq.), the flask was sealed with a greased glass stopper and stirred for 16 h at 90 °C. After cooling to room temperature, the suspension was poured into aq. HCl (2 M, 200 ml). The aqueous layer was extracted with EtOAc (3 × 100 ml). The combined organic layers were washed with water (100 ml) and brine (5 × 50 ml), dried over Na_2SO_4, filtered, and the solvent was removed under reduced pressure. The product was purified by column chromatography (CH_2Cl_2, R_f = 0.8) and obtained as a colorless solid in 91% yield (1.28 g, 9.10 mmol). The NMR data matched the literature values. ^1H NMR ($CDCl_3$, 300 MHz): δ = 5.07 (s, 1H), 7.48-7.52 (m, 5H).

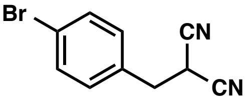

1c

2-(4-Bromobenzyl)malononitrile.[6] Synthesized in analogy to **1a** on a 10.0 mmol scale. Obtained as a colorless solid after column chromatography (CH_2Cl_2, R_f = 0.8) in 65% (1.52 g, 6.50 mmol) yield. The NMR data matched the literature values. ^1H NMR ($CDCl_3$, 300 MHz): δ = 3.24 (d, *J* = 6.8 Hz, 2H), 3.92 (t, *J* = 6.8 Hz, 1H), 7.19-7.22 (m, 2H), 7.53-7.56 (m, 2H).

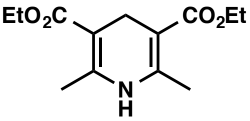

S1

Diethyl 2,6-dimethyl-1,4-dihydropyridine-3,5-dicarboxylate.[7] This compound was prepared according to the following procedure. In a 500 ml round bottom flask formaldehyde solution (37% in H_2O, 8.1 ml, 0.1 mol, 1.0 eq.) and NH_4OAc (15.4 g, 200 mmol, 2.0 eq.) were dissolved in water (200 ml). After addition of ethyl acetoacetate (51 ml, 0.40 mol, 4.0 eq.) the mixture was refluxed for 2 hours. After cooling to room temperature, the flask was immersed in an ice bath and stirring was continued for additional 30 min. The yellow precipitate was filtered off and washed three times with the mother liquor. The product (24.0 g, 94.1 mmol) was obtained after

recrystallization from H_2O/EtOH in 95% yield. The NMR data matched the literature values. ^{1}H NMR ($CDCl_3$, 300 MHz): δ = 1.27 (t, J = 14.2 Hz, 6H), 2.18 (s, 6H), 3.25 (s, 2H), 4.16 (q, J = 7.1 Hz, 4H), 5.23 (br, 1H).

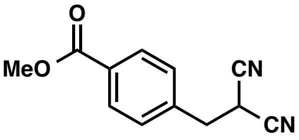

1d

Methyl 4-(2,2-dicyanoethyl)benzoate. Synthesized according to a literature procedure as follows.[8] In a 25 ml round bottom flask malononitrile (347 mg, 5.25 mmol, 1.05 eq.) and dihydropyridine **S1** (1.29 g, 5.1 mmol, 1.02 eq.) were dissolved in MeOH. To this suspension was added methyl 4-formylbenzoate (821 mg, 5.00 mmol, 1.0 eq.) in MeOH (5 ml). The yellow suspension was stirred at room temperature until it became a yellow solution. The solution was then concentrated to dryness under reduced pressure. The product was purified by column chromatography (*n*-pentane/Et_2O, 1:1, R_f = 0.2) and obtained as a colorless solid in 71% yield (870 mg, 3.56 mmol). ^{1}H NMR ($CDCl_3$, 400 MHz): δ = 3.35 (d, J = 6.9 Hz, 2H), 3.93 (s, 3H), 3.96 (t, J = 6.9 Hz, 1H), 7.40-7.43 (m, 2H), 8.07-8.10 (m, 2H). ^{13}C NMR ($CDCl_3$, 100 MHz): δ = 24.74, 36.68, 52.44, 111.94, 129.40, 130.70, 130.97, 137.79, 166.47. MS (EI, 70 eV): m/z (%) = 214.1 $[M]^+$ (12), 173.1 (5), 172.1 (38), 107.1 (100), 7.1 (7), 43.0 (17). HRMS (neg. ESI) calcd for $C_{12}H_9O_2N_2$ $[M-H]^-$: 213.0670, found 213.0671. IR (ATR): ν [cm^{-1}] = 2909, 1716, 1612, 1437, 1418, 1310, 1279, 1181, 1111, 1103, 1021, 965, 865, 760, 702.

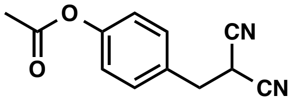

1e

4-(2,2-Dicyanoethyl)phenyl acetate. Synthesized in analogy to **1d**. The product was purified by column chromatography (*n*-pentane/Et_2O, 1:1, R_f = 0.2) and obtained as a colorless solid in 91% yield (977 mg, 4.56 mmol). ^{1}H NMR ($CDCl_3$, 500 MHz): δ = 2.31 (s, 3H), 3.29 (d, J = 6.9 Hz, 2H), 3.89 (t, J = 6.9 Hz, 1H), 7.13-7.16 (m, 2H), 7.33-7.36 (m, 2H). ^{13}C NMR ($CDCl_3$, 125.1 MHz): δ = 21.25, 25.09, 36.36, 112.14, 122.69, 130.69, 130.54, 151.23, 169.28. MS (EI, 70 eV): m/z (%) = 214.0 $[M]^+$ (23), 183.0 (50), 149.1 (100), 121.1 (19), 118.1 (26), 90.1 (18). HRMS (neg. ESI) calcd for $C_{11}H_9N_2S$ $[M-H]^-$: 213.0670, found 213.0671. IR (ATR): ν [cm^{-1}] = 2916, 1751, 1605, 1509, 1421, 1370, 1194, 1168, 1107, 1019, 913, 854, 804, 659, 625, 595.

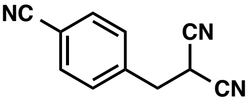

1f

2-(4-Cyanobenzyl)malononitrile.[3] Synthesized in analogy to **1d**. The product was purified by column chromatography (*n*-pentane/Et_2O, 1:1, R_f = 0.2) and obtained as a colorless solid in 73% yield (665 mg, 3.67 mmol). The NMR data matched the literature values. ^{1}H NMR ($CDCl_3$, 400 MHz): δ = 3.35 (d, J = 6.6 Hz, 2H), 4.00 (t, J = 6.7 Hz, 1H), 7.46-7.50 (m, 2H), 7.70-7.75 (m, 2H).

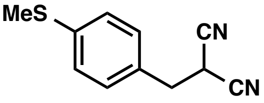

1g

2-(4-(Methylthio)benzyl)malononitrile. Synthesized in analogy to **1d**. The product was purified by column chromatography (*n*-pentane/Et_2O, 1:1, R_f = 0.4) and obtained as a colorless solid in 41% yield (413 mg, 2.04 mmol). ^{1}H NMR ($CDCl_3$, 400 MHz): δ = 2.49 (s, 3H), 3.25 (d, J = 6.8 Hz, 2H), 3.88 (t, J = 6.8 Hz, 1H), 7.22-7.25 (m, 2H), 7.26-7.29 (m, 2H). ^{13}C NMR ($CDCl_3$, 100 MHz): δ = 15.65, 25.15, 36.46, 112.23, 127.11,129.50, 129.68, 140.00. MS (EI, 70 eV): m/z (%) = 202.1 $[M]^+$, (45), 137.1 (199), 122.1 (23), 78.1 (5). HRMS (neg. APCI) calcd for $C_{11}H_9N_2S$

[M-H]⁻: 201.0492, found 201.0491. IR (ATR): ν [cm^{-1}] = 2923, 2257, 1601, 1495, 1453, 1436, 1426, 1408, 1314, 1294, 1262, 1193, 1163, 1112, 1093, 1029, 1017, 972, 956, 841, 824, 796, 715, 663, 564.

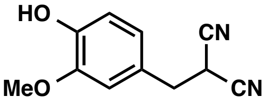

1h

2-(4-Hydroxy-3-methoxybenzyl)malononitrile.[9] Synthesized in analogy to **1d**. The product was purified by column chromatography (*n*-pentane/Et_2O, 1:1, R_f = 0.2) and obtained as a colorless solid in 91% yield (923 mg, 4.57 mmol). The NMR data matched the literature values. ^{1}H NMR (acetone-d_6, 500 MHz): δ = 3.34 (d, *J* = 7.0 Hz, 2H), 3.85 (s, 3H), 4.71 (t, *J* = 7.0 Hz, 1H), 6.84 (d, *J* = 8.1 Hz, 1H), 6.89 (dd, *J* = 2.1, 8.1 Hz, 1H), 7.07 (d, *J* = 2.0 Hz, 1H), 7.70 (s, 1H).

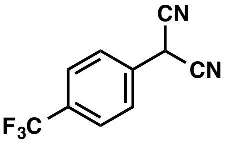

1i

2-(4-(Trifluoromethyl)phenyl)malononitrile.[10] Synthesized in analogy to **1b**. The product was purified by column chromatography (CH_2Cl_2, R_f = 0.8) and additional Kugelrohr apparatus (120 °C, 4 h) and then obtained as a colorless solid in 20% yield (420 mg, 2.0 mmol). The NMR data matched the literature values. ^{1}H NMR ($CDCl_3$, 300 MHz): δ = 5.15 (s, 1H), 7.69 (d, *J* = 8.8 Hz, 2H), 7.79 (d, *J* = 8.2 Hz, 2H).

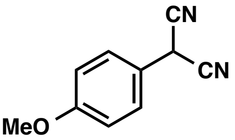

1j

2-(4-Methoxyphenyl)malononitrile.[11] Synthesized in analogy to **1b**. The product was purified by column chromatography (CH_2Cl_2, R_f = 0.8) and was obtained as a pink solid in 41% yield (796 mg, 4.63 mmol). The NMR data matched the literature values. ^{1}H NMR ($CDCl_3$, 400 MHz): δ = 3.83 (s, 3H), 5.00 (s, 1H), 6.97-7.01 (m, 2H), 7.38-7.42 (m, 2H).

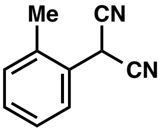

1k

2-(*o*-Tolyl)malononitrile.[12] The literature procedure was modified as follows. A flame-dried and argon-filled 10 ml Schlenk tube equipped with a magnetic stir bar was charged with $PdCl_2$ (7 mg, 0.04 mmol, 1 mol%) and PPh_3 (32 mg, 0.12 mmol, 3 mol%). The solid was suspended in dry Pyridine (2 ml) and stirred for 15 min at 60 °C. In a second flame-dried and argon filled 25 ml Schlenk tube equipped with a magnetic stir bar, NaH (60 % dispersion in mineral oil, 416 mg, 10.4 mmol, 2.6 eq.) was suspended in dry Pyridine (10 ml). Malononitrile (330 mg, 5.0 mmol, 1.25 eq.) was slowly added to this Schlenk tube. After complete addition of the malononitrile, the pre-generated catalyst was added to the 25 ml Schlenk tube. 2-Bromotoluene (481 µl, 4.0 mmol, 1.0 eq.) was added and the reaction was stirred at 85 °C for 48 h. After allowing the reaction mixture to cool down to room temperature, aq. HCl (1 M, 10 ml) was added and the mixture was transferred to a separation funnel containing aq. HCl (1 M, 50 ml). The aqueous layer was extracted with EtOAc (3 × 30 ml). The combined organic layers were dried over Na_2SO_4, filtered, and the solvent was removed under reduced pressure. The product was purified by column chromatography (*n*-pentane/Et_2O, 1:1, R_f = 0.8) and was obtained as a colorless solid in 92% yield (577 mg, 3.70 mmol). The NMR data matched the literature values.

^{1}H NMR ($CDCl_3$, 500 MHz): δ = 2.48 (s, 3H), 5.04 (s, 1H), 7.30-7.36 (m, 2H), 7.40 (ddd, *J* = 1.5, 7.5, 7.5 Hz, 1H), 7.54 (dd, *J* = 1.1, 7.7 Hz, 1H).

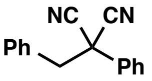

1l

2-benzyl-2-phenylmalononitrile.[13] In a 50 ml round bottom flask phenyl malononitrile (781 mg, 5.0 mmol, 1.0 eq.) was dissolved in CH_3CN. After the addition of K_2CO_3 (898 mg, 6.50 mmol, 1.30 eq.) and benzyl bromide (772 μl, 6.5 mmol, 1.3 eq.) the mixture was stirred at room temperature for 16 h. Water (50 ml) was added and the mixture was transferred into a separation funnel. The aqueous layer was extracted with EtOAc (3 × 20 ml). The organic layer was dried over Na_2SO_4, filtered, and the solvent was removed under reduced pressure. The product was purified by column chromatography (*n*-pentane/Et_2O = 5:1, R_f = 0.5) and was obtained as a colorless solid in 88% yield (1.03 g, 4.42 mmol). The NMR data matched the literature values. ^{1}H NMR ($CDCl_3$, 400 MHz): δ = 3.47 (s, 2H), 7.14-7.17 (m, 2H), 7.30-7.38 (m, 3H), 7.45-7.49 (m, 5H).

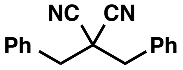

1m

Obtained from the synthesis of **1a** (see above).

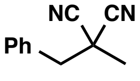

1n

2-Benzyl-2-methylmalononitrile.[14] Prepared in analogy to **1l** (5 mmol scale) using methyl iodide (620 μl, 10.0 mmol, 2.0 eq.) as alkylating agent. The product was purified by column chromatography (*n*-pentane/Et_2O, 3:1, R_f = 0.5) and was obtained as a colorless solid in 87% yield (740 mg, 4.35 mmol). The NMR data matched the literature values. ^{1}H NMR ($CDCl_3$, 400.1 MHz): δ = 1.80 (s, 3H), 3.21 (s, 2H), 7.36-7.42 (m, 5H).

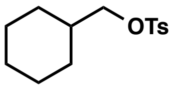

S2

Cyclohexylmethyl 4-methylbenzenesulfonate.[15] In a 50 ml round bottom flask equipped with a magnetic stir bar, cyclohexylmethanol (1.2 ml, 9.8 mmol, 1.0 eq.) was dissolved in CH_2Cl_2 (30 ml) and stirring was stated. After addition of Et_3N (2.1 ml, 15.0 mmol, 1.5 eq.) the solution was cooled to 0 °C. *para*-Toluenesulfonyl chloride (2.29 g, 12.0 mmol, 1.2 eq.) was added and the mixture was allowed to warm to room temperature and then stirred for an additional 18 h. Aqueous sat. NH_4Cl-solution (20 ml) was added and the solution transferred into a separation funnel. The layers were separated and the aqueous layer was extracted with CH_2Cl_2 (3 × 20 ml). The combined organic layers were dried over Na_2SO_4, filtered, and the solvent was removed under reduced pressure. The crude product was re-dissolved in CH_2Cl_2 (25 ml), pyridine (25 ml) and torn filter paper (1.9 g) was added and the mixture was sonicated in an ultrasonic bath for 2 h to remove any remaining tosyl chloride.[16] The mixture was filtered, the filtrate diluted with CH_2Cl_2 (150 ml) and washed with HCl (3 M, 5 × 100 ml). The organic layer was dried over Na_2SO_4, filtered, and the solvent was removed under reduced pressure. The product was obtained as a colorless solid in 78% yield (2.05 g, 7.65 mmol). The NMR data matched the literature values. ^{1}H NMR ($CDCl_3$, 300 MHz): δ = 0.83-0.97 (m, 2H), 1.07-1.28 (m, 3H), 1.58-1.72 (m, 6H), 2.45 (s, 3H), 3.80 (d, *J* = 6.2 Hz, 2H), 7.32-7.36 (m, 2H), 7.76-7.80 (m, 2H).

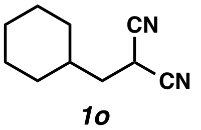

1o

2-(Cyclohexylmethyl)malononitrile. A flame-dried and argon-filled 10 ml Schlenk tube equipped with a magnetic stir bar was charged with NaH (95%, 183 mg, 7.65 mmol, 1.0 eq.) and the solid was suspended in fresh destilled THF (5 ml). While stirring, malononitrile (505 mg, 7.65 mmol, 1.0 eq.) in THF (10 ml) was added carefully at 0 °C. Tosylate **S2** (2.05 g, 7.65 mmol, 1.0 eq.) in THF (5 ml) was added and the mixture was heated to 60 °C and stirred for 24 h. The reaction was allowed to cool to room temperature and water (20 ml) was added. The mixture was transferred into a separation funnel containing water (100 ml). The organic layer was extracted with EtOAc (3 × 50 ml). The combined organic layers were dried over Na_2SO_4, filtered, and the solvent was removed under reduced pressure. The product was purified by column chromatography (*n*-pentane/Et_2O = 5:1, R_f = 0.3) and obtained as a colorless solid in 23% yield (280 mg, 1.73 mmol). The product still contained 2% of **S2**, which could not be removed by chromatography. It was used as such in the decyanation reaction. ^1H NMR ($CDCl_3$, 400 MHz): δ = 0.94-1.03 (m, 2H), 1.11-1.22 (m, 1H), 1.24-1.35 (m, 2H), 1.56-1.78 (m, 6H), 1.93 (t, *J* = 7.6 Hz, 2H), 3.75 (t, *J* = 7.7 Hz, 1H). ^{13}C NMR ($CDCl_3$, 100 MHz): δ = 20.39, 25.74, 32.74, 35.20, 37.95, 112.97, 127.99, 129.89. MS (EI, 70 eV): m/z (%) = 120.1 (6), 108.0 (31), 80.0 (39), 55.0 (100), 41.0 (18). HRMS (neg. ESI) calcd for $C_{10}H_{13}N_2$ $[M-H]^-$: 161.1084, found 161.1084. IR (ATR): ν [cm^{-1}] = 4924, 2854, 2256, 1450, 1353, 1274, 1239, 1189, 1174, 1134, 1098, 1019, 996, 948, 914, 894, 845, 666, 597, 590, 575, 555.

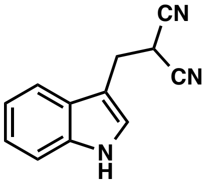

1p

2-((Indol-3-yl)methyl)malononitrile.[17] Synthesized in analogy to **1d**. The product was purified by column chromatography ($CHCl_3$, R_f = 0.2) and obtained as a colorless solid in 36% yield (276 mg, 1.73 mmol). The NMR data matched the literature values. ^1H NMR ($CDCl_3$, 500 MHz): δ = 3.51 (dd, *J* = 0.7, 7.1 Hz, 2H), 3.95 (t, *J* = 7.1 Hz, 1H), 7.20 (ddd, *J* = 1.0, 7.0, 7.0 Hz, 1H), 7.25-7.30 (m, 2H), 7.42 (dt, *J* = 1.0, 1.0, 8.1 Hz, 1H), 7.57-7.70 (m, 1H), 8.24 (br, 1H).

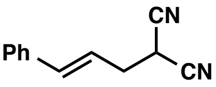

1q

2-Cinnamylmalononitrile.[18] Synthesized in analogy to **1d**. The product was purified by column chromatography (*n*-pentane/Et_2O, 1:1, R_f = 0.5) and obtained as a colorless solid in 56% yield (547 mg, 3.00 mmol). The NMR data matched the literature values. ^1H NMR ($CDCl_3$, 400 MHz): δ = 2.93 (ddd, *J* = 1.3, 6.7, 7.4 Hz, 2H), 3.82 (t, *J* = 6.6 Hz, 1H), 6.18 (dt, *J* = 7.4, 15.7 Hz, 1H), 6.71 (dt, *J* = 1.4, 15.8 Hz, 1H), 7.27-7.44 (m, 5H).

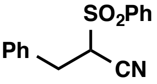

3

***rac*-3-Phenyl-2-(phenylsulfonyl)propanenitrile**.[19] Synthesized in analogy to **1a** on a 10.0 mmol scale using (phenylsulfonyl)acetonitrile instead of malononitrile. Obtained as a colorless solid after column chromatography (*n*-pentane/Et_2O = 10:9, R_f = 0.6) in 45% yield (1.23 g, 4.54 mmol). The NMR data matched the literature values. ^1H NMR ($CDCl_3$, 400 MHz): δ = 3.10 (dd, *J* = 11.8, 13.6 Hz, 1H), 3.59 (dd, *J* = 3.8, 13.6 Hz, 1H), 4.07 (dd, *J* = 3.8, 11.8 Hz, 1H), 7.25-7.37 (m, 5H), 7.64-7.69 (m, 2H), 7.79 (dddd, *J* = 1.3, 1.3, 7.5, 7.5 Hz, 1H), 8.05-8.08 (m, 2H).

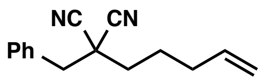

4

2-Benzyl-2-(pent-4-en-1-yl)malononitrile. Synthesized following the same procedure as for 2-benzyl-2-methylmalononitrile (**1n**), but on a 3.0 mmol scale and with 5-bromo-pentene as alkylation reagent. The product was purified by column chromatography (*n*-pentane/Et_2O = 4:1, R_f = 0.6) and obtained as a colorless solid in 24% yield (160 mg, 0.71 mmol). ^{1}H NMR ($CDCl_3$, 400 MHz): δ = 1.80-1.87 (m, 2H), 1.93-1.98 (m, 2H), 2.18 (qt, *J* = 1.3, 7.0 Hz, 2H), 3.21 (s, 2H), 5.03-5.10 (m, 2H), 5.78 (ddt, *J* = 3.1, 6.7, 10.3 Hz, 1H), 7.36-7.43 (m, 5H). ^{13}C NMR ($CDCl_3$, 100 MHz): δ = 24.88, 32.77, 37.03, 39.45, 43.60, 115.44, 116.04, 128.94, 129.11, 130.34, 132.16, 136.71. MS (EI, 70 eV): m/z (%) = 224.1 $[M]^+$ (5), 196.1 (6), 91.0 (100). HRMS (pos. APCI) calcd for $C_{15}H_{20}N_3$ $[M+NH_4]^+$: 242.1652, found 242.1652. IR (ATR): ν [cm^{-1}] = 3070, 3036, 2928, 2863, 2249, 1644, 1497, 1460, 1448, 1422, 1344, 1247, 1143, 1082, 1033, 998, 917, 824, 766, 745, 697, 667, 635, 584, 572, 564.

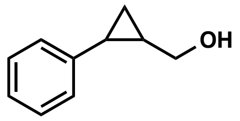

S3

(2-Phenylcyclopropyl)methanol. This compound was synthesized according to a literature procedure as follows.[20] To a flame dried and argon backfilled 500 ml Schlenk flask were added CH_2I_2 (2.6ml, 31.2 mmol, 2.0 eq.) and dry dichloromethane (90 ml). To a second flame dried and argon backfilled 100 ml Schlenk flask were added cinnamyl alcohol (2.0 ml, 15.6 mmol, 1.0 eq.) and dry DCM (40 ml). After cooling both Schlenk flasks to 0 °C, to each flask was added Et_2Zn-Solution (1 M in Hexane, 19.6 mmol, 1.25 eq.) dropwise. The Schlenk flasks were stirred for further 30 min at 0 °C and then the contents of the 100 ml Schlenk flask was transferred to the 500 ml Schlenk flask via cannula transfer. The reaction mixture was stirred at 0 °C for 30 min. Then, the ice bath was removed and the reaction was stirred for 16 h at room temperature. Saturated aq. NH_4Cl-solution (50 ml) and HCl (2 M, 200 ml) were added. The mixture was transferred to a separation funnel, the layers were separated and the aqueous layer was extracted with dichloromethane (2 × 200 ml). The combined organic layers were dried over $MgSO_4$, filtered, and concentrated to dryness under reduced pressure. The product (2.3 g, 15.6 mmol) was obtained in analytical pure form and quantitative yield (>99%), and it was used without purification. The NMR data matched the literature values.[21] ^{1}H NMR (300 MHz, $CDCl_3$): δ = 0.90-1.02 (m, 2H), 1.41-1.52 (m, 1H), 1.57 (bs, 1H), 1.80-1.86 (m, 1H), 3.63 (dd, *J* = 3.0, 6.7 Hz, 2H), /7.05-7.09 (m, 2H), 7.12-7.18 (m, 1H), 7.23-2.29 (m, 2H).

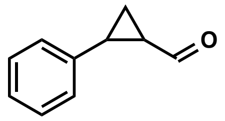

S4

2-Phenylcyclopropane-1-carbaldehyde. This compound was synthesized according to a literature procedure as follows.[20] To a flame dried and argon backfilled 100 ml Schlenk flask were added (2-phenylcyclopropyl)methanol (**S3**) (2.3 g, 15.6 mmol, 1.0 eq.), dry dichloromethane (40 ml) and pyridinium chlorochromate (5.04 g, 23.4 mmol, 1.5 eq.). The reaction was stirred for 1 h at room temperature and then filtered over celite. The filtrate was dried under reduced pressure and the product (2.01 g, 13.8 mmol) was obtained after column chromatography (CH_2Cl_2, R_f = 0.9) in 89% yield. The NMR data matched the literature values.[22] ^{1}H NMR (300 MHz, $CDCl_3$): δ = 1.53 (ddd, *J* = 4.9, 6.7, 8.2 Hz, 1H), 1.73 (dt, *J* = 5.0, 9.2 Hz, 1H), 2.14-2.21 (m, 1H), 2.63 (ddd, *J* = 4.0, 6.7, 9.3 Hz, 1H), 7.09-7.10 (m, 2H), 7.13-7.16 (m, 1H), 7.20-7.23 (m, 2H), 9.33 (d, *J* = 4.6 Hz, 1H).

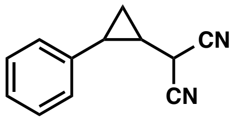

7

2-(2-Phenylcyclopropyl)malononitrile. This compound was synthesized according to a modified literature procedure for a different compound.[23] In a 100 ml round bottom flask 2-phenylcyclopropane-1-carbaldehyde (**S4**) (585 mg, 4.0 mmol, 1.0 eq.) was dissolved in acetonitrile (4.76 ml). *p*-Toluenesulfonyl chloride (877 mg, 4.60 mmol, 1.15 eq.) was added and the solution was cooled to –10 °C. An aqueous solution of KCN (432 mg, 6.64 mmol, 1.66 mmol) in H_2O (2.4 ml) was added dropwise. The solution was stirred at –10 °C for 30 min and then warmed to room temperature. The mixture was stirred until full conversion as monitored by ^{1}H NMR analysis of reaction samples. After full conversion was achieved, the reaction mixture was filtered over celite, the filter cake was rinsed with dichloromethane and the resulting filtrate was dried over $MgSO_4$. The solvent was removed under reduced pressure at 15 °C water bath temperature. The resulting crude tosylated cyanohydrin was used without further purification due to its tendency to decompose over time and during attempted flash chromatography.
In a 100 ml round bottom flask, 18-crown-6 (2.11 g, 8.0 mmol, 2.0 eq.) was dissolved in DMSO (5 ml). Potassium cyanide (520 mg, 8.0 mmol, 2.0 eq.) was added and the mixture was stirred at room temperature until all solids had dissolved. In a 10 ml vial that was cooled by an ice bath, the tosylated cyanohydrin was dissolved in DMSO (1 ml). Since the dissolving of the tosylated cyanohydrin was exothermic, cooling the vial was required to prevent premature decomposition of the substrate. The resulting solution was then slowly added to the flask containing the 18-crown-6/KCN solution. The reaction was stirred at room temperature for 3 h. The reaction mixture was transferred to a separation funnel containing Et_2O (70 ml). After extraction, the organic layer was separated and washed seven times with brine (15 ml), dried over Na_2SO_4, filtered and concentrated to dryness under reduced pressure. The product (70 mg, 0.4 mmol) was obtained after column chromatography (*n*-Pentane:EtOAc = 11:1, R_f = 0.2) in 10% yield. ^{1}H NMR ($CDCl_3$, 400 MHz): δ = 1.28-1.36 (m, 2H), 1.71 (dddd, *J* = 4.5, 5.6, 6.4 8.2 Hz, 1H), 2.21-2.28 (m, 1H), 3.87 (d, *J* = 6.4 Hz, 1H), 7.11-7.14 (m, 2H), 7.22-7.26 (m, 1H), 7.29-7.34 (m, 2H). ^{13}C NMR ($CDCl_3$, 100 MHz): δ = 13.45, 20.75, 22.48, 26.32, 111.62, 126.55, 127.16, 128.86, 138.64. MS (EI, 70 eV): m/z (%) = 182.3 $[M]^+$ (15), 155.1 (26), 153.3 (26), 115.0 (100), 90.7 (17). 77.1 (12), 51.0 (13). HRMS (neg. APCI) calcd for $C_{12}H_9N_2$ $[M\text{-}H]^-$: 181.0771, found 181.0771. IR (ATR): ν [cm^{-1}] = 2976, 2959, 2475, 2108, 1733, 1456, 1446, 1402, 1391, 1363, 1159, 1063, 1028, 842, 792, 744, 716, 605, 585, 580.

Titanium Catalyzed Reductive Decyanation Reactions

General Catalytic Decyanation Procedure

A flame-dried and argon-filled 10 ml Schlenk tube equipped with a magnetic stir bar was charged with 2,4,6-collidine hydrochloride (78 mg, 0.50 mmol, 1.0 eq.), Zn (65 mg, 1.0 mmol, 2.0 eq.), $ZnCl_2$ (68 mg, 0.50 mmol, 1.0 eq.) and Cp_2TiCl_2 (12.5 mg, 0.05 mmol, 10 mol%). The tube was evacuated and back-filled with argon three times. Freshly distilled THF (1.0 ml) was added and the mixture was stirred for about one minute to allow the color to change from red to lime green to smurf blue. At this point the geminal dinitrile was added (0.5 mmol, 1.0 equiv) followed by TMSCl (188 μl, 1.5 mmol, 3.0 eq.) and the reaction vessel was sealed with a greased glass stopper. The reaction mixture was stirred at 35 °C for 24 h. After cooling to room temperature dichloromethane (1 ml) was added, the reaction mixture was filtered and concentrated to give the crude product. The product was purified by column chromatography as described.
Due to the presence of $ZnCl_2$, the ring-opening of THF was usually observed by crude ^{1}H NMR. However, this THF opening did not interfere with the catalysis.

Data for the Products 2a–r

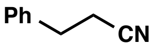

2a

3-Phenylpropanenitrile.[24] Synthesized according to the general catalytic decyanation procedure. The product was purified by column chromatography (*n*-pentane/Et_2O, 5:3, R_f = 0.6) and obtained as a colorless oil in 82% yield (53 mg, 0.41 mmol). The reaction was repeated on a 9.00 mmol scale (1.41 g of **1a**) and product **2a** was isolated in 70% yield (822 mg, 6.27 mmol). The NMR data matched the literature values. ^{1}H NMR ($CDCl_3$, 400 MHz): δ = 2.62 (t, *J* = 7.4 Hz, 2H), 2.96 (t, *J* = 7.4 Hz, 2H), 7.22-7.26 (m, 2H), 7.27-7.30 (m, 1H), 7.32-7.37 (m, 2H).

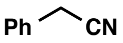

2b

2-Phenylacetonitrile.[25] Synthesized according to the general catalytic decyanation procedure. The product was purified by column chromatography (*n*-pentane/Et_2O, 5:1, R_f = 0.5) and obtained as a colorless oil in 74% yield (43 mg, 0.37 mmol). The NMR data matched the literature values. ^{1}H NMR ($CDCl_3$, 400 MHz): δ = 3.75 (s, 2H), 7.25-7.42 (m, 5H).

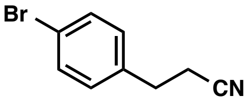

2c

3-(4-Bromophenyl)propannitrile.[26] Synthesized according to the general catalytic decyanation procedure. The product was purified by column chromatography (CH_2Cl_2, R_f = 0.8) and obtained as a colorless oil in 82% yield (85 mg, 0.41 mmol). The NMR data matched the literature values. ^{1}H NMR ($CDCl_3$, 300 MHz): δ = 2.61 (t, *J* = 7.3 Hz, 2H), 2.92 (t, *J* = 7.3 Hz, 2H), 7.13 (d, *J* = 8.4 Hz, 2H), 7.47 (d, *J* = 8.4 Hz, 2H).

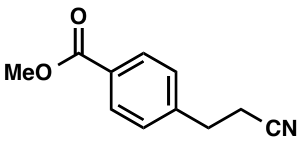

2d

Methyl 4-(2-cyanoethyl)benzoate. Synthesized according to the general catalytic decyanation procedure. The product was purified by column chromatography (*n*-pentane/Et_2O, 1:1, R_f = 0.4) and obtained as a colorless oil in 89% yield (84 mg, 0.44 mmol). ^{1}H NMR ($CDCl_3$, 400 MHz): δ = 2.65 (t, *J* = 7.3 Hz, 2H), 3.02 (t, *J* = 7.4 Hz, 2H), 3.91 (s, 3H), 7.29-7.33 (m, 2H), 8.00-8.03 (m, 2H). ^{13}C NMR ($CDCl_3$, 100 MHz): δ = 19.10, 31.64, 52.26, 118.77, 128.48, 129.48, 130.35, 143.20, 166.83. MS (EI, 70 eV): m/z (%) = 189.1 $[M]^+$ (51), 158.0 (100), 149.0 (66), 121.1 (21=, 103.1 (20), 89.0 (25), 78.1 (5). HRMS (pos. APCI) calcd for $C_{11}H_{12}O_2N$ $[M+H]^+$: 190.0863, found 190.0863. IR (ATR): ν [cm^{-1}] = 2954, 1718, 1613, 1435, 1311, 1281, 1182, 1112, 1021, 855, 768, 704, 582, 568, 563, 552.

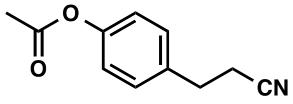

2e

4-(2-Cyanoethyl)phenyl acetate. Synthesized according to the general catalytic decyanation procedure. The product was purified by column chromatography (*n*-pentane/Et_2O, 1:1, R_f = 0.4) and obtained as a colorless oil in 56% yield (53 mg, 0.28 mmol). ^{1}H NMR ($CDCl_3$, 400 MHz): δ = 2.29 (s, 3H), 2.61 (t, *J* = 7.4 Hz, 2H), 2.96 (t, *J* = 7.4 Hz, 2H), 7.05-7.08 (m, 2H), 7.22-7.25 (m, 2H). ^{13}C NMR ($CDCl_3$, 100 MHz): δ = 19.46, 21.23, 31.16, 119.05, 122.15, 129.43, 135.70, 150.00, 169.52. MS (EI, 70 eV): m/z (%) = 189.1 $[M]^+$ (10), 147.1)30), 107.1 (100), 91.1 (6), 77.1 (12), 43.1 (16). HRMS (pos. ESI) calcd for $C_{11}H_{15}O_2N_2$ $[M+NH_4]^+$: 207.1128, found 207.1129. IR (ATR): ν [cm^{-1}] = 1755, 1508, 1425, 1369, 1217, 1194, 1167, 1104, 1019, 910, 851, 595, 576, 569, 559, 551.

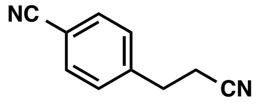

2f

4-(2-Cyanoethyl)benzonitrile. Synthesized according to the general catalytic decyanation procedure. The product was purified by column chromatography (*n*-pentane/Et_2O, 1:1, R_f = 0.2) and obtained as a colorless oil in 82% yield (64 mg, 0.41 mmol). ^{1}H NMR ($CDCl_3$, 500 MHz): δ = 2.66 (t, *J* = 7.2 Hz, 2H), 3.02 (t, *J* = 7.2 Hz, 2H), 7.35-7.38 (m, 2H), 7.63-7.66 (m, 2H). ^{13}C NMR ($CDCl_3$, 125 MHz): δ = 18.99, 31.62, 111.59, 118.44, 118.62, 129.32, 132.83, 143.30. MS (EI, 70 eV): m/z (%) = 156.1 $[M]^+$ (28), 116.0 (100), 89.1 (19), 63.1 (7). HRMS (pos. ESI) calcd for $C_{10}H_{12}ONS$ $[M+OH]^+$: 194.0634, found 194.0635. IR (ATR): ν [cm^{-1}] = 3066, 2955, 2867, 2245, 2228, 1610, 1507, 1450, 1423, 1298, 1274, 1180, 1115, 1025, 999, 840, 826, 761, 560.

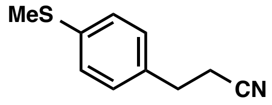

2g

3-(4-(Methylthio)phenyl)propanenitrile. Synthesized according to the general catalytic decyanation procedure. The product was purified by column chromatography (*n*-pentane/Et_2O, 3:1, R_f = 0.3) and obtained as a colorless oil in 60% yield (53 mg, 0.30 mmol). ^{1}H NMR ($CDCl_3$, 400 MHz): δ = 2.48 (s, 3H), 2.60 (t, *J* = 7.3 Hz, 2H), 2.92 (t, *J* = 7.3 Hz, 2H), 7.13-7.17 (m, 2H), 7.22-7.25 (m, 2H). ^{13}C NMR ($CDCl_3$, 100 MHz): δ = 16.11, 19.52, 31.22, 119.12, 127.35, 128.90, 135.62, 137.62. MS (EI, 70 eV): m/z (%) = 177.1 $[M]^+$ (63), 137.0 (100), 122.0 (17). HRMS (pos. ESI) calcd for $C_{10}H_{12}ONS$ $[M+OH]^+$: 194.0634, found 194.0635. IR (ATR): ν [cm^{-1}] = 2924, 2245, 1492, 1455, 1419, 1321, 1305, 1192, 1172, 1111, 1092, 1016, 982, 953, 942, 920, 846, 831, 801, 777, 723, 712, 628, 571.

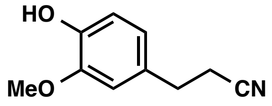

2h

3-(4-Hydroxy-3-methoxyphenyl)propanenitrile. Synthesized according to the general catalytic decyanation procedure. After cooling to room temperature TBAF-solution (1 M in THF, 2.5 ml, 5.0 eq.) was added and the mixture was stirred for further 15 minutes. Then water (5 ml) was added and the reaction mixture was stirred again for 15 minutes. The mixture was transferred into a separation funnel, water (15 ml) was added and the aqueous layer was extracted with CH_2Cl_2 (3 × 15 ml). The combined organic layers were dried over Na_2SO_4, filtered, and the solvent was removed under reduced pressure. The product was purified by column chromatography (*n*-pentane/Et_2O, 1:1, R_f = 0.2) and obtained as a colorless oil in 41% yield (36 mg, 0.20 mmol). ^{1}H NMR ($CDCl_3$, 400 MHz): δ = 2.59 (t, *J* = 7.3 Hz, 2H), 2.89 (t, *J* = 7.3 Hz, 2H), 3.90 (s, 3H), 5.55 (br, 1H), 6.70-6.75 (m, 2H), 6.87 (d, *J* = 8.0 Hz, 1H). ^{13}C NMR ($CDCl_3$, 100 MHz): δ = 19.91, 31.51, 56.11, 111.05, 114.83, 119.35, 121.13, 130.15, 145.00, 146.81. MS (EI, 70 eV): m/z (%) = 177.1 $[M]^+$ (73), 156.1 (13), 137.1 (100), 116.1 (43), 94.1 (9), 77.1 (8). HRMS (neg. ESI) calcd for $C_{10}H_{10}O_2N$ $[M-H]^-$: 176.0717, found 176.0717. IR (ATR): ν [cm^{-1}] = 2958, 1606, 1589, 1514, 1466, 1452, 1420, 1278, 1251, 1236, 1158, 1126, 1037, 905, 874, 844, 759, 691, 640, 610, 587, 577, 572, 567, 563, 559, 556, 551.

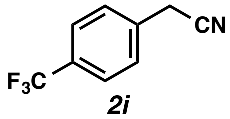

2i

2-(4-(Trifluoromethyl)phenyl)acetonitrile.[27] Synthesized according to the general catalytic decyanation procedure. The product was purified by column chromatography (*n*-pentane/Et_2O, 1:1, R_f = 0.5) and obtained as a colorless oil in 44% yield (40 mg, 0.22 mmol). The NMR data matched the literature values. ^{1}H NMR ($CDCl_3$, 400 MHz): δ = 3.83 (s, 2H), 7.46-7.49 (m, 2H), 7.65 (d, *J* = 8.1 Hz, 2H).

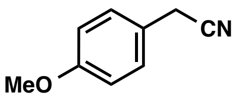

2j

2-(4-Methoxyphenyl)acetonitrile.[27] Synthesized according to the general catalytic decyanation procedure. The product was purified by column chromatography (*n*-pentane/Et_2O, 4:1, R_f = 0.4) and obtained as a colorless oil in 70% yield (51 mg, 0.35 mmol). The NMR data matched the literature values. ^1H NMR ($CDCl_3$, 500 MHz): δ = 3.68 (s, 2H), 3.81 (s, 3H), 6.88-6.92 (m, 2H), 7.22-7.26 (m, 2H).

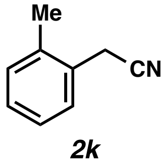

2k

2-(*o*-Tolyl)acetonitrile. [28] Synthesized according to the general catalytic decyanation procedure. The product was purified by column chromatography (*n*-pentane/Et_2O = 2:1, R_f = 0.5) and obtained as a colorless oil in 71% yield (47 mg, 0.35 mmol). The NMR data matched the literature values. ^1H NMR ($CDCl_3$, 400 MHz): δ = 2.36 (s, 3H), 3.67 (s, 2H), 7.22-7.25 (m, 2H), 7.26-7.29 (m, 1H), 7.36-7.39 (m, 1H).

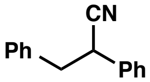

2l

***rac*-2,3-Diphenylpropanenitrile.**[29] Synthesized according to the general catalytic decyanation procedure with a reaction time of 48 h. The product was purified by reversed-phase flash column chromatography on C_{18}-modified silica gel eluting with MeCN/H_2O and was obtained as a colorless solid in 88% yield (91 mg, 0.44 mmol). The NMR data matched the literature values. ^1H NMR ($CDCl_3$, 300 MHz): δ = 3.13 (dd, J = 6.5, 7.1 Hz, 1H), 3.21 (dd, J = 5.4, 8.5 Hz, 1H), 4.00 (dd, J = 6.6, 8.2 Hz, 1H), 7.11-7.18 (m, 2H), 7.24-7.30 (m, 5H), 7.31-7.39 (m, 3H).

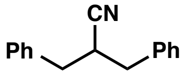

2m

2-Benzyl-3-phenylpropanenitrile.[3] Synthesized according to the general catalytic decyanation procedure with a reaction time of 48 h. The product was purified by column chromatography (*n*-pentane/Et_2O, 5:1, Rf = 0.5) and obtained as a pale-yellow oil in 66% yield (72 mg, 0.33 mmol). The NMR data matched the literature values. ^1H NMR ($CDCl_3$, 500 MHz): δ = 2.29 (d, J = 6.9 Hz, 4 H), 3.03 (tt, J = 6.9, 6.9 Hz, 1H), 7.23-7.31 (m, 6H), 7.33-7.37 (m, 4H).

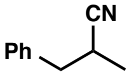

2n

***rac*-2-Methyl-3-phenylpropanenitrile.** [30] Synthesized according to the general catalytic decyanation procedure with a reaction time of 48 h. The product was purified by column chromatography (*n*-pentane/Et_2O, 5:2, Rf = 0.6) and obtained as a pale-yellow oil in 54% yield (38 mg, 0.27 mmol). The NMR data matched the literature values. ^1H NMR ($CDCl_3$, 500 MHz): δ = 1.33 (d, J = 6.9 Hz, 3H), 2.81-2.89 (m, 2H), 2.93-2.96 (m, 1H), 7.22-7.25 (m, 2H), 7.27-7.30 (m, 1H), 7.32-7.36 (m, 2H).

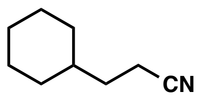

2o

3-Cyclohexylpropanenitrile.[31] Synthesized according to the general catalytic decyanation procedure. The product was purified by flash chromatography (*n*-pentane/Et_2O = 5:1, R_f = 0.5) and obtained as a colorless oil in 80% yield (55 mg, 0.40 mmol). The NMR data matched the literature values. ^{1}H-NMR ($CDCl_3$, 400.1 MHz): δ = 0.86-0.95 (m, 2H), 1.09-1.31 (m, 3H), 1.34-1.44 (m, 1H), 1.52-1.58 (m, 2H), 1.62-1.75 (m, 5H), 2.34 (t, *J* = 7.4 Hz, 2H).

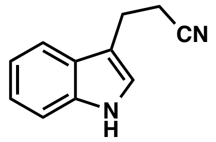

2p

3-(Indol-3-yl)propanenitrile.[32] Synthesized according to the general catalytic decyanation procedure. The product was purified by column chromatography ($CHCl_3$, 1:1, R_f = 0.2) and obtained as a colorless oil in 76% yield (65 mg, 0.38 mmol). The NMR data matched the literature values. ^{1}H NMR ($CDCl_3$, 400 MHz): δ = 2.71 (t, *J* = 7.2 Hz, 2H), 3.15 (td, *J* = 0.9, 7.2 Hz, 2H), 7.12-7.14 (m, 1H), 7.16 (ddd, *J* = 0. 9, 6.9, 6.9 Hz, 1H), 7.23 (ddd, *J* = 1.2, 7.2, 9.3 Hz, 1H), 7.39 (ddd, *J* = 0.9, 8.1, 8.1 Hz, 1H), 7.54-7.58 (m, 1H), 8.08 (br, 1H).

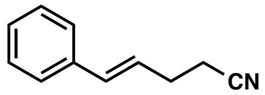

2q

(*E*)-5-Phenylpent-4-enenitrile.[33] Synthesized according to the general catalytic decyanation procedure. The product was purified by column chromatography (*n*-pentane/Et_2O, 2:1, Rf = 0.3) and obtained as a colorless oil in 72% yield (56 mg, 0.36 mmol). The NMR data matched the literature values. ^{1}H NMR ($CDCl_3$, 400 MHz): δ = 2.47-2.53 (m, 2H), 2.54-2.61 (m, 2H), 6.19 (dt, *J* = 6.7, 15.8 Hz, 1H), 6.53 (dt, *J* = 1.5, 15.8 Hz, 1H), 7.23-7.28 (m, 1H), 7.29-7.39 (m, 4H).

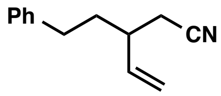

2r

3-Phenethylpent-4-enenitrile. Synthesized according to the general catalytic decyanation procedure with a reaction time of 48 h. The product was purified by column chromatography (*n*-pentane/Et_2O, 5:1, R_f = 0.4) and obtained as a colorless oil in 42% yield (38 mg, 0.21 mmol). ^{1}H NMR ($CDCl_3$, 400 MHz): δ = 1.72-1.81 (m, 1H), 1.83-1.91 (m, 1H), 1.35-2.45 (m, 3H), 2.55-2.62 (m, 1H), 2.66-2.74 (m, 1H), 5.18-5.25 (m, 2H), 5.68-5.77 (m, 1H), 7.16-7.19 (m, 2H), 7.20-7.23 (m, 1H), 7.27-7.32 (m, 2H). ^{13}C NMR ($CDCl_3$, 100 MHz): δ = 23.44, 33.13, 35.51, 39.74, 117.77, 118.33, 126.23, 128.49, 128.63, 138.60, 141.30. MS (EI, 70 eV): m/z (%) = 184.4 $[M]^+$ (7), 168.0 (9), 144.1 (12), 129.1 (17), 104.0 (50), 91.0 (100), 65.0 (35), 39.0 (5). HRMS (pos. APCI) calcd for $C_{13}H_{19}N_2$ $[M+NH_4]^+$: 203.1543, found 203.1545. IR (ATR): ν [cm^{-1}] = 3084, 3027, 2925, 2858, 2247, 1643, 1603, 1497, 1454, 1419, 1030, 994, 923, 749, 700, 624, 605, 596, 588, 583, 580, 574, 559.

Titanium Catalyzed Reductive Desulfonylation of 3

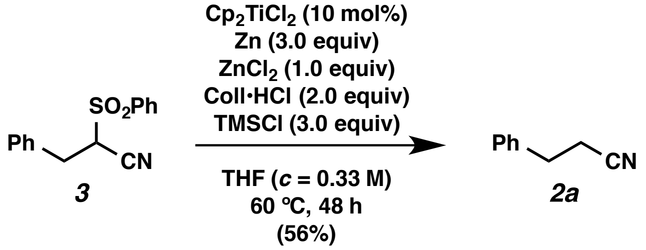

A flame-dried and argon-filled 10 ml Schlenk tube equipped with a magnetic stir bar was charged with 2,4,6-collidine hydrochloride (156 mg, 1.0 mmol, 2.0 eq.), Zn (98 mg, 1.5 mmol, 3.0 eq.), $ZnCl_2$ (68 mg, 0.5 mmol, 1.0 eq.) and Cp_2TiCl_2 (12.5 mg, 0.05 mmol, 10 mol%). The tube was evacuated and back-filled with argon three times. Freshly distilled THF (1.5 ml) was added and the mixture was stirred for about one minute to allow the color to change from red to lime green to smurf blue. At this point 3-phenyl-2-(phenylsulfonyl)propanenitrile (**3**) was added (0.5 mmol, 1.0 equiv) followed by TMSCl (188 μl, 1.5 mmol, 3.0 eq.) and the reaction vessel was sealed with a greased glass stopper. The reaction mixture was stirred at 60 °C for 48 h. After cooling to room temperature dichloromethane (1 ml) was added, the reaction mixture was filtered and concentrated to give the crude product. The product was purified by column chromatography (cyclohexane/ethyl acetate: 6:1, R_f = 0.3) and obtained as a colorless oil in 56% yield (37 mg, 0.28 mmol). The NMR data matched the literature values. ^{1}H NMR ($CDCl_3$, 400 MHz): δ = 2.62 (t, *J* = 7.4 Hz, 2H), 2.96 (t, *J* = 7.4 Hz, 2H), 7.22-7.26 (m, 2H), 7.27-7.30 (m, 1H), 7.32-7.37 (m, 2H).
No desulfonylation occurred in absence of the catalyst under otherwise identical conditions.

Negative Radical Clock Experiments and Data for 5 and 8

The decyanation reaction of **4** according to the general procedure with 10 mol% Cp_2TiCl_2 proceeded gave nitrile **5** as the sole product as confirmed by crude NMR.
The decyanation reaction of **7** according to the general procedure with 10 mol% Cp_2TiCl_2 and 48 h reaction time gave nitrile **8** as the sole product as confirmed by crude NMR. The remaining material was unreacted substrate.

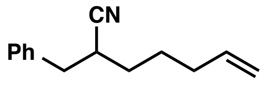

5

2-Benzylhept-6-enenitrile. Synthesized according to the general catalytic decyanation procedure. The product was purified by column chromatography (*n*-pentane/Et_2O, 20:1→4:1) (R_f = 0.4 in *n*-pentane/Et_2O, 10:1) and obtained as a colorless oil in 70% yield (71 mg, 0.35 mmol). ^{1}H NMR ($CDCl_3$, 400 MHz): δ = 1.52-1.76 (m, 4H), 2.04-2.15 (m, 2H), 2.74-2.81 (m, 1H), 2.83-2.96 (m, 2H), 4.97-5.05 (m, 2H), 5.77 (ddt, *J* = 6.8, 10.2, 16.9 Hz, 1H), 7.22-7.26 (m, 2H), 7.26-7.30 (m, 1H), 7.32-7.36 (m, 2H). ^{13}C NMR ($CDCl_3$, 100 MHz): δ = 26.39, 31.31, 33.17, 33.84, 38.59, 115.48, 121.81, 127.34, 128.85, 129.11, 137.16, 137.74. MS (EI, 70 eV): m/z (%) = 199.1 $[M]^+$ (9), 171.1 (11), 144.1 (8), 108.0 (6), 91.0 (100), 80.0 (5), 65.0 (5). HRMS (pos. APCI) calcd for $C_{14}H_{21}N_2$ $[M+NH_4]^+$: 217.1699, found 217.1701. IR (ATR): ν [cm^{-1}] = 3030, 2929, 2862, 2238, 1641, 1497, 1455, 1030, 995, 913, 739, 700, 642, 624, 606, 573, 566, 560, 556, 553.

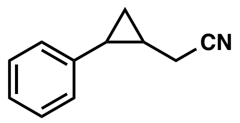

8

2-(2-Phenylcyclopropyl)acetonitrile: Synthesized according to the general catalytic decyanation procedure on a 0.2 mmol scale with 48 h reaction time. A ^{1}H NMR analysis of the

crude reaction mixture did not show any ring-opening products. The decyation product **8** was purified by column chromatography (*n*-pentane/EtOAc = 11:1, R_f = 0.3) and obtained as a colorless oil in 45% yield (14 mg, 0.09 mmol). A second fraction (R_f = 0.2) contained the unreacted substrate, which could be recovered in 41% yield (15 mg, 0.08 mmol). ^{1}H NMR ($CDCl_3$, 500 MHz): δ = 1.03 (dt, *J* = 5.6, 9.0 Hz, 1H), 1.11 (dt, *J* = 5.5, 8.4 Hz, 1H), 1.32-1.39 (m, 1H), 1.92 (ddd, *J* = 4.5, 5.4, 9.5 Hz, 1H), 2.58 (qd, *J* = 6.1, 17.3 Hz, 2H), 7.06-7.09 (m, 2H), 7.16-7.21 (m, 1H), 7.24-7.30 (m, 2H). ^{13}C NMR ($CDCl_3$, 125 MHz): δ = 14.94, 17.63, 21.58, 22.96, 118.26, 126.05, 126.24, 128.56, 141.09. MS (EI, 70 eV): m/z (%) = 157.1 $[M]^+$ (16), 128.9 (23), 117.1 (100), 91.0 (29), 77.3 (9), 65.1 (10), 39.1 (5). HRMS (pos. APCI) calcd for $C_{11}H_{11}N$ $[M+H]^+$: 158.0964, found 158.0964. IR (ATR): ν [cm^{-1}] = 2323, 2160, 2050, 1979, 1604, 1499, 1029, 754, 697, 632,624, 618, 613, 610, 587, 582, 563, 556, 553.

Deuteration Experiments

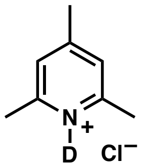

S5

2,4,6-Collidine deuterochloride (94% D).[34] In a flame-dried and argon-filled 50 ml round bottom Schlenk flask 2,4,6-collidine (2.65 ml, 20.0 mmol, 1.0 eq.) was dissolved in dry Et_2O (40 mL). CD_3OD (1.22 ml, 30.0 mmol, 1.5 eq.) was added and the solution was cooled to 0 °C. Acetyl chloride (1.57 ml, 22.0 mmol, 1.1 eq.) was slowly added and the mixture was then allowed to warm to room temperature. The resulting white solid was filtered off under inert conditions using a Schlenk frit. The solid was washed three times with dry Et_2O and then dried under reduced pressure. The deuteration grade was determined by ^{1}H NMR to be 94% as described earlier.[34]

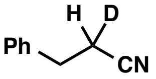

2a-d$_1$

3-Phenylpropanenitrile-2-d. Synthesized according to the general catalytic decyanation procedure from **1a** and using Coll•DCl (94% D) instead of Coll•HCl. The $ZnCl_2$ used in this experiment was dried again prior to use. The product was purified by column chromatography (*n*-pentane/Et_2O, 5:3, R_f = 0.6) and obtained as a colorless oil in 77% yield (51 mg, 0.39 mmol). The ^{1}H NMR data showed that a 68% deuterium incorporation had occurred during the reaction (72% transfer efficiency considering the deuteration grade of Coll•DCl). Repeating the experiment and quenching the reaction in addition with D_2O led to the identical deuterium incorporation. ^{1}H NMR ($CDCl_3$, 400 MHz): δ = 2.59-2.64 (m, 1.34H), 2.95-2.98 (m, 2H), 7.22-7.25 (m, 2H), 7.26-7.30 (m, 1H), 7.33-7.36 (m, 2H). ^{13}C NMR ($CDCl_3$, 100 MHz): δ = 19.24 (t, *J* = 20.8 Hz), 31.62, 119.23, 127.35, 128.36, 128.99, 138.16. MS (EI, 70 eV): m/z (%) = 132.1 $[M]^+$ (17), 91.0 (100), 65.1 (14), 39.0 (5). HRMS (pos. APCI) calcd for $C_9H_{12}{}^2HN_2$ $[M+NH_4]^+$: 150.1136, found 150.1135.

An experiment run with Coll•HCl and THF-d_8 showed no deuterium incorporation, ruling out a hydrogen atom transfer (HAT) from the solvent.

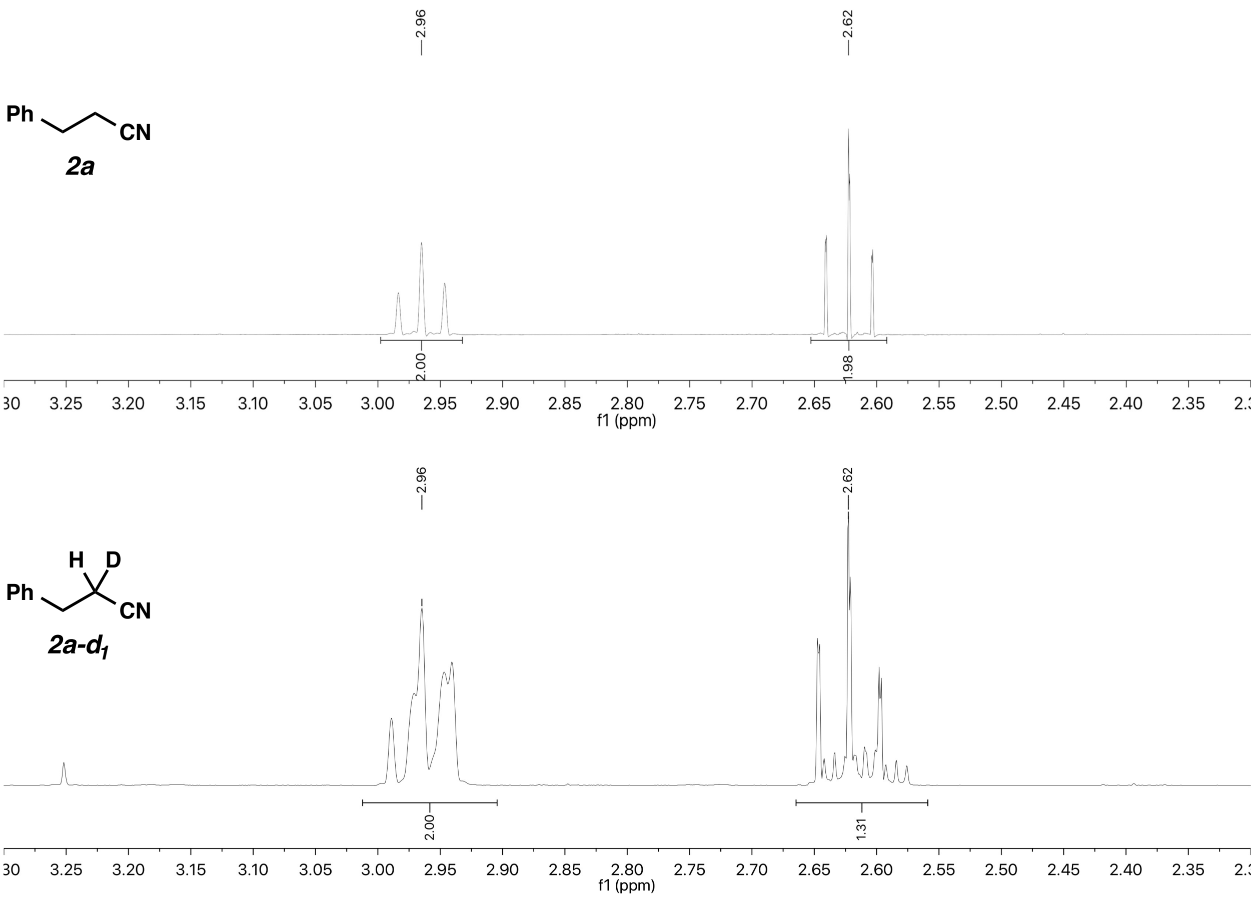

Figure S1. Excerpts of the NMR spectra of **2a** (top) and **2a–d₁** (bottom).

Cyclic Voltammetry

Cyclic voltammograms were recorded with a VersaSTAT 4 Potentiostat Galvanostat under moisture- and oxygen-free conditions. A standard three-electrode setup was employed, with a Pt millielectrode (model G0228, AMETEK) as working electrode. The counter electrode consisted of a platinum wire in an electrolyte (0.2 M Bu_4NPF_6 in THF), while the reference electrode consisted of a silver wire in a 3 M NaCl/sat. AgCl solution.

Experimental Procedure

Bu_4NPF_6 (387 mg, 1.00 mmol) was added to the electrochemical cell. The cell was closed and flushed with argon for 5–10 minutes. To a flame dried, argon filled 10 ml Schlenk tube were added Cp_2TiCl_2 (7.5 mg, 0.03 mmol, 1.0 eq.), Zn (39 mg, 0.60 mmol, 20 eq.), and depending on the experiment either Coll•HCl, $ZnCl_2$, or Coll•HCl and $ZnCl_2$ as additives in the following quantities: Coll•HCl (98 mg, 0.30 mmol, 10 eq.), $ZnCl_2$ (41 mg, 0.30 mmol, 10 eq.). The Schlenk tube was evacuated and backfilled with argon. THF (1.5 ml) was added and the mixture was stirred for 5 minutes. Then, THF (4.5 ml) was added to the electrochemical cell via syringe followed by 0.5 ml of the prepared solution of the catalyst-additive mixture. The resulting final concentration was 2 mM based on Zn-reduced Cp_2TiCl_2. The cyclic voltammetry measurements were carried out using a sweep rate of 0.1 V s^{-1}. At the end of each series of experiments, a small amount of ferrocene was added, the measurement was repeated. All potentials were reported against the ferrocenium/ ferrocene (Fc^+/Fc) redox couple.

The recorded voltammograms of Zn-reduced Cp_2TiCl_2 in presence of Coll•HCl, $ZnCl_2$, and Coll•HCl/$ZnCl_2$ are shown in Figure S2–Figure S4. In the manuscript, these were combined to Figure 2.

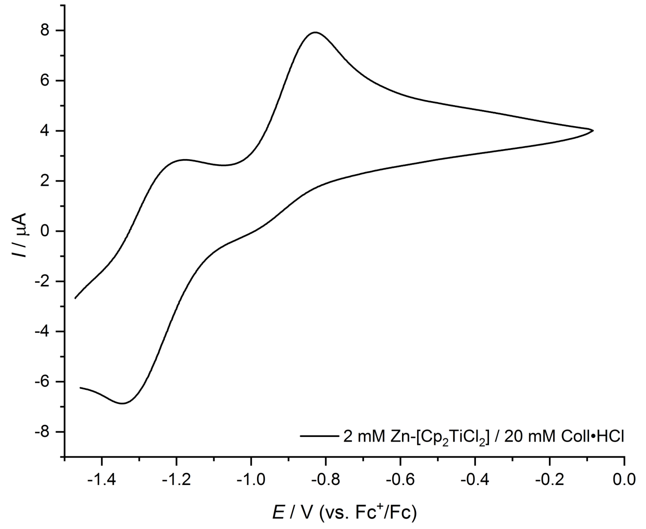

Figure S2. Cyclic voltammogram of zinc-reduced Cp_2TiCl_2 (c = 2 mM) with added Coll•HCl (c = 20 mM) at a sweep rate of 0.1 V s^{-1}.

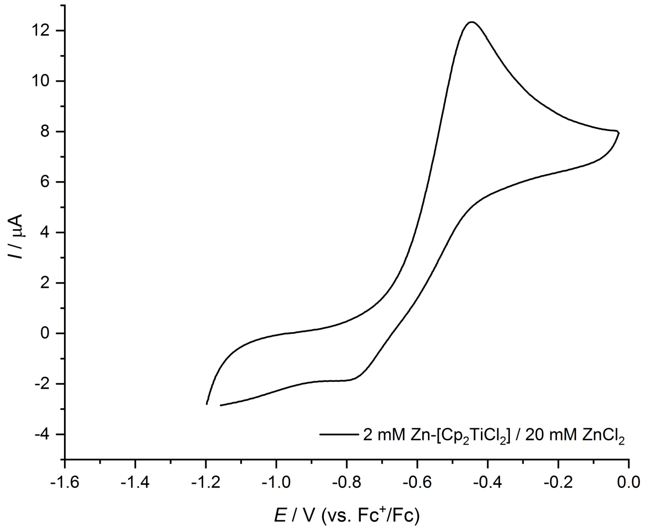

Figure S3. Cyclic voltammogram of zinc-reduced Cp_2TiCl_2 (c = 2 mM) with added $ZnCl_2$ (c = 20 mM) at a sweep rate of 0.1 V s^{-1}.

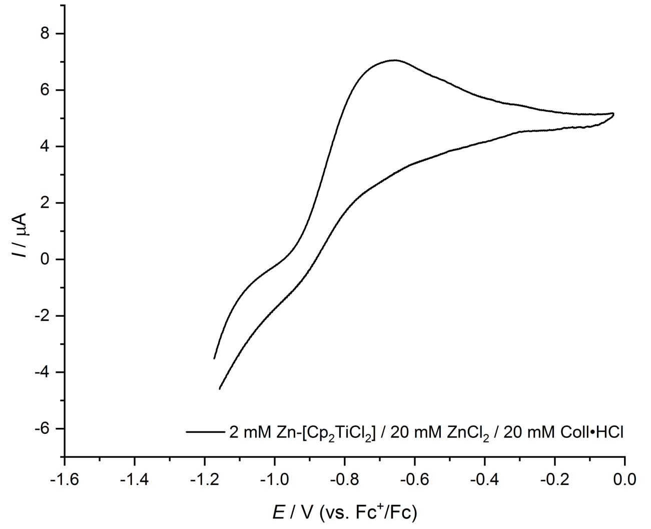

Figure S4. Cyclic voltammogram of zinc-reduced Cp_2TiCl_2 (c = 2 mM) with added Coll•HCl (c = 20 mM) and $ZnCl_2$ (c = 20 mM) at a sweep rate of 0.1 V s^{-1}.

Interpretation of the Results

As previously discussed, the single-electron reduction of Cp_2TiCl_2 with zinc first gives $[Cp_2TiCl_2]^-$.[35] The Lewis acid, $ZnCl_2$, then abstracts a chloride, resulting in the formation of $[Cp_2TiCl]$ and its dimer $[(Cp_2TiCl)_2]$. Furthermore, the cation Cp_2Ti^+ can usually be observed in the cyclic voltammogram. However, it was concluded that this species is formed during the CV measurement and is normally not present in Zn-Cp_2TiCl_2 solutions (father-son-relationship).[35]

In the presence of Coll•HCl, an adduct of the monomer and the hydrochloride is formed, namely $[CollH]^+$ $[Cp_2TiCl_2]^-$.[36] The relationship between this adduct, the monomer, and the dimer can be illustrated in form of the equations **I** and **II** shown in Scheme S1. Under the conditions of our measurements, only $[Cp_2TiCl_2]^-$ and the dimer $[(Cp_2TiCl)_2]$ are visible (Figure S2), which indicates that the monomer is majorly converted into the hydrochloride adduct (the oxidation wave of the monomer is greatly reduced). The presence of Coll•HCl further prevents the formation of $[Cp_2Ti]^+$. These observations are in agreement with earlier recorded cyclic voltammograms of Mn-reduced Cp_2TiCl_2 in presence of Coll•HCl.[36]

The addition of 10 equiv of $ZnCl_2$ to Zn-reduced Cp_2TiCl_2, however, results in a completely different voltammogram (Figure S3) that shows only the oxidation wave of Cp_2Ti^+. As discussed in the manuscript text, this has been interpreted as the formation of an adduct of Cp_2TiCl and $ZnCl_2$, namely $[Cp_2Ti]^+[ZnCl_3]^-$ [Scheme S1, Eq. (**III**)]. As is noted in the manuscript, the presence of $ZnCl_2$ prevents a measurement at $E < -1.2$ V, which makes it impossible to detect $[Cp_2TiCl_2]^-$. However, it is assumed that $[Cp_2TiCl_2]^-$ is not present under these conditions.

The experiment with both additives (Coll•HCl and $ZnCl_2$) then shows again a new situation (Figure S4). Only the oxidation wave of the monomer $[Cp_2TiCl]$ is observed, which possesses a free site for substrate coordination. The formation of the adduct $[Cp_2Ti]^+$ $[ZnCl_3]^-$ is suppressed since $ZnCl_2$ becomes unavailable for coordination to the titanium(III) center. Instead, Coll•HCl and $ZnCl_2$ now form an adduct, the formation of which releases $\Delta G = -8.5$ kcal mol^{-1} (see the chapter on the DFT calculations). As a result, $[Cp_2TiCl]$ is released again from any $[Cp_2Ti^+]$ which may have been formed [Eq. (**IV**)]. Interestingly, the dimer $[(Cp_2TiCl)_2]$ is not visible under these conditions and, hence, the presence of an excess of $ZnCl_2$ leads to a break-up of this dimer, even in presence of an equal amount of Coll•HCl.

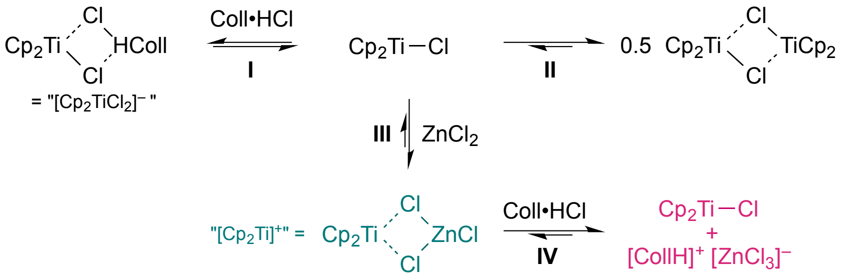

Scheme S1. Equilibria between the species observed in the cyclic voltammetry measurements.

Determination of the Order in Catalyst by Visual Kinetic Analysis

Two decyanation reactions of **1a** with 5 mol% and 12.5 mol% Cp_2TiCl_2 were set up following the general decyanation procedure on a 4 mmol scale using a 100 ml Schlenk tube equipped with a rubber septum. 1,3,5-Trimethoxybenzene (89.7 mg, 0.533 mmol, 0.133 eq) was added as internal standard.[37] During the reaction, a positive pressure of argon was maintained. Samples were taken from the reaction, filtered, and analyzed by NMR. The data obtained is shown in Figure S5.

The Visual Kinetic Analysis developed by Burés was applied to determine the order in catalyst.[38,39] First, the time scale was normalized assuming a first order in catalyst, but no overlay could be achieved (Figure S6). In contrast, an excellent overlay could be achieved for an assumed **second order** dependence on Cp_2TiCl_2 as is shown in Figure S7.

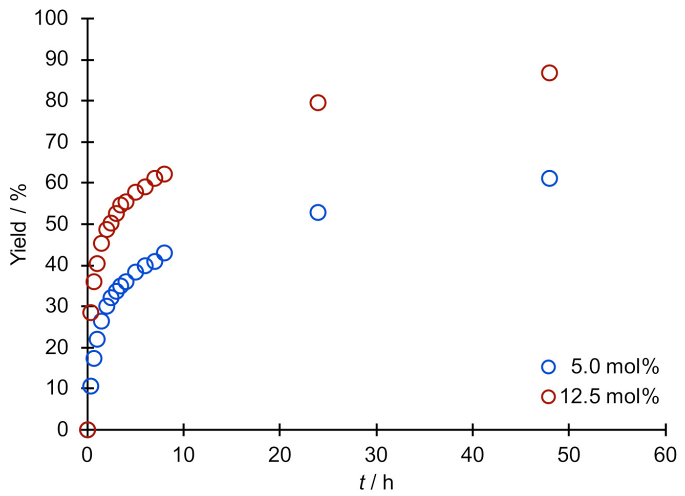

Figure S5. Data obtained without correction for the catalyst loading.

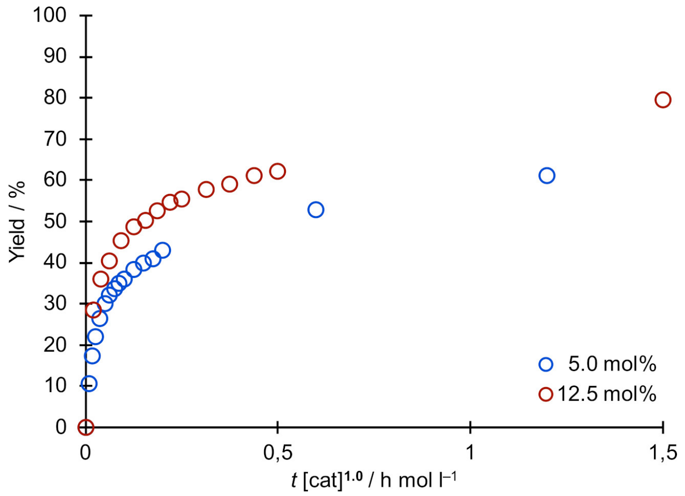

Figure S6. No overlay was achieved for an assumed first order in catalyst.

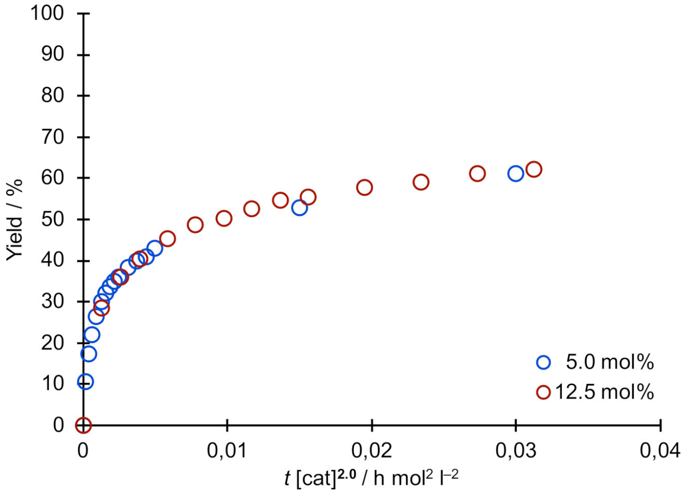

Figure S7. Excellent overlay is achieved for a second order in catalyst.

Qualitative Verification of Cyanide Formation

The decyanation of **1a** was carried out on a 4.0 mmol scale according to the general catalytic decyanation procedure. After cooling to room temperature, the mixture was transferred into a separation funnel, containing Et_2O (30 ml) and water (10 ml). The organic layer was extracted with water (3 × 10 ml). Traces of the organic solvents were removed from the aqueous layer by reduced pressure. A volume of 0.5 ml of the aqueous layer was dissolved in 1.0 ml and given to the IC. Reference samples of NaCl (1 mg in 1 ml H_2O) and NaCN (5 mg in 1 ml H_2O) were analyzed for comparison.
t (CN^-) = 2.03 min, t (Cl^-) = 3.19 min.

The chromatograms of the references (Figure S8 and Figure S9) and of the aqueous layer of the decyanation (Figure S10) are shown. The latter clearly shows the presence of cyanide at t = 2.03 min. The chloride peak appears several times bigger than the cyanide peak because of the lower electric conductivity of aq. NaCN and because of the presence of several equivalents of chloride under the reaction conditions (from TMSCl, $ZnCl_2$, Coll•HCl).

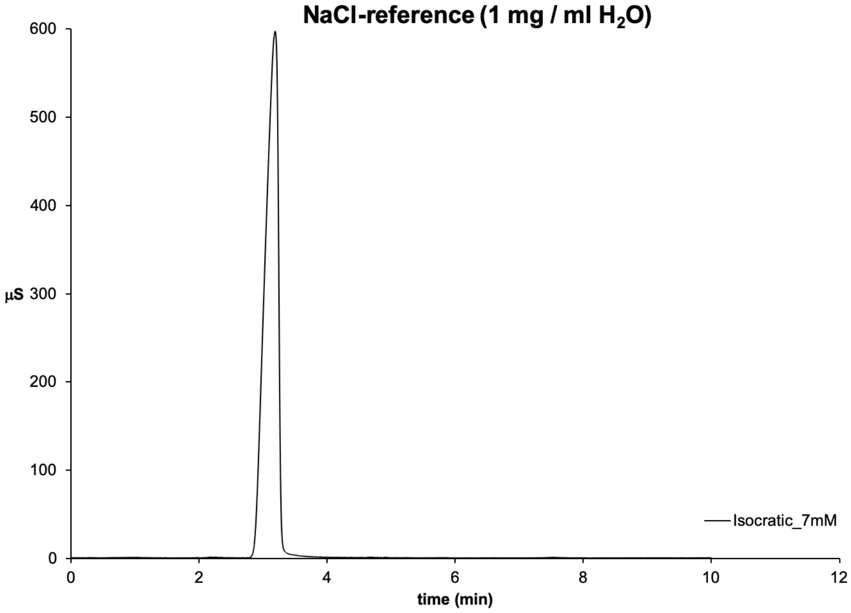

Figure S8. Ion chromatogram of the NaCl sample.

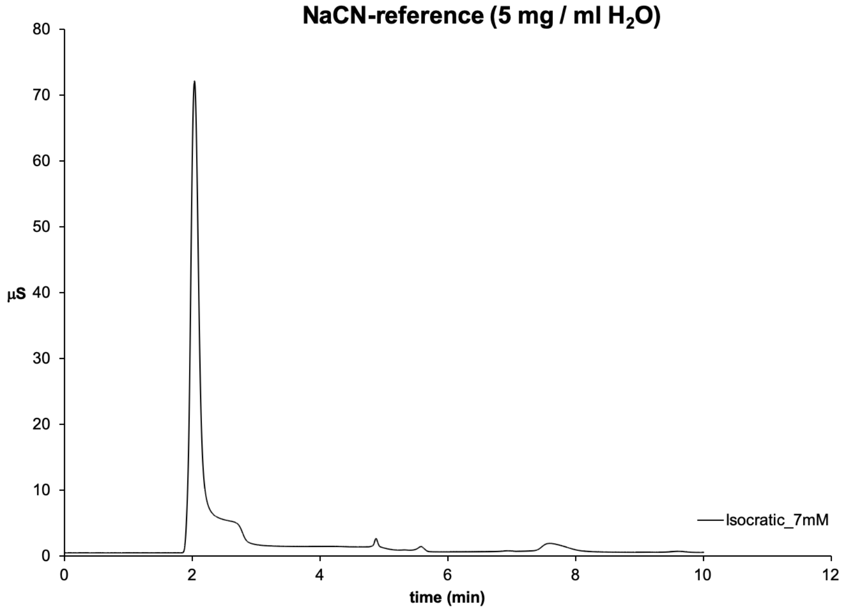

Figure S9. Ion chromatogram of the NaCN sample.

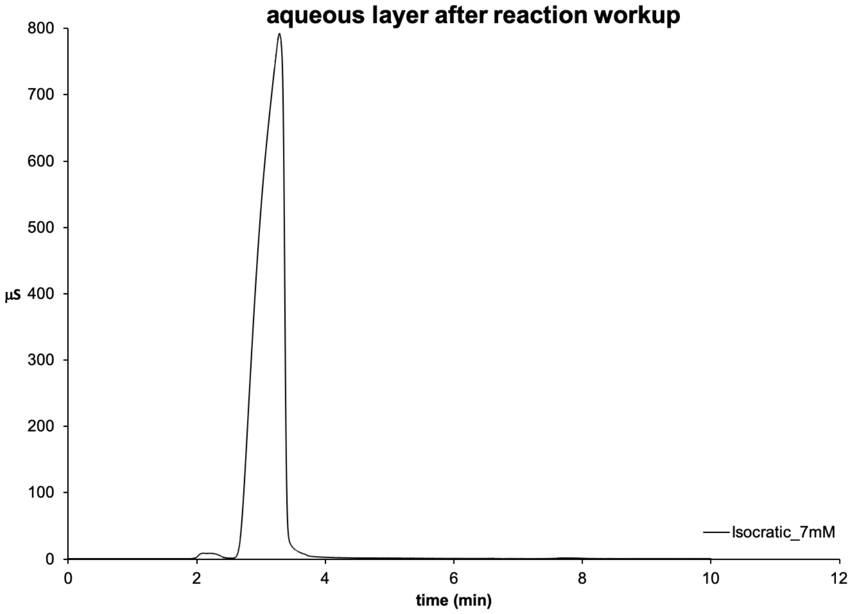

Figure S10. Ion chromatogram of the aqueous layer of the decyanation of **1a**, confirming the presence of CN^{-}.

Computational Details

The Orca 4.1.1 program package was used for the DFT calculations.[40] The RI-J approximation for Coulomb integrals and the COSX numerical integration for HF exchange (RIJCOSX) were applied.[41,42] Furthermore, the D3 dispersion correction with Becke-Johnson damping, D3(BJ), was applied in all calculations.[43,44] All structure optimizations were finalized using the TPSS functional[45] together with the def2-TZVP basis set[46] and matching auxiliary basis sets.[47] The conductor-like polarizable continuum model (CPCM)[48] was applied for the optimizations. The optimizations were carried out with the *Grid3 FinalGrid5* option. Frequency analyses were carried out numerically (*NumFreq*). Stationary points (minimum structures) were characterized by the absence of imaginary frequencies. The correction to the Gibbs Free Energy was obtained from the Orca-output of the frequency calculation. Single-point calculations were carried out using the PW6B95[49] functional and the def2-QZVP basis set and matching auxiliary basis sets.[50] The single point calculations were carried out with the *Grid4 FinalGrid5* option. The CPCM model together with the correction $\Delta G^{*\rightarrow o}{}_{solv}$ (= 1.90 kcal mol^{-1}, see Born-Haber cycle in Scheme S2) were applied to obtain the energy in solution. For the interconversion energy THF(g, 1bar)→THF(l), a quasi-experimental value of $-\Delta G^{o}{}_{vap}$ = –0.91 kcal mol^{-1} was used.[51]

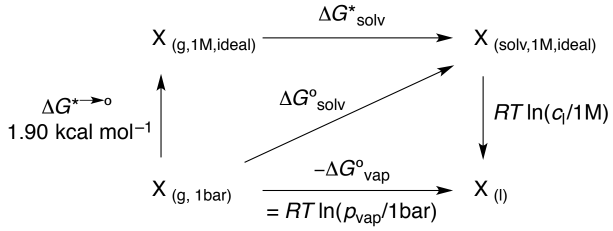

Scheme S2. Interconversion Scheme for the calculation of solvation energies for a compound X.

The Product Inhibition Scenario and the Formation of [CollH]$^{+}$ [ZnCl$_3$]$^{-}$

To support the conclusion that $ZnCl_2$ prevented the product inhibition of the catalyst, DFT calculations of the involved equilibria have been carried out (Scheme S3).[18] The calculated energies of the individual species are given in Table S1. The results show that the displacement of malononitrile **1a** in [Cp$_2$Ti(**1a**)Cl] with nitrile **2a** or **2b** is favored by about 1.0 kcal mol^{-1} (Scheme S3a and b). Hence, product coordination is slightly more favorable than substrate coordination, leading to a reduction of the catalytically active substrate-catalyst complex. However, the transfer of benzyl cyanide from [Cp$_2$Ti(**2b**)Cl] to zinc chloride is even more favorable (ΔG = –3.8 kcal mol^{-1}), resulting in the liberation of the active catalyst (Scheme S3c). The transfer of **1a** from [Cp$_2$Ti(**1a**)Cl] to $ZnCl_2$ is also found to be slightly exergonic (ΔG = –2.0 kcal mol^{-1}, Scheme S3d). Overall, the added $ZnCl_2$ gets preferably coordinated by **2b**, even in presence of **1a.** The catalyst is freed and the product inhibition reversed. This is shown in Scheme S4, which puts these equilibria into relation.

The energy of formation of [CollH]$^{+}$ [ZnCl$_3$(THF)]$^{-}$ from Coll•HCl and $ZnCl_2$•2THF was calculated as shown in Scheme S5.

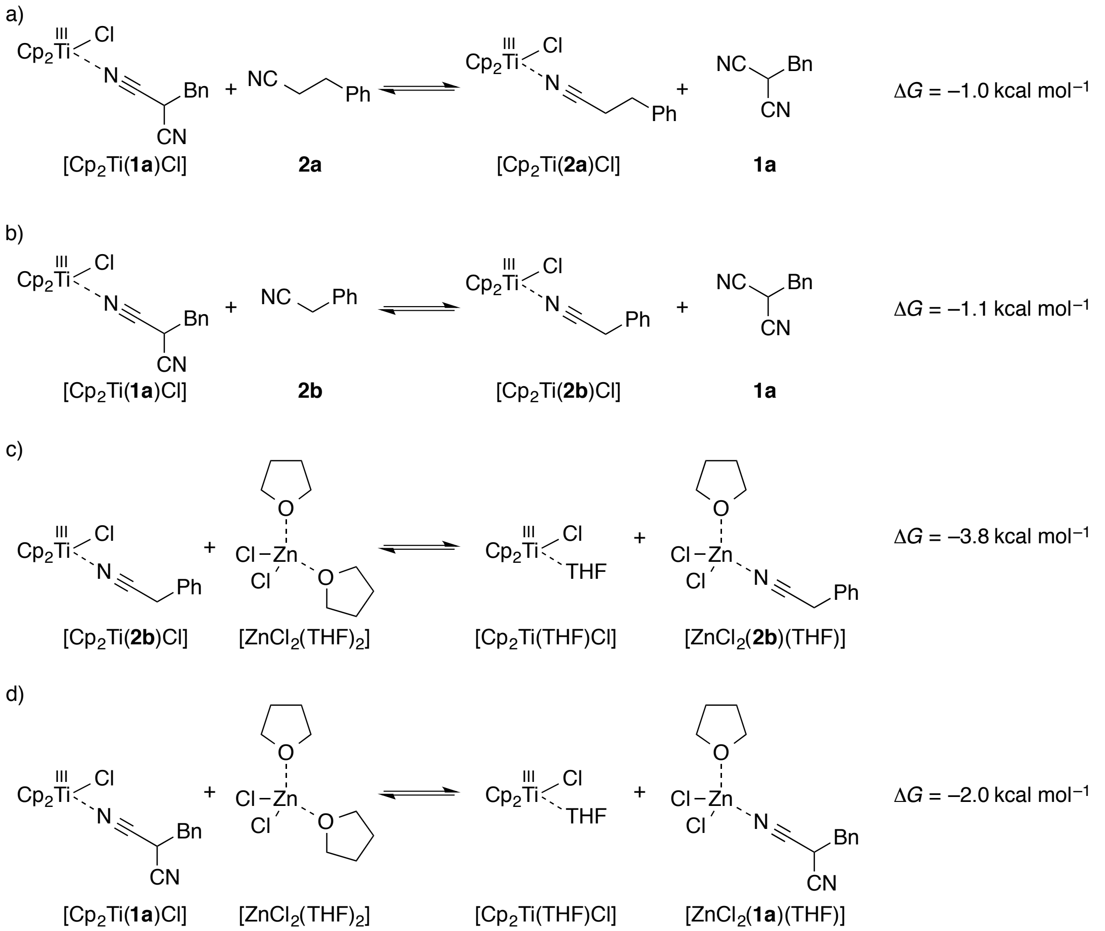

Scheme S3. Calculated competitive equilibria for the coordination of the substrate and product to the titanium catalyst and zinc chloride.

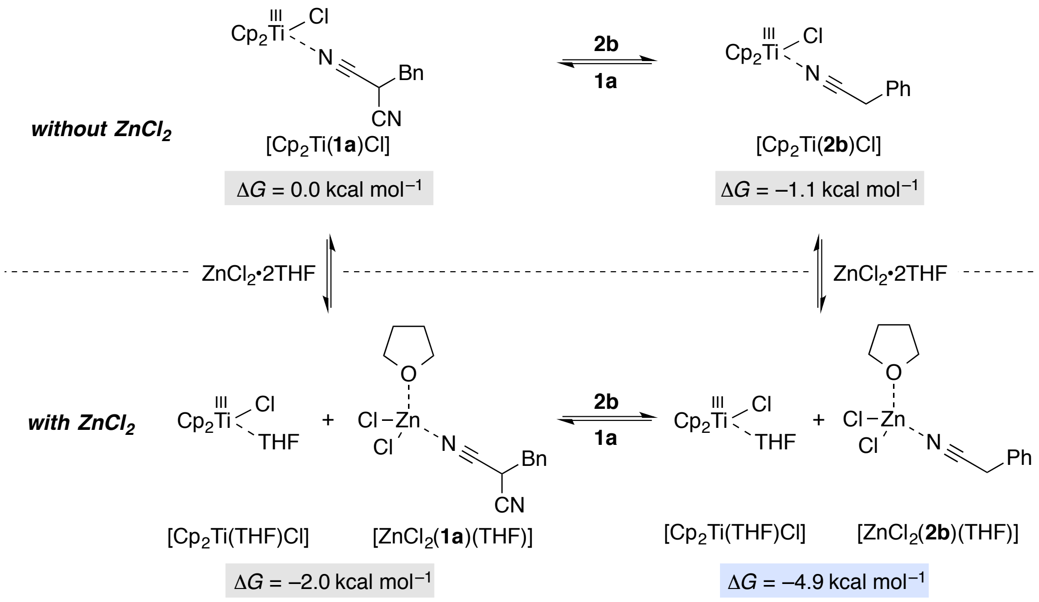

Scheme S4. Connection between the equilibria.

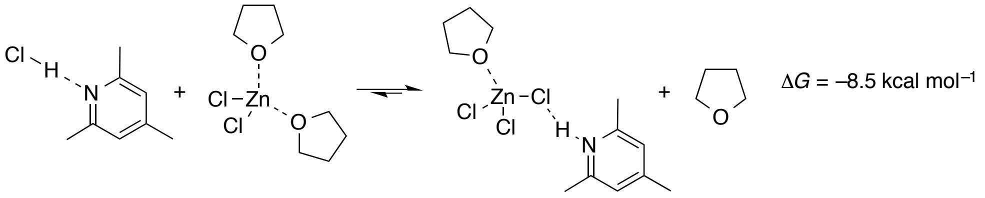

Scheme S5. Calculated formation of $[CollH]^+[ZnCl_3(THF)]^-$ from Coll•HCl and $ZnCl_2$.

Table S1. Energies of the calculated species.

**Compound**	**TPSS-CPCM/def2-TZVP / Hartree**	**PW6B95-CPCM/def2-QZVP/ Hartree**	**Correction to *G* / kcal mol^{-1}**	G_{solv} **/ kcal mol^{-1}**
Cp_2TiCl(**1a**)	–2192.8958286	–2194.7602561	170.2	–1377060.75
Cp_2TiCl(**2a**)	–2100.6126611	–2102.3742073	172.06	–1319085.77
Cp_2TiCl(**2b**)	–2061.2701908	–2062.9874942	154.89	–1294387.41
Cp_2TiCl(THF)	–1929.8300691	–1931.3874787	150.92	–1211811.12
$ZnCl_2(THF)_2$	–3165.2438251	–3167.3851636	121.77	–1987440.53
$ZnCl_2$(**1a**)(THF)	-3428.299653	-3430.75615	137.89	-2152692.20
$ZnCl_2$(**2b**)(THF)	–3296.6790449	–3298.9880572	123.75	–2070020.61
$[CollH]^+$ $[ZnCl_3(THF)]^-$	–3760.016323	–3762.668599	156.37	–2360951.92
**1a**	–495.6532988	–496.2214022	72.94	–311308.79
**2a**	–403.3702966	–403.8338467	74.88	–253332.78
**2b**	–364.0293583	–364.4486505	58.7	–228634.38
Coll•HCl	–827.3523286	–828.1169007	89.94	–519559.36
THF	–232.5927471	-232.841937 (gas phase)	55.00	–146056.43*

* A quasi-experimental value for $-\Delta G^o_{vap} = -0.91$ kcal mol^{-1} was used for THF.

Coordinates

Cp_2TiCl(**1a**)

C	-3.35039926987281	0.71046750098822	2.57358290634617
C	-4.32041489803206	1.01474479944583	1.59280309505214
C	-3.65525219276194	1.14661119057230	0.33789956942075
C	-2.08507535741409	0.63329482088970	1.92909679539787
C	-2.27763861723667	0.92225311171252	0.54717861240488
H	-1.51010958829151	0.93440909909129	-0.21478292848615
H	-4.12439478649824	1.36152952859120	-0.61219627698613
H	-3.54183721597510	0.51276785551821	3.61833594000136
C	-5.60141191460186	-1.96840874067594	0.98430085990906
C	-5.36364655375159	-1.21527806098820	-0.20214661555655
C	-4.34877386894811	-1.86761463804336	-0.94881218700198
C	-3.98794729463045	-3.04928830868388	-0.23569672054354
C	-4.76326473804425	-3.11033125711793	0.94252284145551
H	-6.30169214037492	-1.71840024185311	1.77025557574300
H	-5.86400623581091	-0.29824319558509	-0.47689473980232
H	-3.22723273790853	-3.75952195776428	-0.52854285576010
Ti	-3.38786718755093	-1.11690169235100	1.05817111056991
H	-1.14790060313592	0.37009939532835	2.39794521384558
H	-4.67978984469413	-3.86059384576413	1.71436982684217
Cl	-2.72994929783255	-2.32583237785260	3.12080625276731
N	-1.52874496740360	-1.79489865947994	0.33413357176409
C	-0.47615491331344	-2.13021283244490	-0.01719433986937
C	0.86449875171051	-2.55464674229266	-0.41702608596664
C	1.52106314919221	-1.47373772066153	-1.16765095031269
N	2.05792127006576	-0.62624457675760	-1.74711092450657
C	1.71961131214679	-3.00640433513357	0.82445934335873
H	0.75385463004354	-3.41267192928062	-1.09376571353988
H	-3.93205751506093	-1.53645888515770	-1.89074818796112
H	-5.38531822230490	1.10894258685800	1.75850973064822
H	4.13652298957805	0.57420279483979	2.61078861062956

C	3.24667945703576	-0.04603139056119	2.67011738549460
C	2.28537041319675	0.19823196868508	3.65307436346450
H	2.42427241974520	1.01035884335263	4.36104081762492
C	1.14798222368357	-0.60861605598348	3.72577536941236
H	0.39688473173288	-0.42840276184971	4.48972699373237
C	0.96966160665994	-1.65351622541651	2.81881866506429
H	0.07264409232837	-2.26537932579735	2.87963257309149
C	1.92863253662804	-1.90229790857484	1.82820327042969
C	3.06847484508742	-1.09097918895721	1.76297939226117
H	3.82066993397212	-1.28176474296289	1.00169557927626
H	2.67078124491737	-3.36771612691323	0.42538188927158
H	1.18452915372512	-3.85065677096868	1.26598847101349

Cp_2TiCl(**2a**)

C	-3.36925736647408	0.66128950903401	2.61398413854584
C	-4.32388589548777	0.99675454460025	1.62850706647084
C	-3.64028192443040	1.16496941196867	0.38829533958258
C	-2.09431968918767	0.60188008710259	1.98578619494661
C	-2.26630298912279	0.93201288612677	0.61093007158638
H	-1.48793284055343	0.96002976057085	-0.13938494629321
H	-4.09622107790348	1.40695301923037	-0.56196313914223
H	-3.57652065568662	0.43268115658766	3.64948560249044
C	-5.59660978465221	-1.97242969581137	0.94274541242680
C	-5.35696411770093	-1.18058910050657	-0.21747212014357
C	-4.34123705247371	-1.80847877354201	-0.98479886274724
C	-3.98279869125798	-3.01330199841497	-0.31157022295130
C	-4.75849530079127	-3.11278533459324	0.86329851559430
H	-6.29849747045197	-1.74924459485032	1.73542534429679
H	-5.85660471507689	-0.25467552988414	-0.46259939787229
H	-3.21939068894092	-3.71151594077213	-0.62537820462395
Ti	-3.38218723769139	-1.12261499211387	1.04807987760745
H	-1.16497623381275	0.31908809740233	2.45862103492827
H	-4.67443446266782	-3.88756169389798	1.61059777901163
Cl	-2.73663986767709	-2.39462017264584	3.08828757554861
N	-1.50626696674664	-1.80168989137229	0.33029736821672
C	-0.45719820835327	-2.16020828618653	-0.00773604366080
C	0.87172979433370	-2.62988291898908	-0.36250382946368
C	1.68648075643962	-3.05612468583732	0.89289519832890
H	-3.92412891571506	-1.44670818315263	-1.91533527801182
H	-5.39121504268504	1.08649714136086	1.78102804828287
H	4.09344657538686	0.64127833163768	2.45551196345827
C	3.22000371749577	0.00557571449664	2.57302296644249
C	2.29440565582998	0.26652737375184	3.58623313277340
H	2.44397291560244	1.10647920281250	4.25889753107067
C	1.17823263290192	-0.56013488755963	3.73125368591563
H	0.45302926034723	-0.36548573646184	4.51674521160471
C	0.98709690672123	-1.64107697152514	2.86898068383555
H	0.10350120959786	-2.26532484377142	2.98118215805248
C	1.91106867740504	-1.91101873086546	1.85122478874881
C	3.02779950933957	-1.07624224665355	1.71263549859895
H	3.75321115813890	-1.27838290844580	0.92740981543398
H	2.64260073943229	-3.44212726107069	0.52704856561200
H	1.15783697357883	-3.87411891546259	1.39017121923874
H	1.39025402473872	-1.82633553181940	-0.89769010175421
H	0.76129218825119	-3.47609341047719	-1.05013394198641

Cp$_2$TiCl(**2b**)

C	-2.24775829170473	-2.92006882114570	0.86340825518386
C	-3.49700356969027	-2.26068361864643	0.77101954614843
C	-3.67930830650654	-1.86822208509504	-0.58762337742254
C	-2.52597674105388	-2.24770377768626	-1.32006588057615
H	-4.19166080828485	-2.09181292489298	1.58342054916231
C	-1.64200681260690	-2.90551886431957	-0.41254503138050
H	-1.80039331727166	-3.30104850358201	1.76932592855429
H	-0.66223944264429	-3.29549538932645	-0.65134728281606
Ti	-1.86881368718222	-0.61261328881614	0.22682059634974
C	-2.57800299408880	1.35795077817495	1.32877212808500
C	-1.45639005130896	1.68368367261176	0.51439907023540
C	-3.63495617290932	0.97075595560266	0.47423088643687
C	-3.17603944266783	1.06683886248317	-0.87213929796301
C	-1.83908950468115	1.51895866549229	-0.84616010861377
H	-4.61468201221074	0.63497202868478	0.78688327226292
H	-2.59915358765708	1.35874958572677	2.40921117285234
H	-0.47679883980303	1.97128207347516	0.86924380687946
H	-3.74415913586828	0.81858662904529	-1.75812701440042
H	-1.20432015506352	1.67010859840555	-1.70850345189607
Cl	-0.46782729588915	-0.82037008051001	2.27882566461051
N	0.02767114852378	-0.62051450319731	-0.71887411074235
H	-4.54408234349467	-1.35701706015425	-0.98535641862804
H	-2.35119460192237	-2.07838390023729	-2.37433673492454
C	1.11586185108924	-0.55530667185425	-1.11067098194538
C	2.51322275334165	-0.43145435373249	-1.49021682068985
C	3.28289740615602	0.48249277444605	-0.53924507075982
H	2.57089540621333	-0.05382438997742	-2.51719630798476
H	2.94829744274009	-1.44011051860141	-1.49373657534208
C	4.43592894853431	1.12096308678355	-1.00720992998264
C	2.87587375928662	0.67067710395730	0.78483210190148
C	3.62267685481097	1.48869519223214	1.63498310301549
C	4.77670442602188	2.12096996531784	1.16946884710023
C	5.18180111385764	1.93502393831027	-0.15427736208014
H	4.74889745908973	0.98286527319229	-2.03923596838799
H	6.07540590405387	2.42796613544514	-0.52670446962412
H	3.29760770876142	1.63185828912263	2.66163266990690
H	5.35493036573266	2.75897078858311	1.83169269489402
H	1.97318476629702	0.18777905468230	1.15542220258098

Cp$_2$TiCl(THF)

C	-0.74230796312454	2.37486116495638	-0.43482449980729
C	-2.07928599669570	2.05400889508321	-0.81289587151924
C	-2.68231081349556	1.38464829044605	0.27715767021600
C	-1.72191008995373	1.27952124862235	1.32588412790905
H	-2.53906609658574	2.26581790394065	-1.76812368973716
C	-0.53690547790024	1.91216708808618	0.88814912272292
H	-0.00748211807244	2.85855543427705	-1.06199787841282
H	0.38602549945403	1.98389087260889	1.44806900138309
Ti	-0.93332815083104	0.01196954590227	-0.55603534241112
C	-1.51710605867646	-2.04854719503134	-1.70286211294095
C	-0.60004123369625	-2.36383408811581	-0.67615721751628
C	-2.68524986951693	-1.51109234949605	-1.10947955827905
C	-2.49686357101582	-1.52903824221351	0.30238539650599
C	-1.20139869570536	-2.03985157880313	0.57454457700666
H	-3.56190764370363	-1.15400623258689	-1.63382537107964
H	-1.32930901042728	-2.13133334249489	-2.76287762359781

H	0.40491422189932	-2.73685094789329	-0.82169368422787
H	-3.21349191966377	-1.20476595978796	1.04295197072313
H	-0.76959022560954	-2.18722842852913	1.55485680420305
H	-3.69258338703538	1.00130687581482	0.30201117844576
H	-1.87146342883111	0.79459485259235	2.28155370067608
C	3.55406775027666	0.18976381606549	0.04280048856574
C	2.31532660644981	0.58246921952731	-0.74588477825537
O	1.21855795556458	-0.15687681820899	-0.10346815665036
C	1.65145538364380	-0.62958958139792	1.21796124401986
C	2.99754203520664	0.03901273259302	1.46574938279720
H	4.33745793463001	0.94802404117848	-0.03143662992774
H	3.95073400815249	-0.76483693247372	-0.31829823423306
H	2.31971195736737	0.29210466545629	-1.79454076538806
H	2.09520223990286	1.65190303819533	-0.66205815649028
H	1.73370127183257	-1.71954447834473	1.16292642362348
H	0.87796077917013	-0.35521681595602	1.93786403514100
H	3.63408818531808	-0.56383869812006	2.11783113906289
H	2.85707108769920	1.02320704092627	1.92465894188569
Cl	-0.15221486602703	0.44862486318109	-2.87089583441347

$ZnCl_2(THF)_2$

Zn	-0.00166580600147	0.00495544410652	1.07479064766991
Cl	0.47682368531533	1.93515159146707	2.05033403242685
Cl	-0.48055992403194	-1.92389914874286	2.05304786999194
O	-1.44847667631557	0.41812589801201	-0.34272778261101
O	1.44599200299728	-0.41016349357781	-0.34097046960981
C	2.55833436974692	-1.27513630215316	0.06933080676399
C	3.59480560782612	-0.29603646815625	0.60019585945248
H	2.90422741261937	-1.80993225476857	-0.82197305169586
H	2.15774406206716	-1.97713545690938	0.80083558582696
C	3.44691351913978	0.92932777299496	-0.33697242857023
H	4.60273581109878	-0.71707180119505	0.58068226565471
H	3.35067450431892	-0.02239865949847	1.63054686943827
C	2.06428154149840	0.75680963739040	-0.99419019675659
H	4.22759305895371	0.93697457921514	-1.10130388639683
H	3.50415251911527	1.86331268496258	0.22503688222825
H	2.13422781385628	0.52427525514211	-2.06019043941383
H	1.38661961426506	1.59608790427689	-0.83752638237656
C	-2.56594103471782	1.27563010568885	0.06922339658726
C	-3.59552575493692	0.28957901592823	0.60100319809453
H	-2.91650313752963	1.80819850110770	-0.82157358687748
H	-2.16912845733531	1.98018980198353	0.80033445741227
C	-3.43935824505058	-0.93575404944266	-0.33512404349863
H	-4.60634566304413	0.70360677821145	0.58135531290445
H	-3.34929943594890	0.01842977290824	1.63148046960489
C	-2.06089203352494	-0.75087439010061	-0.99761438318606
H	-4.22280306219053	-0.95239997363986	-1.09645260608480
H	-3.48514375530414	-1.86926920382333	0.22869439874536
H	-2.13715657870873	-0.51489381725442	-2.06244200560549
H	-1.37632595817776	-1.58568972413322	-0.84718379011896

$ZnCl_2$(**1a**)(THF)

C	-2.24965225788680	-0.50876328880039	0.93211742171245
C	-1.40590168777670	-1.66112856920052	1.42563888160929
C	-2.79700114828938	-0.55503770887678	-0.35502990549625
C	0.67912896690891	-0.37417541095739	0.93728426377908
N	1.17357601478956	0.29309715743606	0.13922135949745

Zn	2.09435285749367	1.68933311457723	-1.08928200916018
O	0.51217629243164	2.00896861634405	-2.33711824372605
Cl	3.66795068903932	0.65910798521552	-2.25843480606649
Cl	2.29746947072955	3.51911135834469	0.15010376406439
C	-0.72382414209613	2.67208512784983	-1.89226784861362
C	0.25925535059517	1.21798614357543	-3.55639205957036
C	-1.25197875887393	1.26721051819211	-3.74185995292307
H	0.80144754228008	1.70623369241486	-4.37027634845445
H	0.66044780997753	0.21699157869292	-3.39056824358725
C	-1.63082430971157	2.61712744265452	-3.11062762714897
H	-1.13137458078776	2.10466983518239	-1.04937210128402
H	-0.44499428810737	3.67559925087088	-1.56934796272510
H	-1.40073980669657	3.44006959142265	-3.79471456302631
H	-2.68543526968636	2.67402588254265	-2.83230866279900
H	-1.72856499971604	0.44631400817995	-3.19728700361723
H	-1.53054147090073	1.19752652134730	-4.79594012861379
C	-0.00286703226986	-1.19003592395177	1.94362358002189
H	-1.25205687049770	-2.39144335797009	0.62902031045719
H	-1.87655626094341	-2.16909635014617	2.27293768015482
C	-2.51568280818429	0.60162528000027	1.74450335188604
C	-3.61389484479960	0.47956649913935	-0.81576541072329
H	-2.58915121970197	-1.40968471084472	-0.99367591022132
C	-3.87149145780876	1.58317805465837	-0.00208518766912
C	-3.31538985867424	1.64436154290437	1.27776568719279
H	-2.10491139084231	0.65512499279409	2.75006719844884
H	-3.51013499176921	2.50116073539292	1.91588922914339
H	-4.04236358064638	0.42443031660578	-1.81224161908478
H	-4.49887536935292	2.39299800894404	-0.36250597407965
C	0.83398307564009	-2.33031763108373	2.33718683319584
H	-0.13416844248544	-0.55128823778368	2.82720608858282
N	1.45145917861988	-3.25637916566702	2.65621221884409

$ZnCl_2$(**2b**)(THF)

C	-2.25666445599233	0.52147925576011	1.51228766378138
C	-1.47806523104287	-0.49400456182601	0.95212224468073
C	-1.93391396153552	-1.17900342665706	-0.17876307732459
C	-3.16635488736231	-0.85138111310852	-0.74285384577820
C	-3.94644902465201	0.16182159042630	-0.18095844167461
H	-3.51602368918082	-1.38646368874994	-1.62073913192278
H	-1.32726797590083	-1.96748431381700	-0.61657525292929
C	-3.49168451797689	0.84553013736037	0.94760354273006
H	-1.89886441501034	1.05794494462349	2.38696472994071
H	-4.09415357599138	1.63445667891342	1.38795773140690
H	-4.90471358633774	0.41830559290309	-0.62285364681670
C	0.93796559339623	-0.26403858513823	0.73263177812894
N	1.72766036314123	0.20935944837329	0.03559052550739
Zn	2.72954183761378	1.19685190308141	-1.46327698531878
O	0.98150059825310	1.78791556638971	-2.39108920979759
Cl	3.67306612800090	-0.28302429163519	-2.81796905044687
Cl	3.65091272074842	3.03147658269259	-0.62416192214419
C	0.14224342005064	2.84534942685388	-1.79211319317042
C	0.12800159536869	0.78526514297055	-3.05177488271524
C	-1.22403659801633	1.46810304011566	-3.18522418313523
H	0.61235359284861	0.53206506689291	-3.99518728166311
H	0.08026553755131	-0.09972607729397	-2.40866270779237
C	-1.28395426004753	2.32751725448247	-1.91278825115474
H	0.48251321824734	2.99675529424203	-0.76625833870563

H	0.31325472746379	3.75376591446354	-2.37574287161310
H	-2.00604709850924	3.14419192673415	-1.98382146313418
H	-1.54267847219560	1.70772570965814	-1.04950240134171
H	-2.03856377033809	0.74261545206570	-3.24310370360492
H	-1.24653959532779	2.09903681302112	-4.07930125143845
C	-0.11884273220555	-0.83506588672454	1.55548935486141
H	-0.01776836903164	-0.43010795110661	2.56736317276509
H	0.04146398397083	-1.91784944596687	1.60459804982006

[CollH]$^{+}$ [ZnCl$_3$(THF)]$^{-}$

C	-0.02902466836798	1.77963492406978	-0.56791560678012
C	-1.04387036667552	1.84970183703711	0.37352495035199
C	0.44130490284070	0.54081149652890	-1.02163924818425
C	-0.14506770734316	-0.62260761462813	-0.50618936896519
C	-1.15965391945439	-0.54538337574442	0.43368136644865
N	-1.55961103256436	0.68526533356216	0.83844890991222
H	0.39265814284270	2.70297800613245	-0.94973455178983
H	0.18433763462552	-1.60063240004158	-0.83982851138326
C	-1.62243621466925	3.12338674234960	0.89993521916384
H	-1.04311627974517	3.97370486331560	0.53862968401805
H	-1.62410545233809	3.12312816881937	1.99428697252632
H	-2.66250883411044	3.23750187983323	0.56994876903112
C	1.56149013532849	0.46047245151035	-2.01764583826305
H	1.58425110517500	1.34554213139774	-2.65797431254698
H	1.47563203228923	-0.43733428549447	-2.63489622950064
H	2.51849948961821	0.40814828087712	-1.48310052435752
C	-1.86576375800405	-1.72452345695206	1.02026468942301
H	-1.40476946014327	-2.64974816946861	0.67294310530155
H	-2.91835961064120	-1.70627584346923	0.71559984170440
H	-1.83189888797149	-1.68982991609617	2.11379224634327
Cl	-4.06804862545934	0.87563652893886	2.76932405600323
Zn	-5.23063533830449	1.37405062327717	0.84148622729587
H	-2.33861150216523	0.73728590509701	1.52581553925339
Cl	-5.51605115967706	3.57488055009224	0.51572426872733
Cl	-4.54087605051806	0.11778284044631	-0.89098722116808
O	-7.09490347238068	0.63178850202050	1.36201444448162
C	-7.83663682308325	1.37557100607882	2.39823620301343
C	-7.17359095716666	-0.81648830071109	1.64106216091622
C	-7.77611429749827	-0.91726940351806	3.03496179376331
H	-6.16367727414681	-1.22200420739184	1.55824619524043
H	-7.81612115351066	-1.25315955192274	0.87127015510187
C	-8.68455725962731	0.32142934531835	3.09336970063497
H	-6.98965724761268	-0.84678545416587	3.79306397152148
H	-8.32055635883351	-1.85406233262253	3.17543494437669
H	-8.93655673913087	0.61661708433151	4.11482758157697
H	-9.61116001663996	0.14559522493684	2.53735796404435
H	-8.40117652959973	2.15361973824426	1.88320619888589
H	-7.10644844533692	1.82854564801152	3.07651415387747

1a

N	-2.35410456662087	-0.84793242090975	-2.68257947372407
C	-1.40686457125588	-0.92923956767590	-2.02109255679595
C	-0.22549975703152	-1.04983457731397	-1.15521339177188
C	0.79521107745728	-0.07004294478445	-1.55171078034763
N	1.60858293565149	0.71068505618290	-1.81811224892997
C	-0.59889667173925	-0.88545888560622	0.36196788758445
H	0.19616091219653	-2.05143337906280	-1.31043073484093

H	-4.90146169566387	-2.49087516820255	1.25213912801798
C	-3.83550481258085	-2.69900555082552	1.23295443847347
C	-3.36021774914488	-3.95713912683237	1.60695450609041
H	-4.05502232745137	-4.73153858640379	1.91923053050366
C	-1.98731228184278	-4.21510826249016	1.58456296032060
H	-1.61084006310014	-5.18983078412592	1.88193063650113
C	-1.09588547855446	-3.21877717956049	1.18730539604300
H	-0.02719951826474	-3.42092848692911	1.17704073833681
C	-1.56496548189478	-1.95371530500480	0.81018102036213
C	-2.94175872780251	-1.70292749103984	0.83652628029832
H	-3.31463409097428	-0.72305726252860	0.54938589741184
H	-1.01794984486738	0.11317380733046	0.50338462458995
H	0.34198151348425	-0.95054248421714	0.91438344187670

2a

N	-1.51500284364391	-1.74830198835056	0.17755504913211
C	-0.44944871826438	-2.13403941762571	-0.07198281896718
C	0.89242565892121	-2.63073894320372	-0.36515916678761
C	1.66046988629392	-3.06320282533158	0.91417334586256
H	4.05516336997351	0.67304278299088	2.40259563852423
C	3.19671634243613	0.02279060673763	2.54749605124078
C	2.29993603406377	0.26880031857432	3.59005402050775
H	2.45623203436962	1.11195630448748	4.25704158095812
C	1.20506676196710	-0.57772126509346	3.77346263137852
H	0.50493944022142	-0.39575914386332	4.58416262594315
C	1.00771828882963	-1.66247550382053	2.91732040587624
H	0.15495430078837	-2.32059741956967	3.06486485680189
C	1.89808604067578	-1.91589019599103	1.86649453702773
C	2.99510663060526	-1.06138478544969	1.69285452595080
H	3.69821682732369	-1.25028963021767	0.88441136841171
H	2.61417911342965	-3.48153299394480	0.57803909644282
H	1.09888587928919	-3.86027123240578	1.40964387117980
H	1.44132440893163	-1.83896991234249	-0.88659502146416
H	0.79751224378841	-3.47977645558029	-1.05099159801925

2b

C	-2.04334743608611	0.50513546540829	0.00078520016463
C	-0.78999815764784	1.11516009554286	0.00032531844206
C	0.37536673556404	0.33809657371080	-0.00053598334338
C	0.26909569927486	-1.05378207809423	-0.00095043306095
C	-0.98769742608391	-1.66489479315821	-0.00055195245163
C	-2.14612448983516	-0.88871554267474	0.00033824637121
H	-3.12272944377358	-1.36398743573404	0.00057051482041
H	-2.94012613830796	1.11834190558476	0.00122084740128
H	-0.71516363309646	2.19992292630726	0.00074182212574
H	1.16616305480244	-1.66704717362610	-0.00143888712956
H	-1.05633924723277	-2.74898698883478	-0.00089829648247
C	1.71903264196716	1.05865089786251	-0.00075575967446
C	2.87727727270056	0.17017587873182	0.00129132731714
N	3.79717770354433	-0.53637003255604	0.00291684533357
H	1.79805037176358	1.71023248889309	0.87838360651181
H	1.79936249244683	1.70806781263675	-0.88144241634541

Coll•HCl

Cl	0.02058444169404	-0.00024121639229	3.34850176718438
C	-0.31223617264328	-1.15127277507656	-0.31219788459850
C	0.33019755994621	1.15038832291748	-0.31165984393048

C	-0.31700029690586	-1.15731390639875	-1.69849768665345
C	0.33003225898406	1.15717266721894	-1.69941688653774
H	-0.57028764252708	-2.07610627016339	-2.21681638311887
H	0.59021966885450	2.07351721149570	-2.21833751780397
N	0.00946519852820	0.00008718894626	0.32630933892773
C	0.00303068659462	0.00156208427747	-2.41816776382681
H	0.01397625159380	-0.00112667482445	1.39569646610989
C	0.66273549805330	2.34053702909348	0.53242388309029
H	0.94703507751922	3.18112587269005	-0.10181040029047
H	-0.20016843078921	2.62422226096413	1.14396967103743
H	1.48233057509358	2.10537332628065	1.21847127205676
C	-0.63724297790501	-2.34252758942896	0.53344506848707
H	-1.47779961010441	-2.11988722403814	1.19832230239059
H	-0.89073463675816	-3.19330278284114	-0.10030567448283
H	0.21657285329956	-2.60314955411516	1.16723124163555
C	-0.02441549923364	0.00488882159943	-3.91965018173997
H	0.37744472303295	-0.93069215962128	-4.31891114830642
H	-1.06131040931404	0.09184905795564	-4.26762726309251
H	0.54186098298665	0.84658640946088	-4.32452757653768

THF

C	-0.01977997374256	0.79723037697286	0.77748716338541
C	-0.01204527341353	-0.69824935825269	1.14060268392670
O	-0.58065442449003	-1.37177206506077	-0.00002911701209
C	-0.01196753022978	-0.69823597609796	-1.14061812656761
C	-0.01990758153144	0.79724323903561	-0.77749985817668
H	0.84945748087374	1.31364757898040	1.19358820210381
H	-0.91970595370894	1.28340365113955	1.16314564210566
H	-0.62346736211580	-0.95222849401666	2.00925762818540
H	1.01595940224299	-1.05795807757222	1.30200194956889
H	-0.62320756785117	-0.95238162143900	-2.00935811689667
H	1.01612066388600	-1.05778216899957	-1.30186444594263
H	-0.91998656635287	1.28328907597547	-1.16297244443757
H	0.84918498643339	1.31379383933497	-1.19374116024261

References

1 C. P. Grugel, B. Breit, *Chem. Eur. J.* **2018**, *24*, 15223–15226.
2 J. Li, M. J. Lear, Y. Hayashi, *Angew. Chem. Int. Ed.* **2016**, *55*, 9060–9064.
3 T. Kawamoto, Y. Shimaya, D. P. Curran, A. Kamimura, *Chem. Lett.* **2018**, *47*, 573–575.
4 K. Okuro, M. Furuune, M. Miura, M. Nomura, *J. Org. Chem.*, **1993**, *58*, 7606–7607.
5 Y. Jiang, N. Wu, H. Wu, M. He, *Synlett* **2005**, 2731–2734.
6 D. E. Q. Jimenez, I. M. Ferreira, W. G. Birolli, L. P. Fonseca, A. L. M. Porto, *Tetrahedron* **2016**, *72*, 7317–7322.
7 L. W. C. Lawrence, S. A. Styler, A. P. Dicks, *J. Chem. Educ.* **2010**, *87*, 628–630.
8 Y.-Q. Yang, Z. Lu, *Chin. J. Chem.* **2014**, *32*, 650–653.
9 S. J. Garden, C. R. Werneck Guimares, M. B. Correa, C. A. Fernandes de Oliveira, A. da Cunha Pinto, R. B. de Alencastro, *J. Org. Chem.* **2003**, 68, 8815–8822.
10 H. J. Cristau, R. Vogel, M. Taillefer, A. Gadras, *Tetrahedron Lett.* **2000**, *41*, 8457–8460.
11 J. P. Peterson, M. R. Geraskina, R. Zhang, A. H. Winter, *J. Org. Chem.* **2017**, *82*, 6497–6501.
12 C. Gao, X. Tao, Y. Qian, J. Huang, *Synlett* **2003**, 1716–1718.
13 J. Han, X. Qian, B. Xu, L. Wang, *Synlett* **2017**, *28*, 2139–2142.
14 Z.-L. Wu, Z.Y. Li, *Tetrahedron: Asymmetry* **2003**, *14*, 2133–2142.
15 H. K. Oh, S. J. Song, D.-S. Jo, I. Lee, *J. Phys. Org. Chem.* **1997**, *10*, 91–96.

16 D. V. Schoonover, H. W. Gibson, *Tetrahedron Lett.* **2017**, *58*, 242–244.
17 N. S. Dange, B.-C. Hong, C.-C. Lee, G.-H. Lee, *Org. Lett.* **2013**, *15*, 3914–3917.
18 G. Giambastiani, G. Poli, *J. Org. Chem.* **1998**, *63*, 9608–9609.
19 T. Takuwa, T. Minowa, H. Fujisawa, T. Mukayama, *Chem. Pharm. Bull.* **2005**, *53*,476–480.
20 Y. H. Jang, S. W. Youn, *Org. Lett.* **2014**, *16*, 3720–3723.
21 J. Pietruszka, A. C. M. Rieche, T. Wilhelm, A. Witt, *Adv. Synth. Catal.* **2003**, *345*, 1273–1286.
22 M. Schmittel, A. A. Mahajan, G. Bucher, J. W. Bats, *J. Org. Chem.* **2007**, *72*, 2166–2173.
23 J. Li, M. J. Lear, Y. Hayashi, *Angew. Chem. Int. Ed.* **2016**, *55*, 9060–9064.
24 D. Zhang, Y. Huang, E. Zhang, R. Yi, C. Chen, L. Yu, Q. Xu, *Adv. Synth. Catal.* **2018**, *360*, 784–790.
25 D. Dev, N. B. Palakurthy, N. Kumar, B. Mandal, *Tetrahedron Lett.* **2013**, *54*, 4397–4400.
26 Y. Iwata, Y. Tanaka, S. Kubosaki, T. Morita, Y. Yoshimi, *Chem. Commun.* **2018**, *54*, 1257–1260.
27 R. Shang, D. Ji, L. Chu, Y. Fu, L. Liu, *Angew. Chem. Int. Ed.* **2011**, *50*, 4470–4474.
28 Y. Huang, Y. Yu, Z. Zhu, C. Zhu, J. Cen, X. Li, W. Wu, H. Jiang, *J. Org. Chem.* **2017**, *82*, 7621–7627.
29 J. Li, Y. Liu, W. Tang, D. Xue, C. Li, J. Xiao, C. Wang, *Chem. Eur. J.* **2017**, *23*, 14445–14449
30 C. B. Kelly, K. M. Lambert, M. A. Mercadante, J. M. Ovian, W.F. Bailey, N. E. Leadbeater, *Angew. Chem. Int. Ed.* **2015**, *54*, 4241–4245.
31 A. Cardarelly, M. Fagnoni, M. Mella, A. Albini, *J. Org. Chem.* **2001**, *66*, 7320–7327.
32 E. Dolušić, P. Larrieu, L. Moineaux, V. Stroobant, L. Pilotte, D. Colau, L. Pochet, B. V. Eynde, B. Masereel, J. Wouters, R. Frédérick, *J. Med. Chem.* **2011**, *54*, 5320–5334.
33 S. Mannathan, C.-H. Cheng, *Chem. Eur. J.* **2012**, *18*, 11771–11777.
34 J. Gordon, S. Hildebrandt, K. R. Dewese, S. Klare, A. Gansäuer, T. V. RajanBabu, W. A. Nugent, *Organometallics* **2018**, *37*, 4801–4809.
35 R. J. Enemærke, J. Larsen, T. Skrydstrup, K. Daasbjerg, *J. Am. Chem. Soc.* **2004**, *126*, 7853–7864.
36 a) A. Gansäuer, C. Kube, K. Daasbjerg, R. Sure, S. Grimme, G. D. Fianu, D. V. Sadasivam, R. A. Flowers II, *J. Am. Chem. Soc.* **2014**, *136*, 1663–1671. b) A. Gansäuer, M. Behlendorf, D. von Laufenberg, A. Fleckhaus, C. Kube, D. V. Sadasivam, R. A. Flowers II, *Angew. Chem. Int. Ed.* **2012**, *51*, 4739–4742.
37 The internal standard did not interfere with the catalysis.
38 a) C. D.-T. Nielsen, J. Burés, *Chem. Sci.* **2019**, *10*, 348–353. b) J. Burés, *Angew. Chem. Int. Ed.* **2016**, *55*, 2028–2031; *Angew. Chem.* **2016**, *128*, 2068–2071.
39 For excellent reviews on other methods for kinetic profiling and reaction progress kinetics analysis, see: a) D. G. Blackmond, *J. Am. Chem. Soc.* **2015**, *137*, 10852–10866. b) D. G. Blackmond, *Angew. Chem. Int. Ed.* **2005**, *44*, 4302–4320.
40 a) F. Neese, *WIREs* **2012**, *2*, 73–78. b) F. Neese, *WIREs* **2017**, *8*, e1327.
41 a) K. Eichkorn, O. Treutler, H. Öhm, M. Häser, R. Ahlrichs, *Chem. Phys. Lett.* **1995**, *240*, 283–290. b) K. Eichkorn, F. Weigend, O. Treutler, R. Ahlrichs, *Theor. Chem. Acc.* **1997**, *97*, 119–124. c) R. Bauernschmitt, M. Häser, O. Treutler, R. Ahlrichs, *Chem. Phys. Lett.* **1997**, *264*, 573–578. d) P. Deglmann, K. May, F. Furche, R. Ahlrichs, *Chem. Phys. Lett.* **2004**, *384*, 103–107. e) C. K. Skylaris, L. Gagliardi, N. C. Handy, G. Ioannou, S. Spencer, A. Willets, *J. Mol. Struct. (THEOCHEM)* **2000**, *501*, 229–239.
42 F. Neese, F. Wennmohs, A. Hansen, U. Becker, *Chem. Phys.* **2009**, *356*, 98–109.
43 a) S. Grimme, A. Hansen, J. G. Brandenburg, C. Bannwarth, *Chem. Rev.* **2016**, *116*, 5105–5154. b) S. Grimme, J. Antony, S. Ehrlich, H. Krieg, *J. Chem. Phys.* **2010**, *132*, 154104.
44 a) S. Grimme, S. Ehrlich, L. Goerigk, *J. Comput. Chem.* **2011**, *32*, 1456–1465. b) A. D. Becke, E. R. Johnson, *J. Chem. Phys.* **2005**, *123*, 154101. c) E. R. Johnson, A. D. Becke, *J. Chem. Phys.* **2005**, *123*, 24101.
45 J. Tao, J. P. Perdew, V. N. Staroverov, G. E. Scuseria, *Phys. Rev. Lett.* **2003**, *91*, 146401.
46 F. Weigend, R. Ahlrichs, *Phys. Chem. Chem. Phys.* **2005**, *7*, 3297–3305.
47 F. Weigend, *Phys. Chem. Chem. Phys.* **2006**, *8*, 1057–1065.
48 M. Cossi, N. Rega, G. Scalmani, V. Barone, *J. Comput. Chem.* **2003**, *24*, 669–681.

49 Y. Zhao, D. G. Truhlar, *J. Phys. Chem. A* **2005**, *109*, 5656–5667.
50 a) F. Weigend, F. Furche, R. Ahlrichs, *J. Chem. Phys.* **2003**, *119*, 12753. b) A. Hellweg, C. Hättig, S. Höfener, W. Klopper, *Theor. Chem. Acc.* **2007**, *117*, 587–597.
51 See: J. Streuff, D. Himmel, S. L. Younas, *Dalton Trans.* **2018**, *47*, 5072–5082.

NMR Spectra of New Compounds

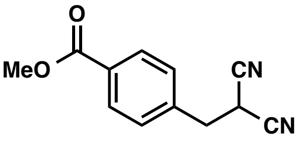

1d

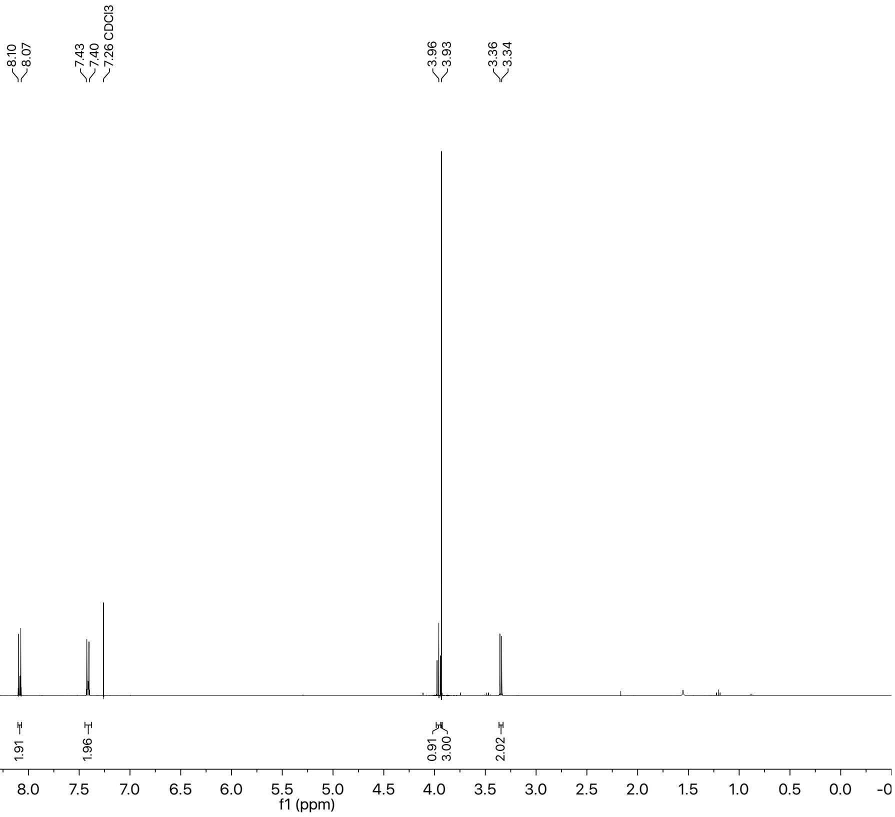

^{1}H NMR (400 MHz, $CDCl_3$) of compound **1d.**

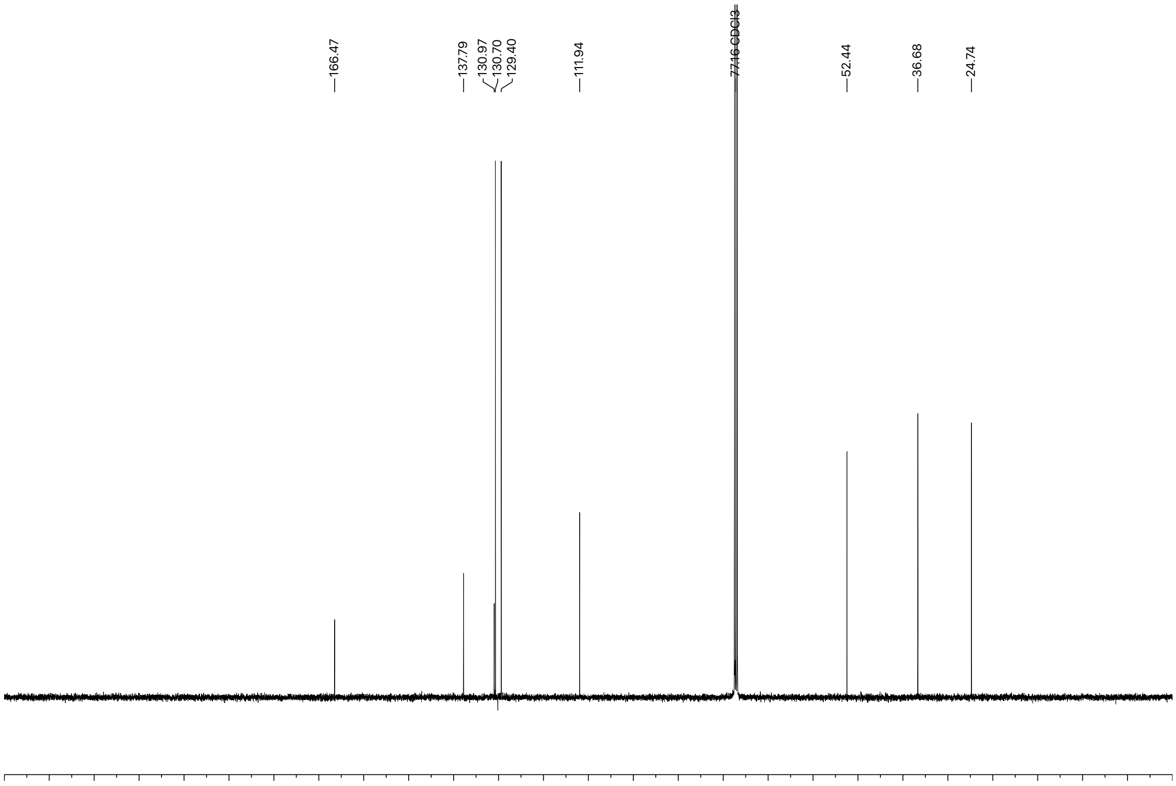

^{13}C NMR (100 MHz, $CDCl_3$) of compound **1d.**

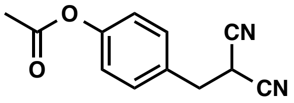

1e

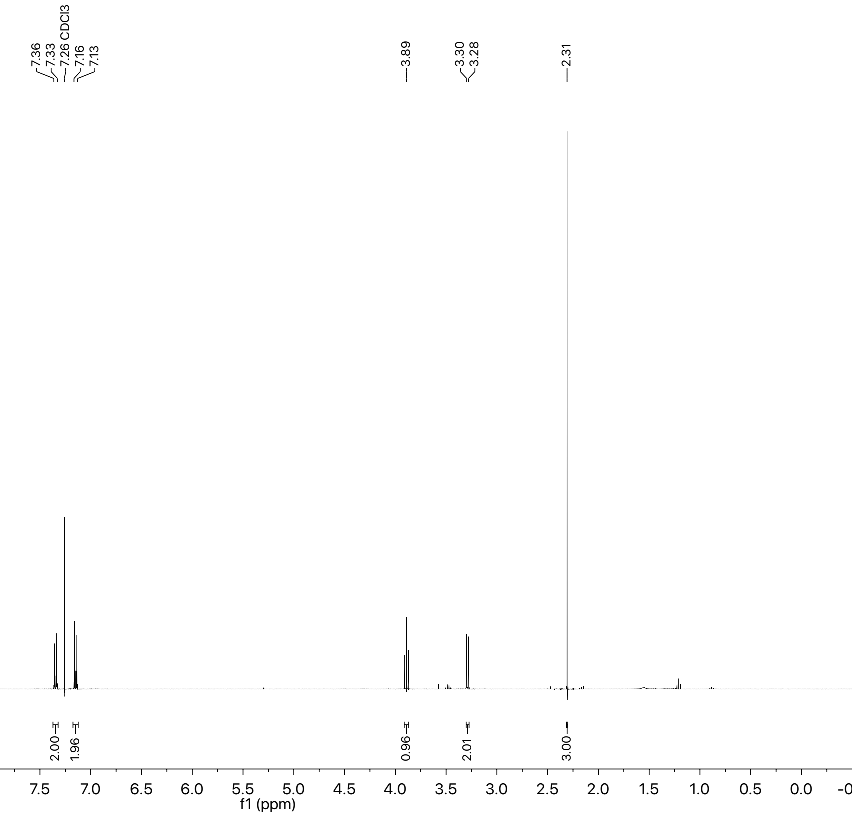

^{1}H NMR (500 MHz, $CDCl_3$) of compound **1e.**

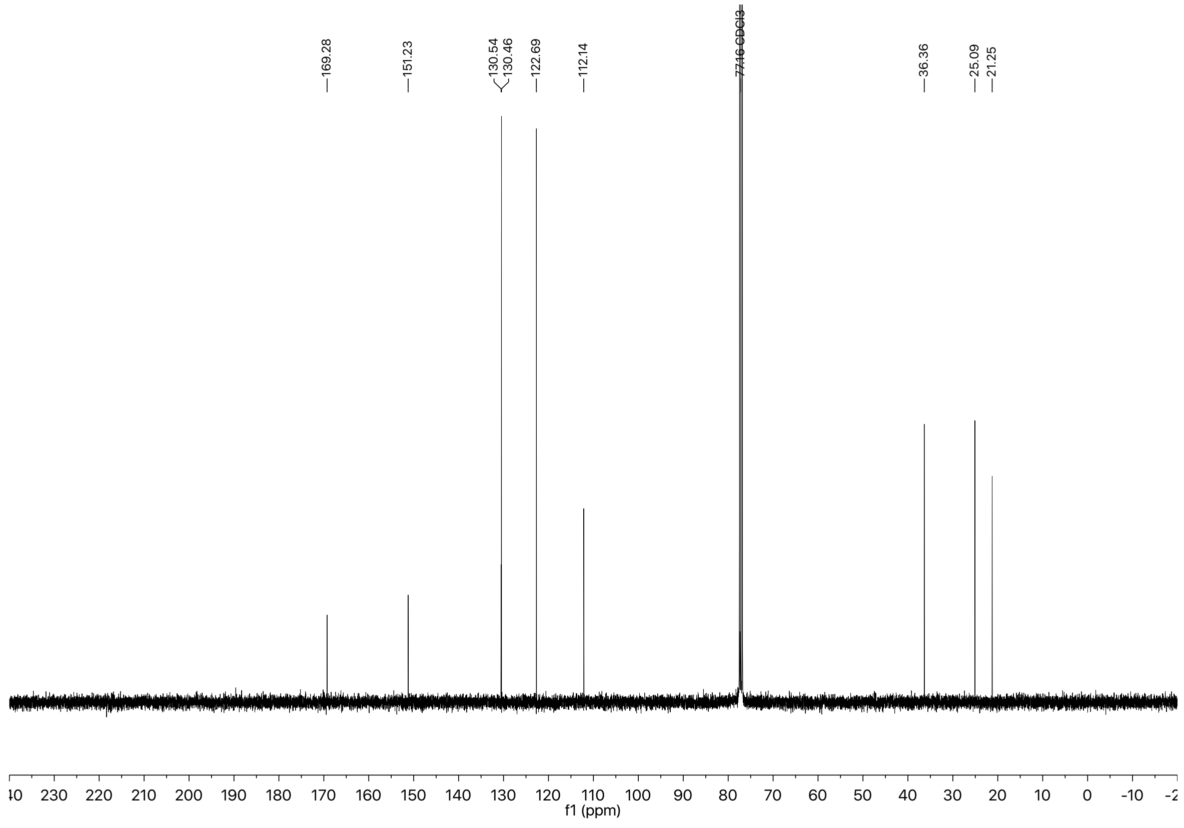

^{13}C NMR (125 MHz, $CDCl_3$) of compound **1e.**

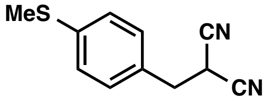

1g

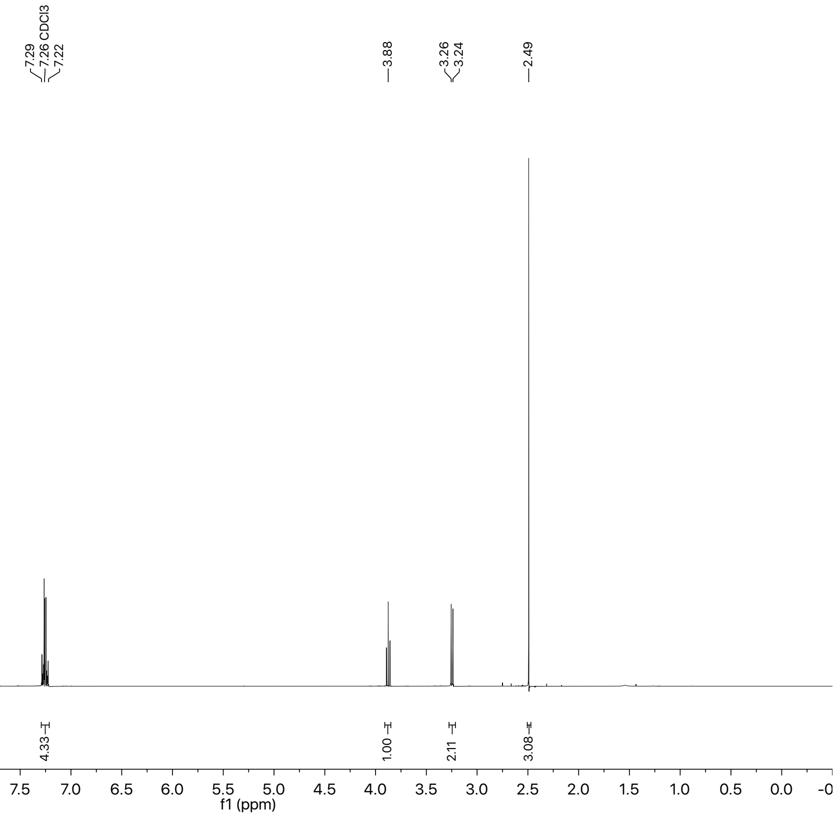

^{1}H NMR (400 MHz, $CDCl_3$) of compound **1g.**

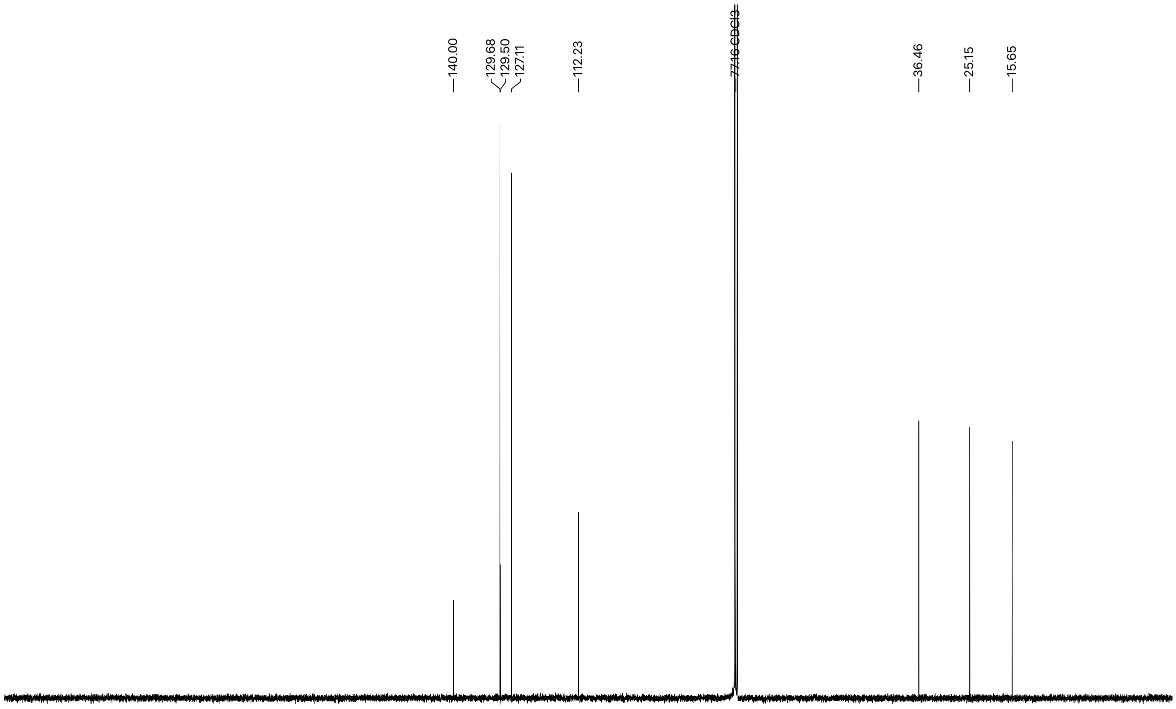

^{13}C NMR (100 MHz, CDCl3) of compound **1g**

.

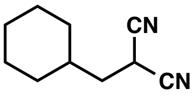

1o

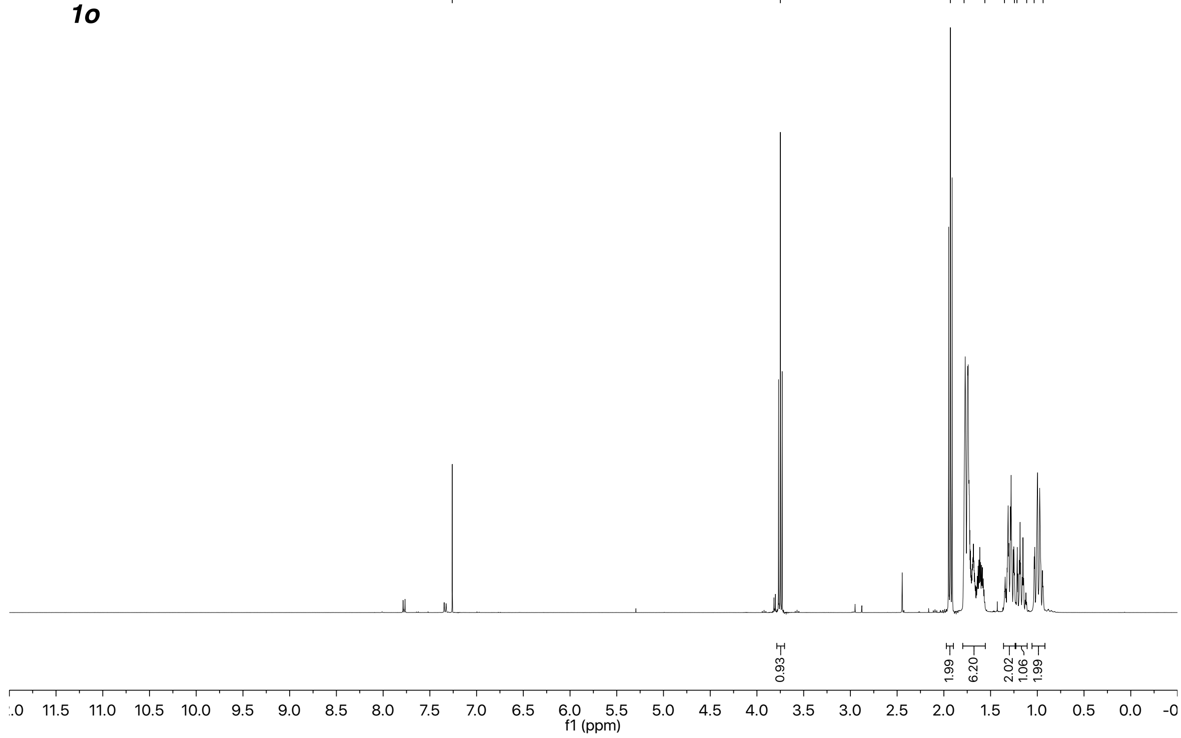

^{1}H NMR (400 MHz, $CDCl_3$) of compound **1o.**

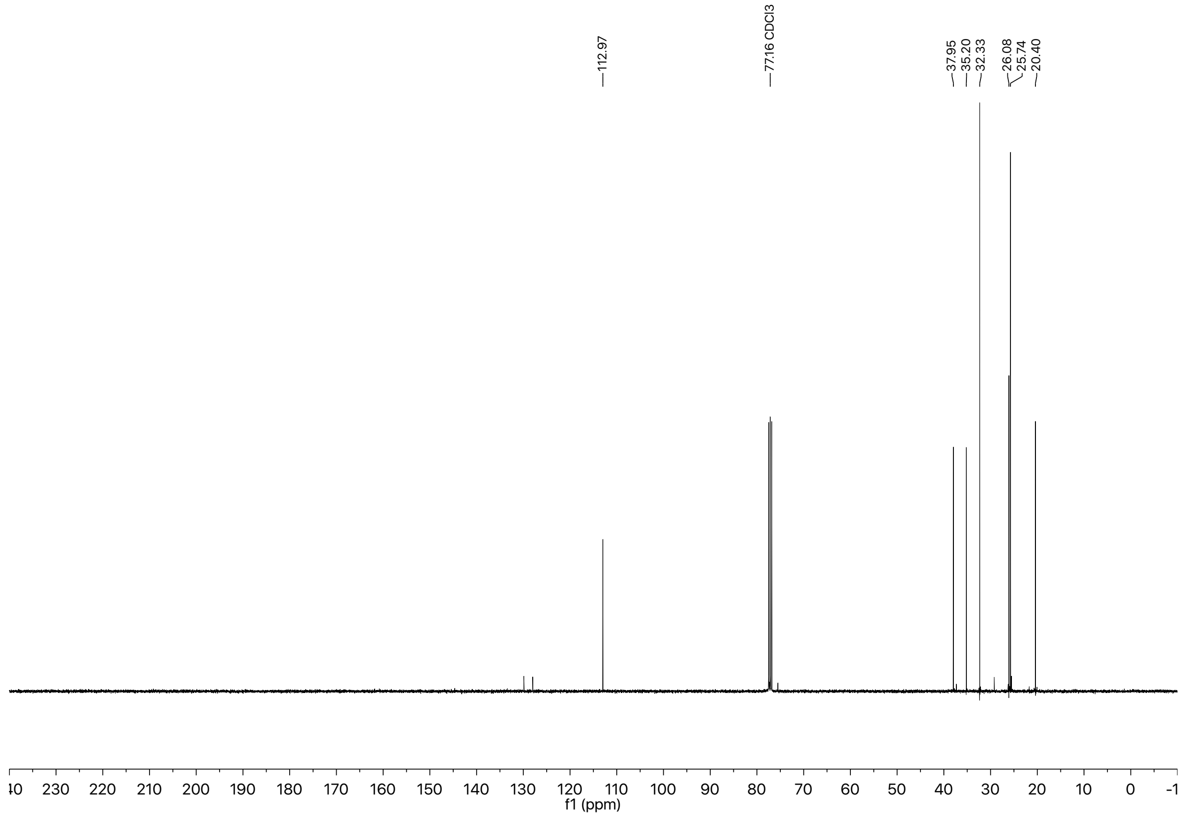

^{13}C NMR (100 MHz, $CDCl_3$) of compound **1o.**

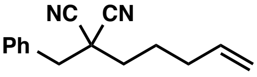

4

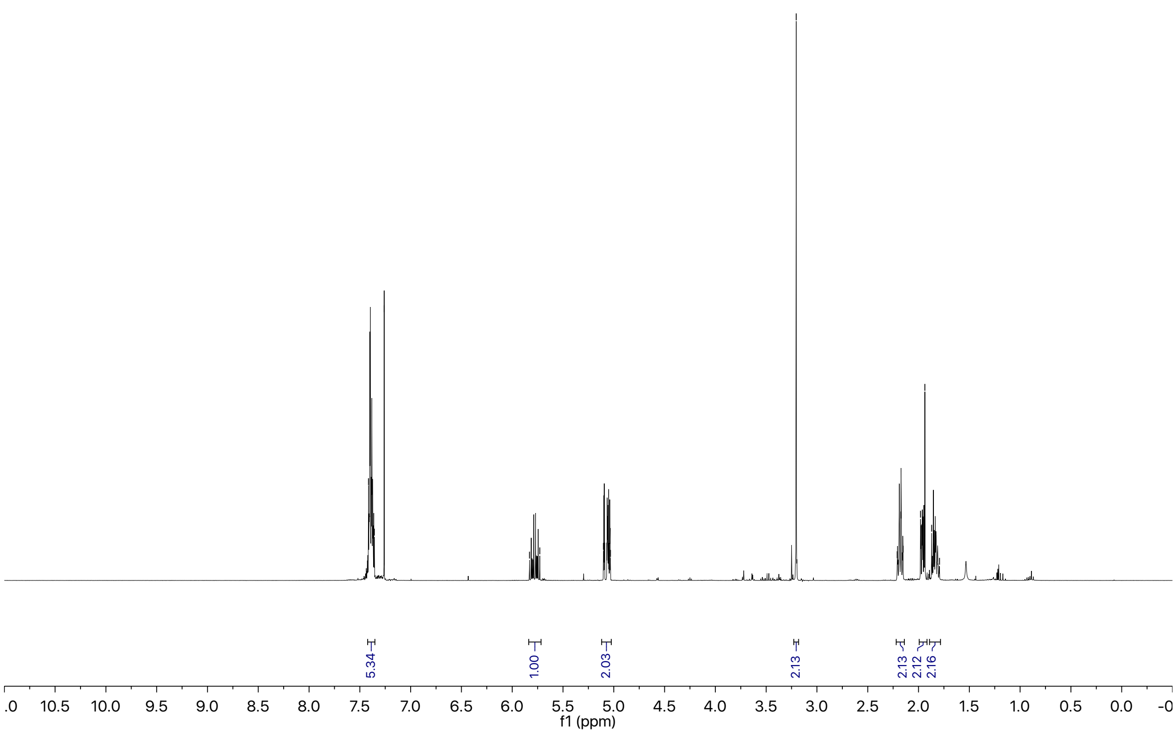

^{1}H NMR (400 MHz, $CDCl_3$) of compound **4.**

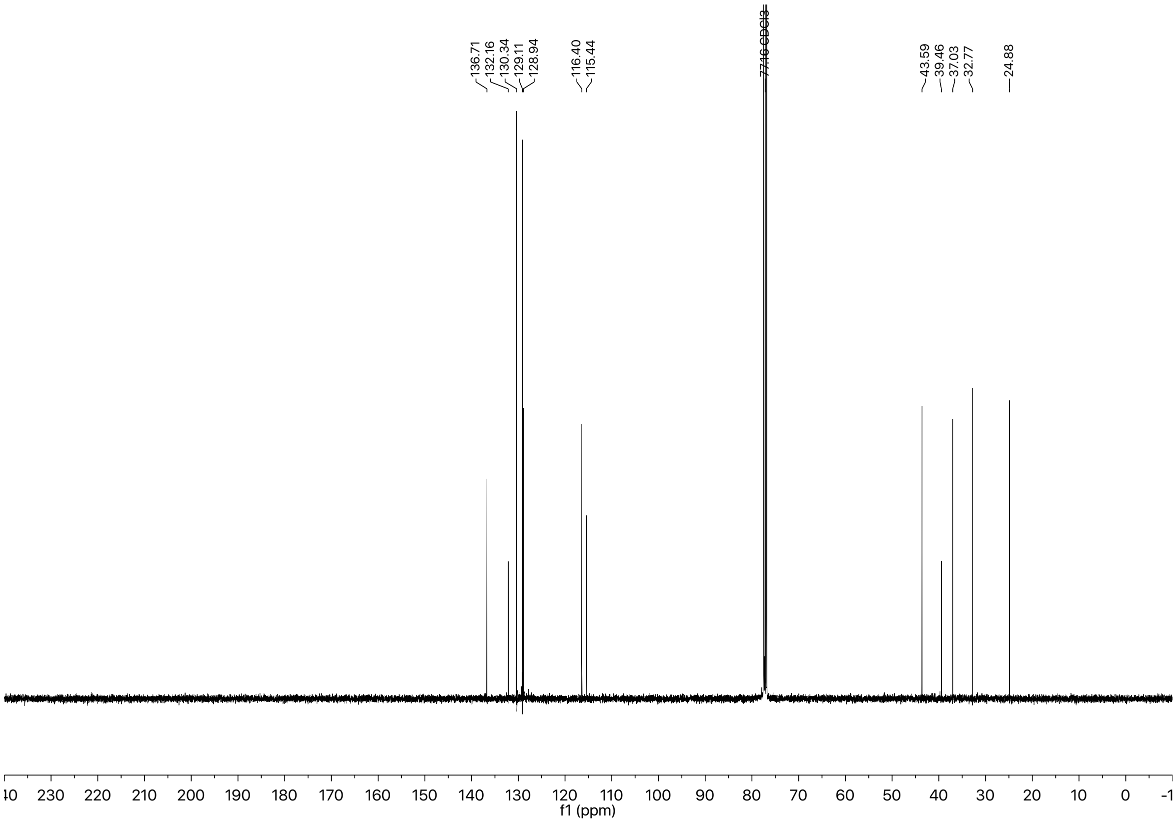

^{13}C NMR (100 MHz, $CDCl_3$) of compound **4.**

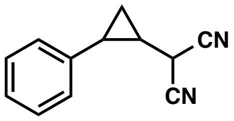

7

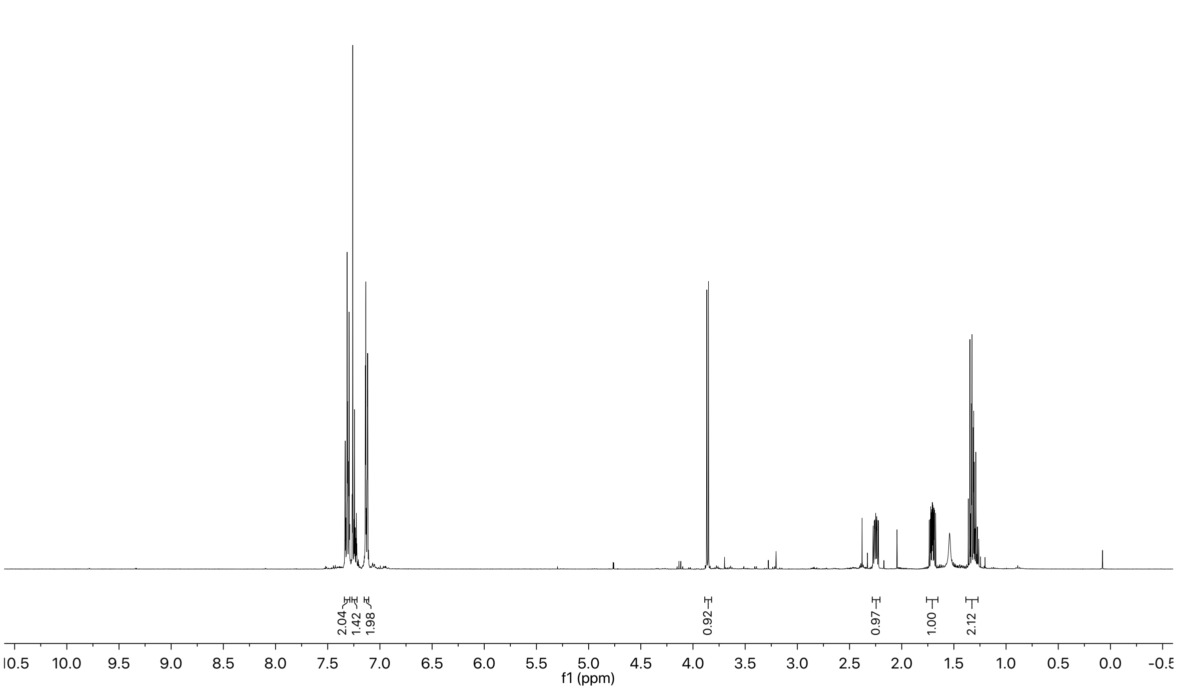

^{1}H NMR (400 MHz, $CDCl_3$) of compound **7.**

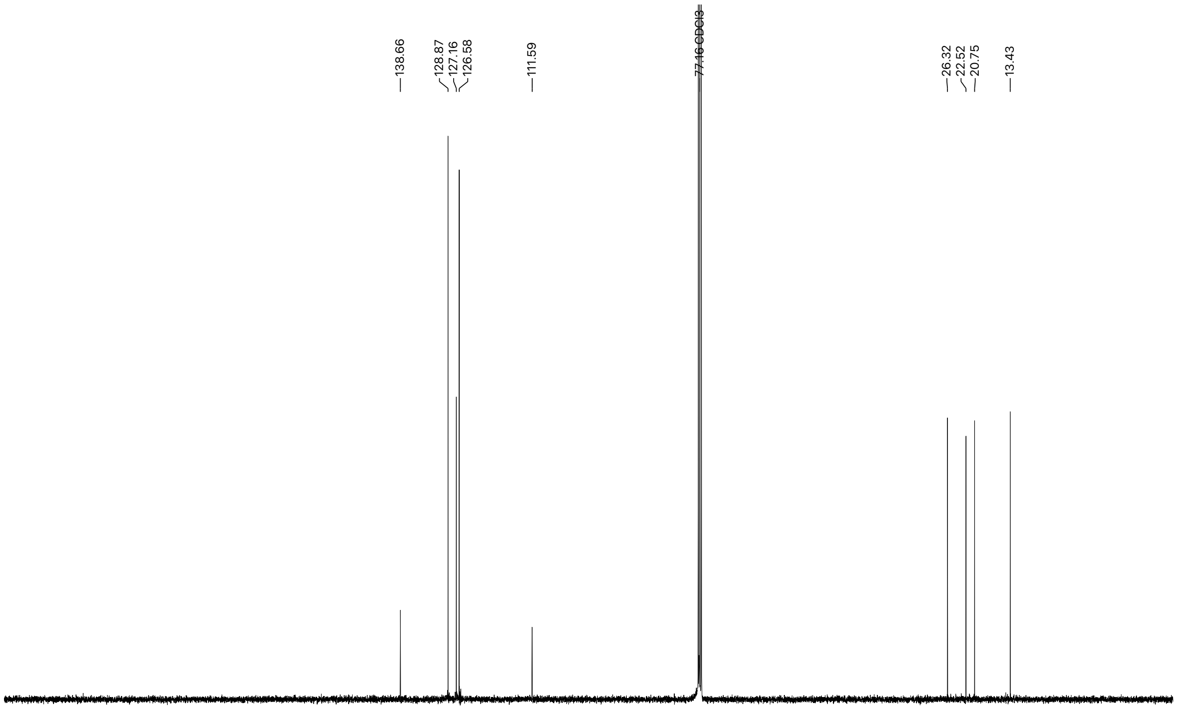

^{13}C NMR (100 MHz, $CDCl_3$) of compound **7.**

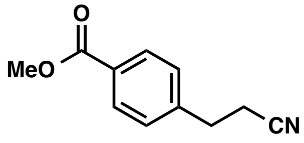

2d

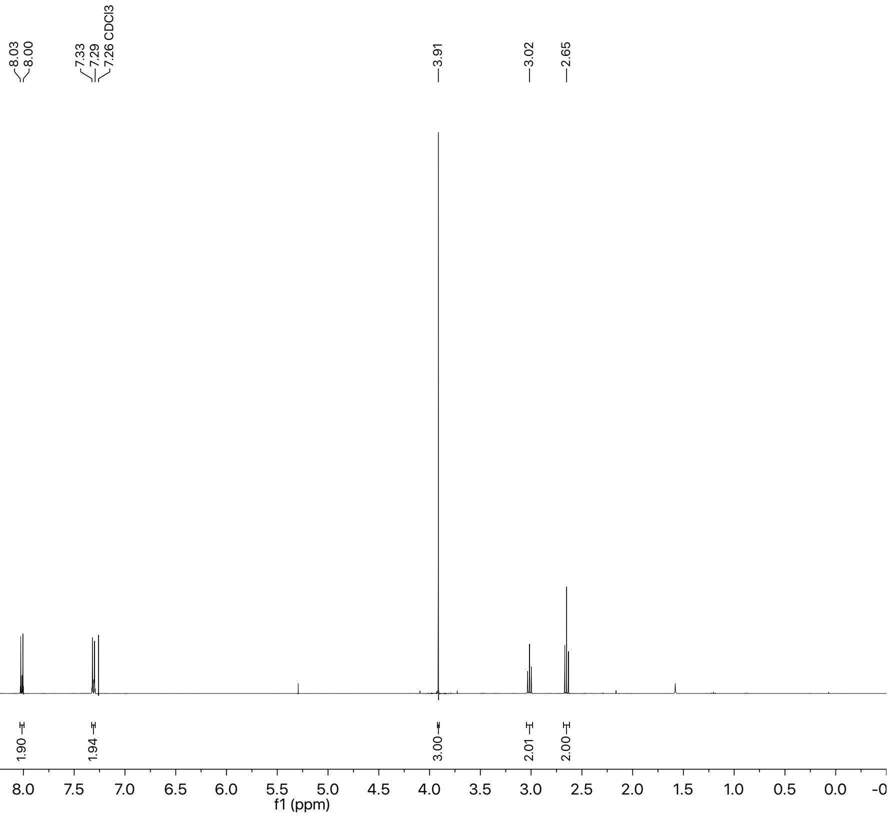

^{1}H NMR (400 MHz, $CDCl_3$) of compound **2d.**

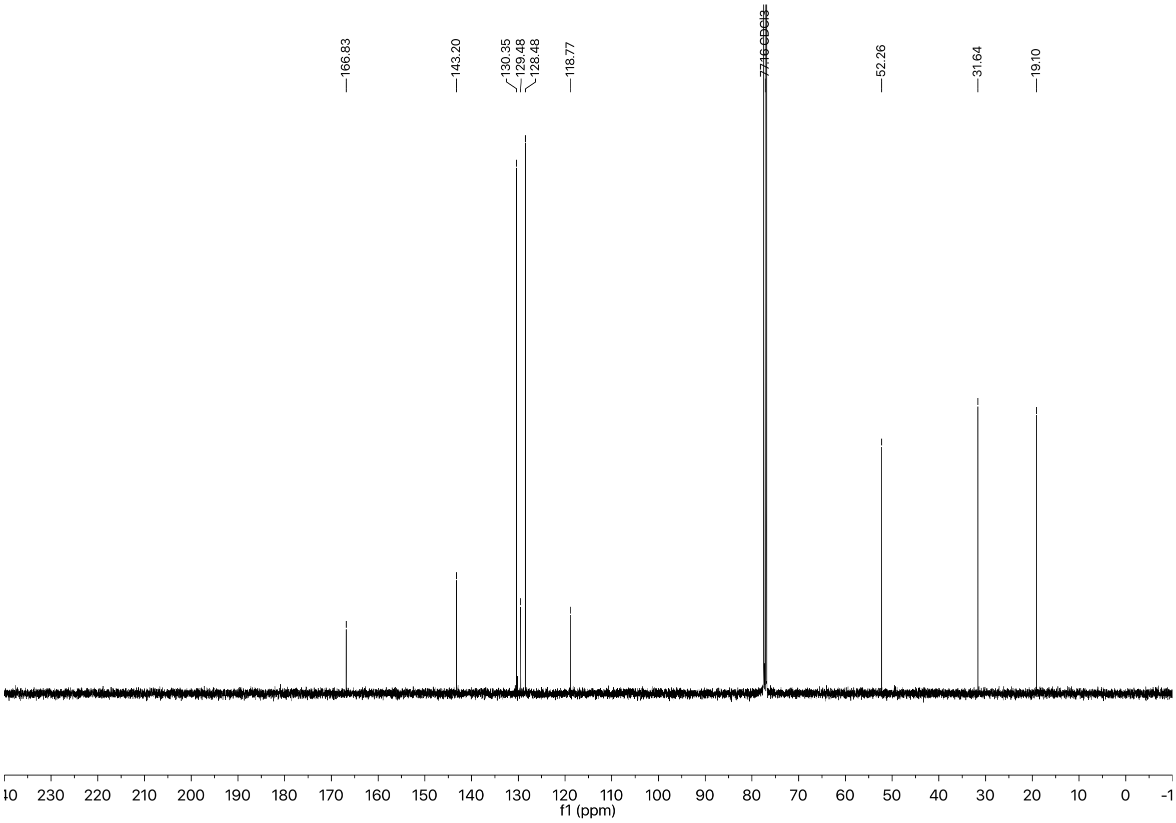

^{13}C NMR (100 MHz, $CDCl_3$) of compound **2d.**

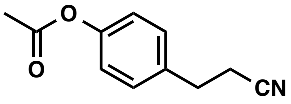

2e

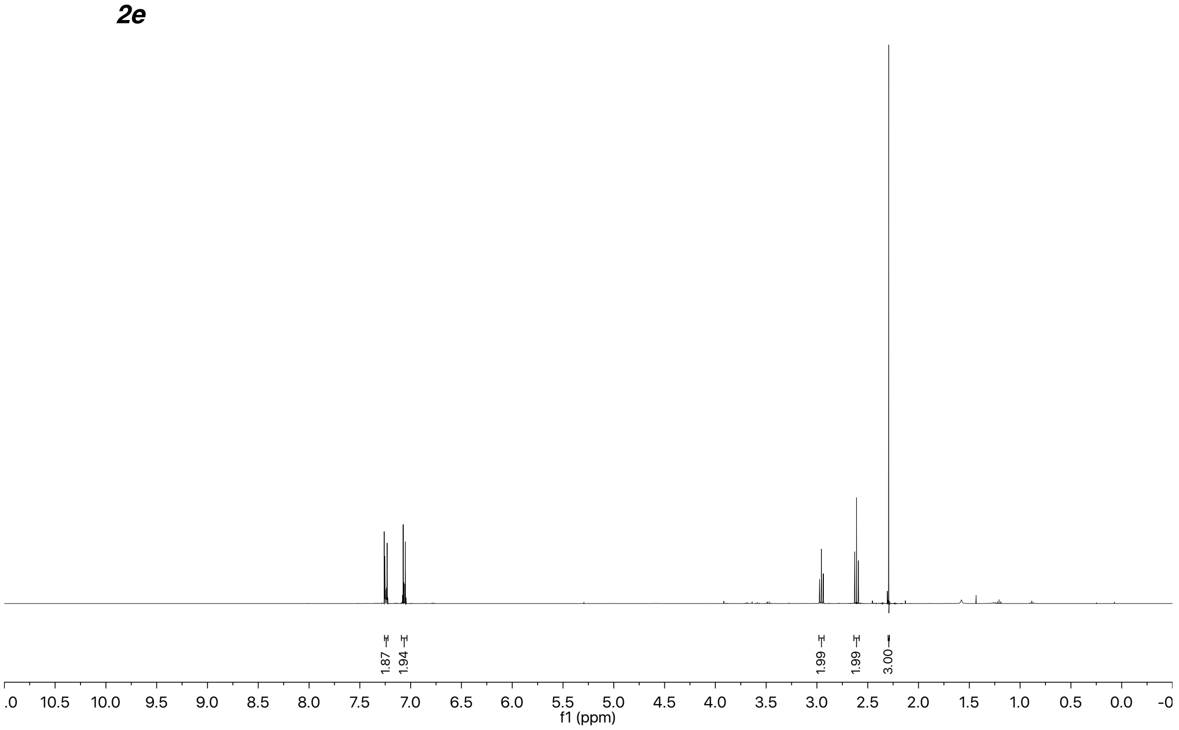

^{1}H NMR (400 MHz, $CDCl_3$) of compound **2e.**

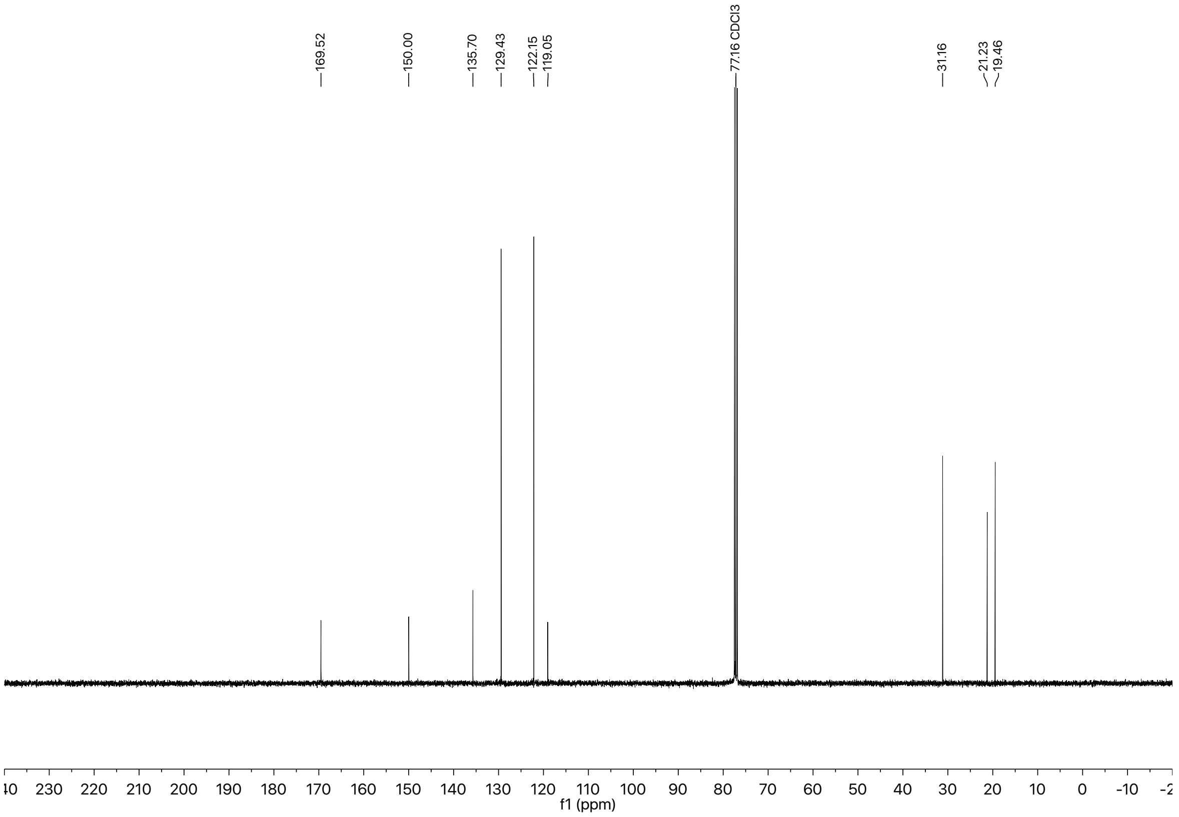

^{13}C NMR (100 MHz, $CDCl_3$) of compound **2e.**

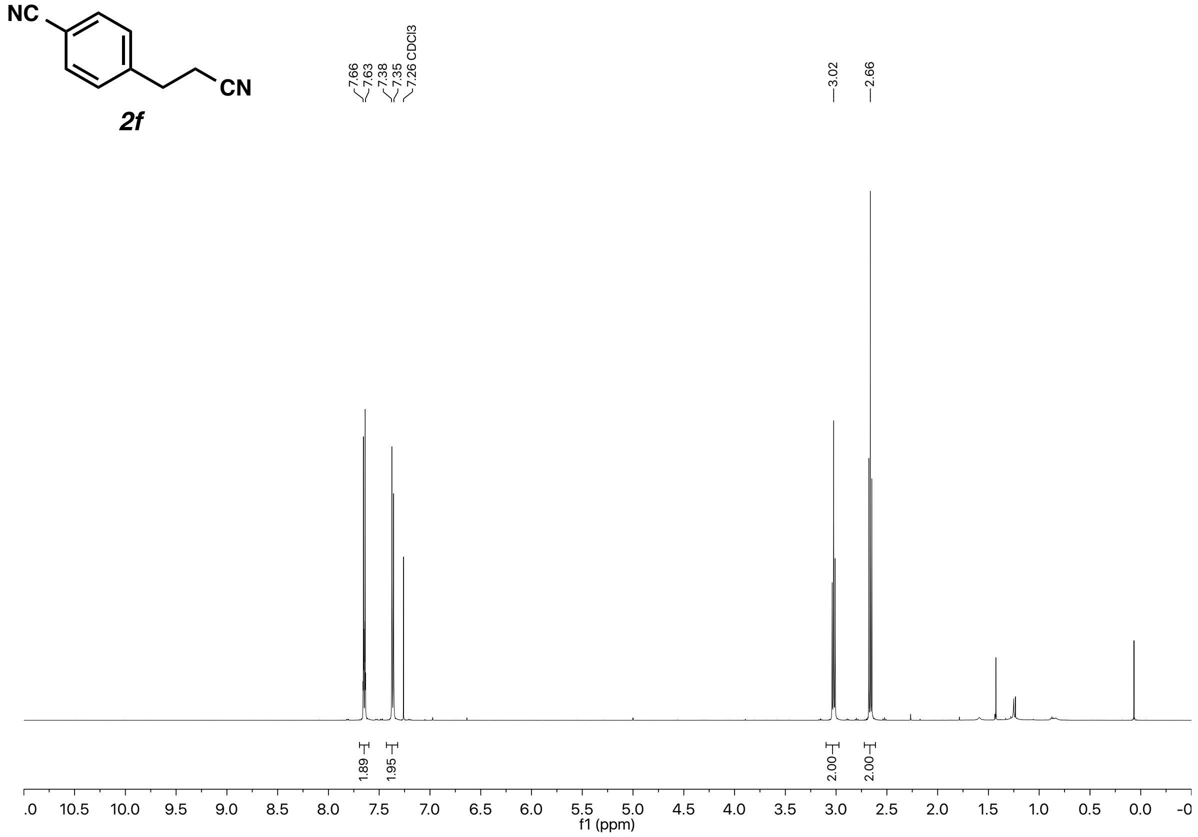

^{1}H NMR (500 MHz, $CDCl_3$) of compound **2f.**

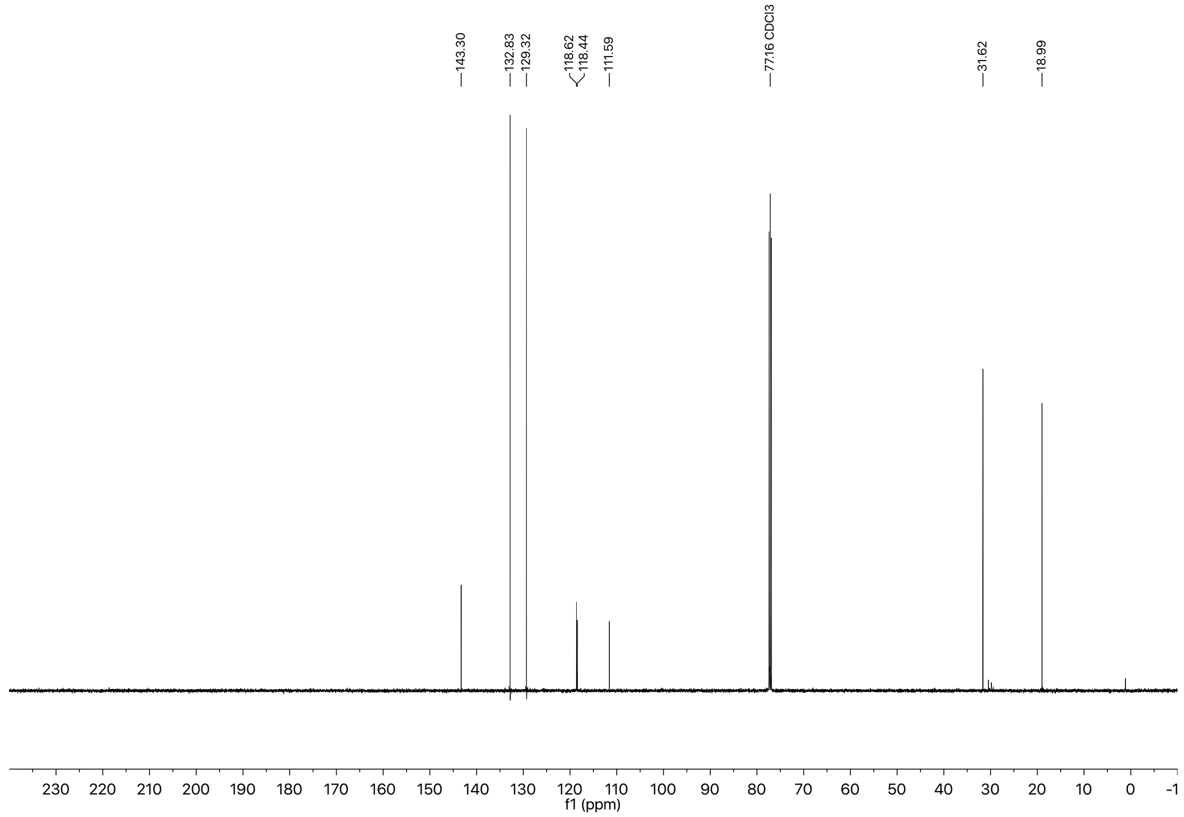

^{13}C NMR (125 MHz, $CDCl_3$) of compound **2f.**

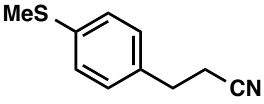

2g

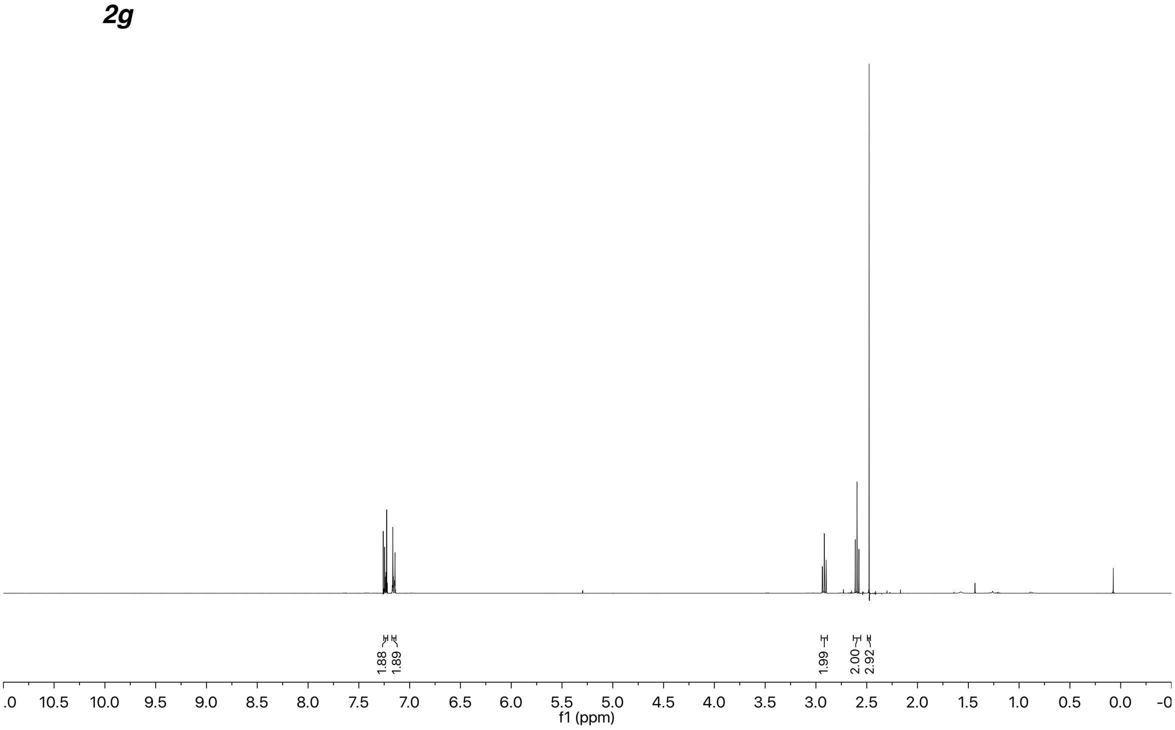

^{1}H NMR (400 MHz, $CDCl_3$) of compound **2g.**

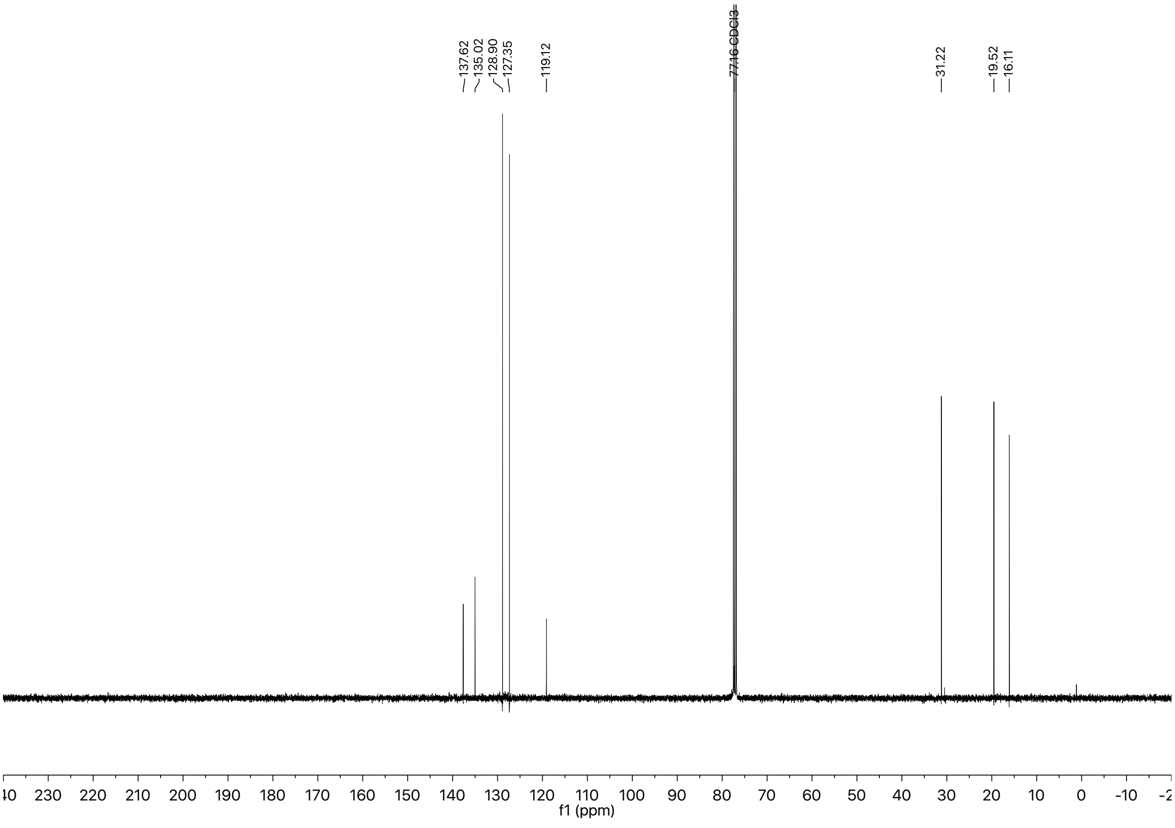

^{13}C NMR (100 MHz, $CDCl_3$) of compound **2g.**

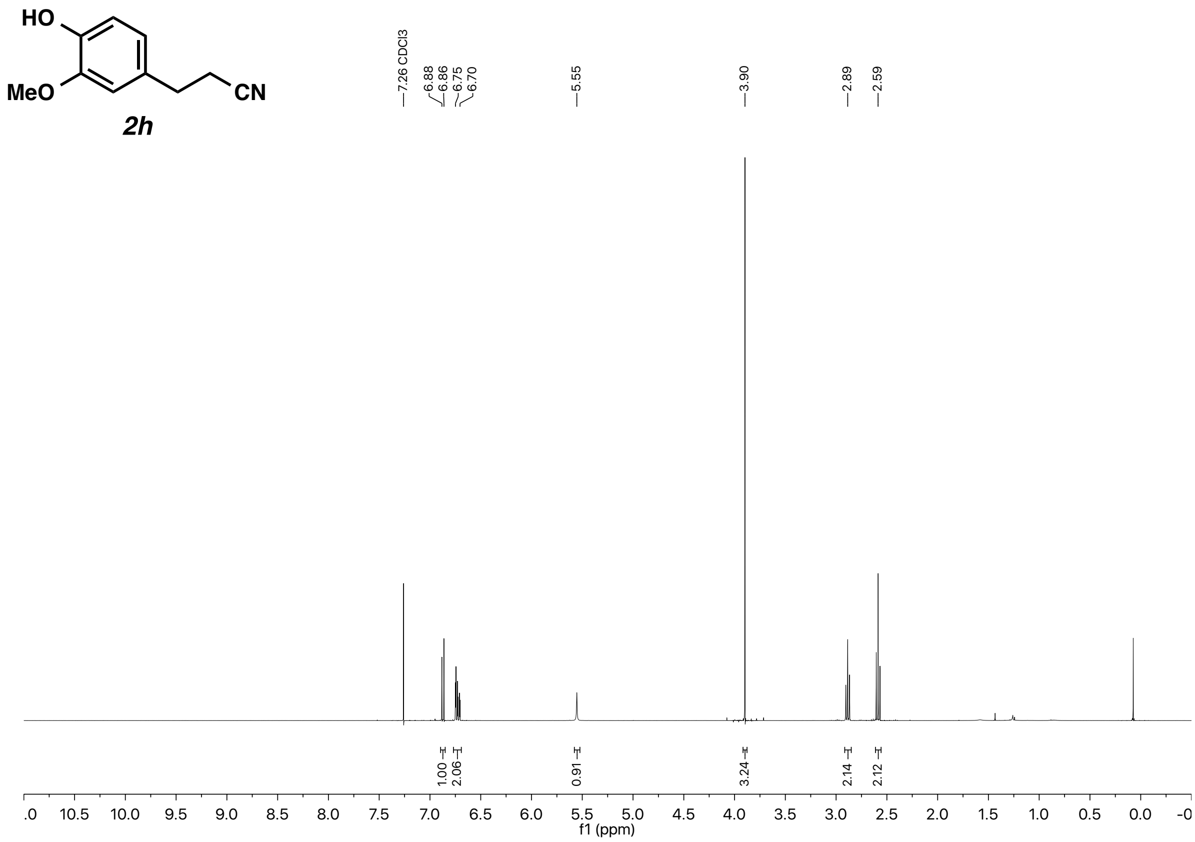

^{1}H NMR (400 MHz, $CDCl_3$) of compound **2h.**

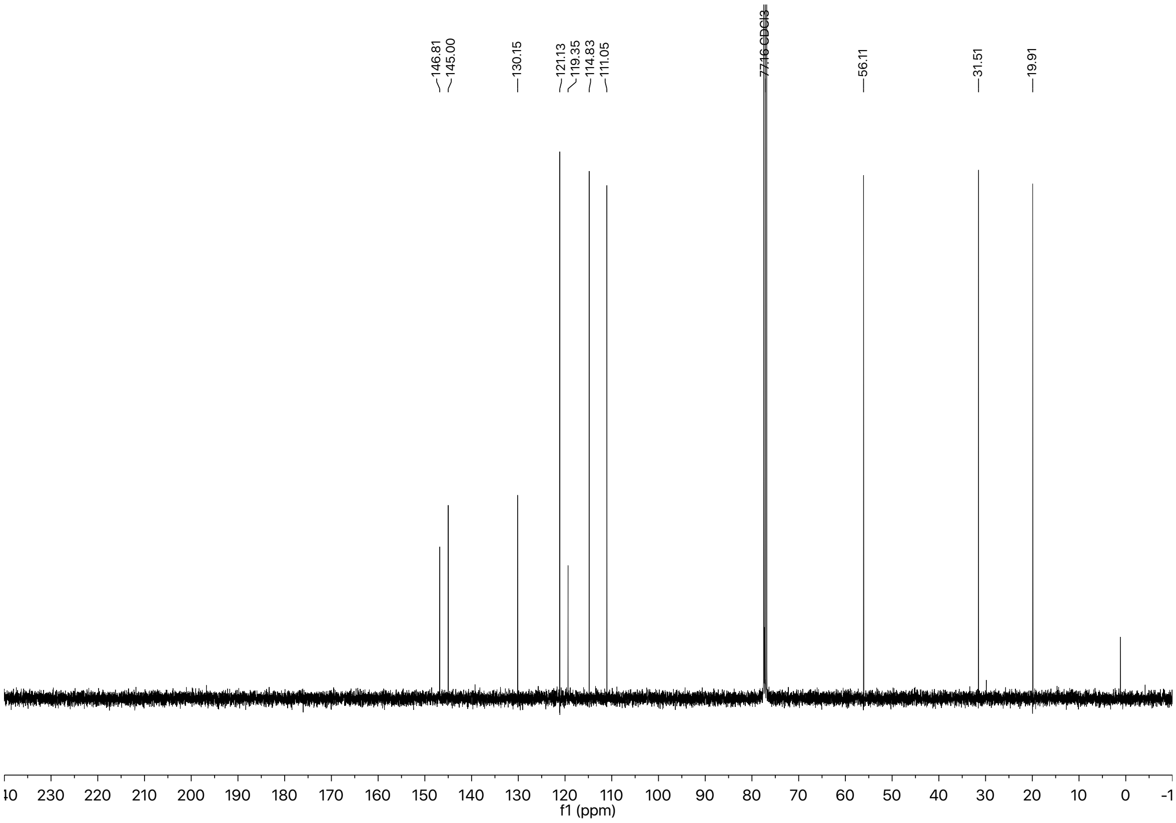

^{13}C NMR (100 MHz, $CDCl_3$) of compound **2h.**

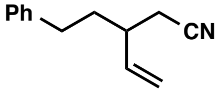

2r

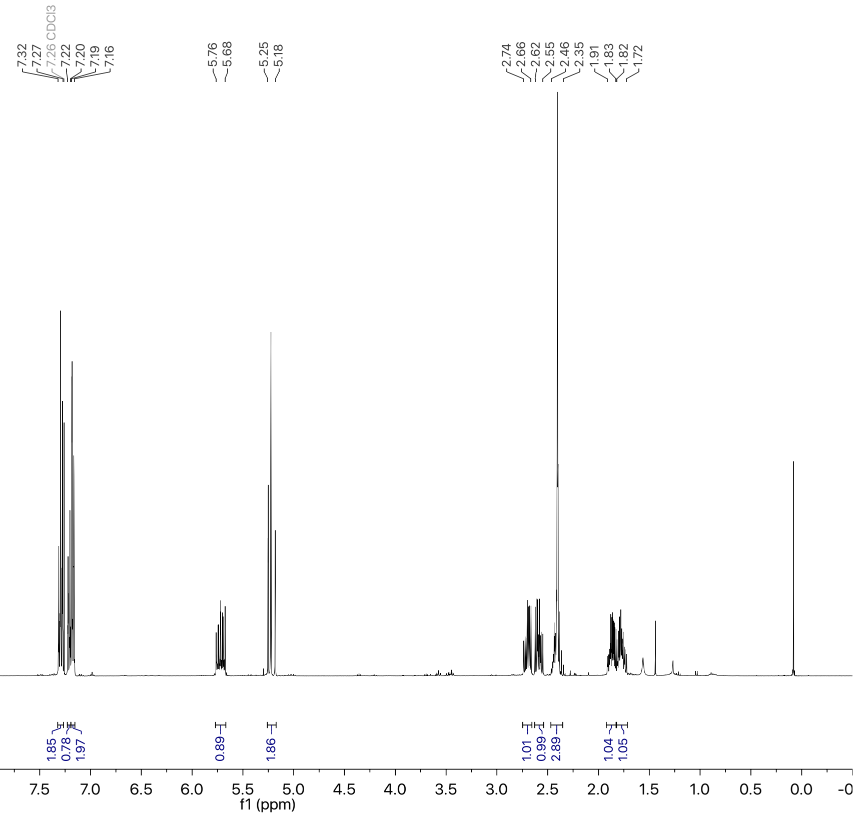

^{1}H NMR (400 MHz, $CDCl_3$) of compound **2r.**

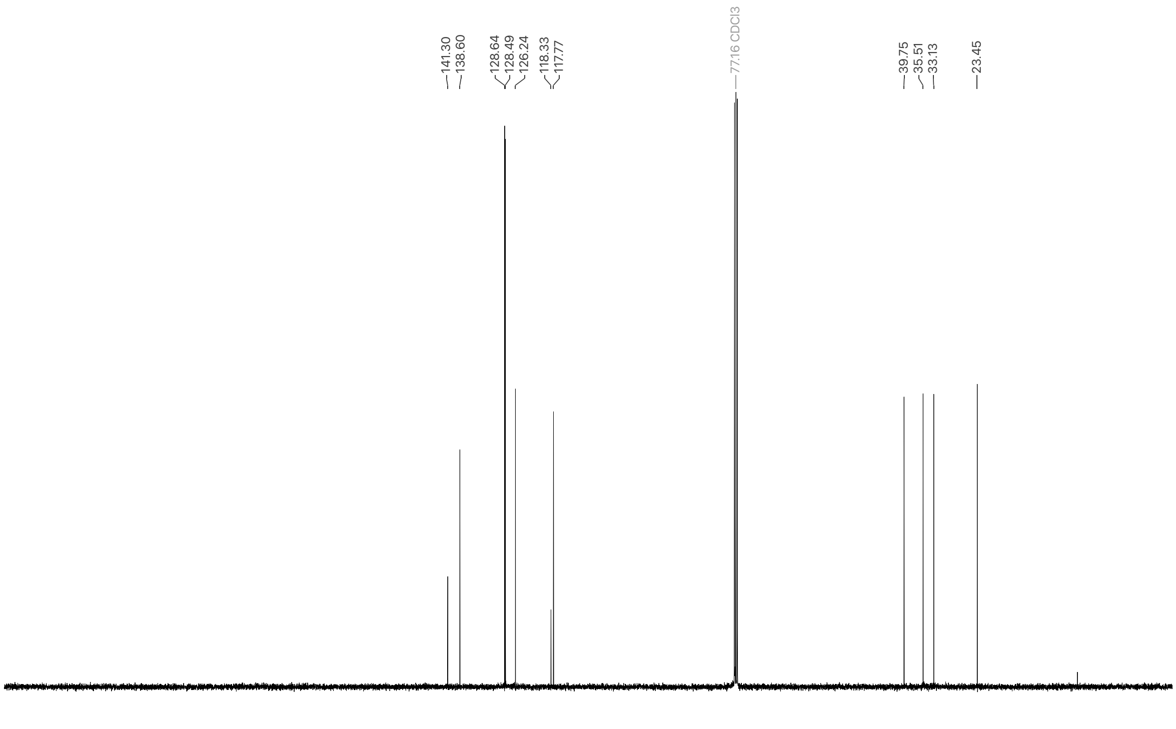

^{13}C NMR (100 MHz, $CDCl_3$) of compound **2r.**

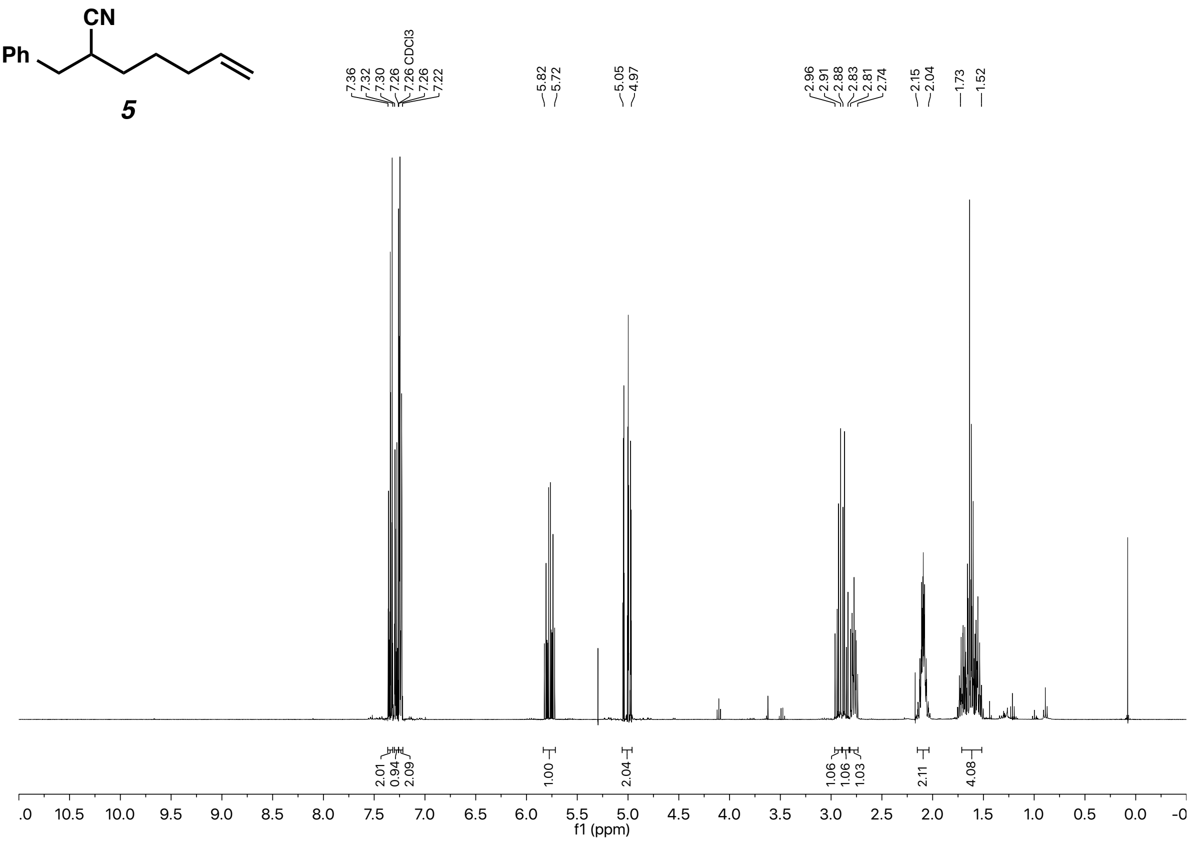

^{1}H NMR (400 MHz, CDCl$_3$) of compound **5.**

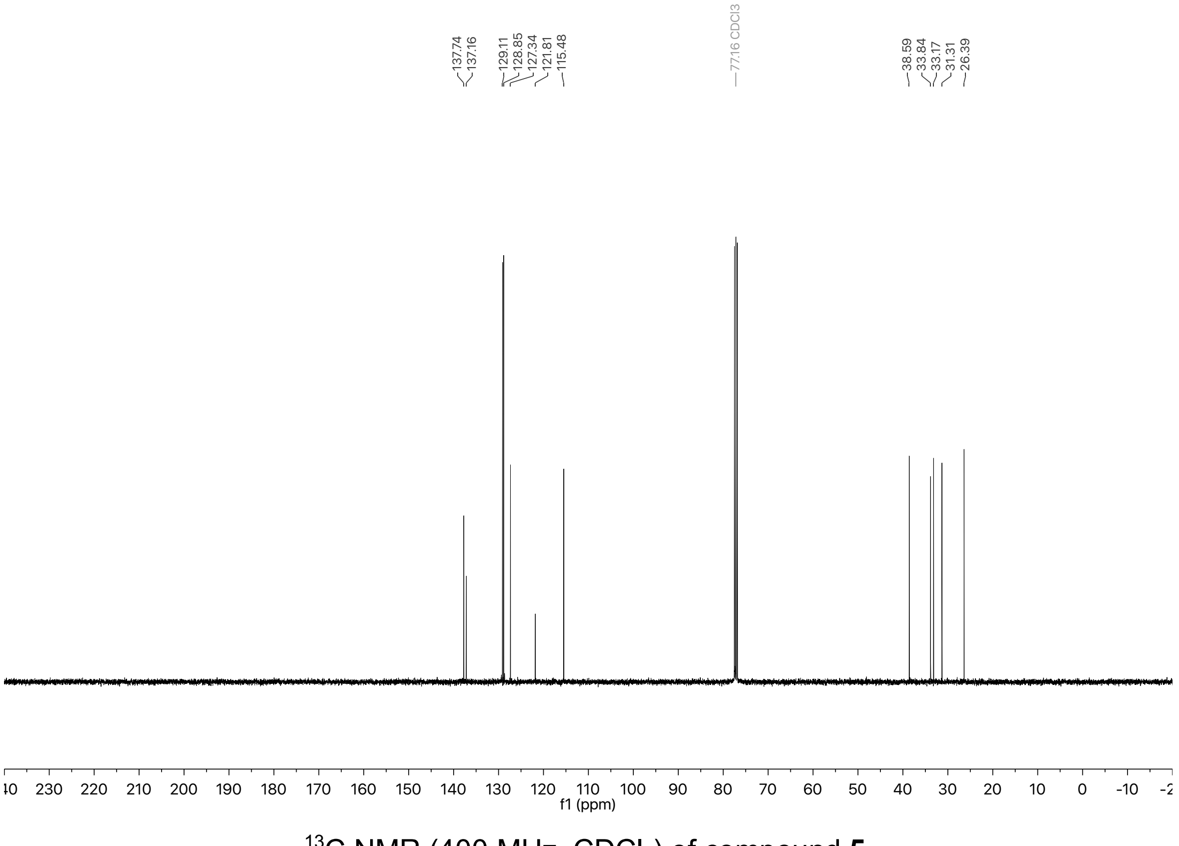

^{13}C NMR (400 MHz, CDCl$_3$) of compound **5.**

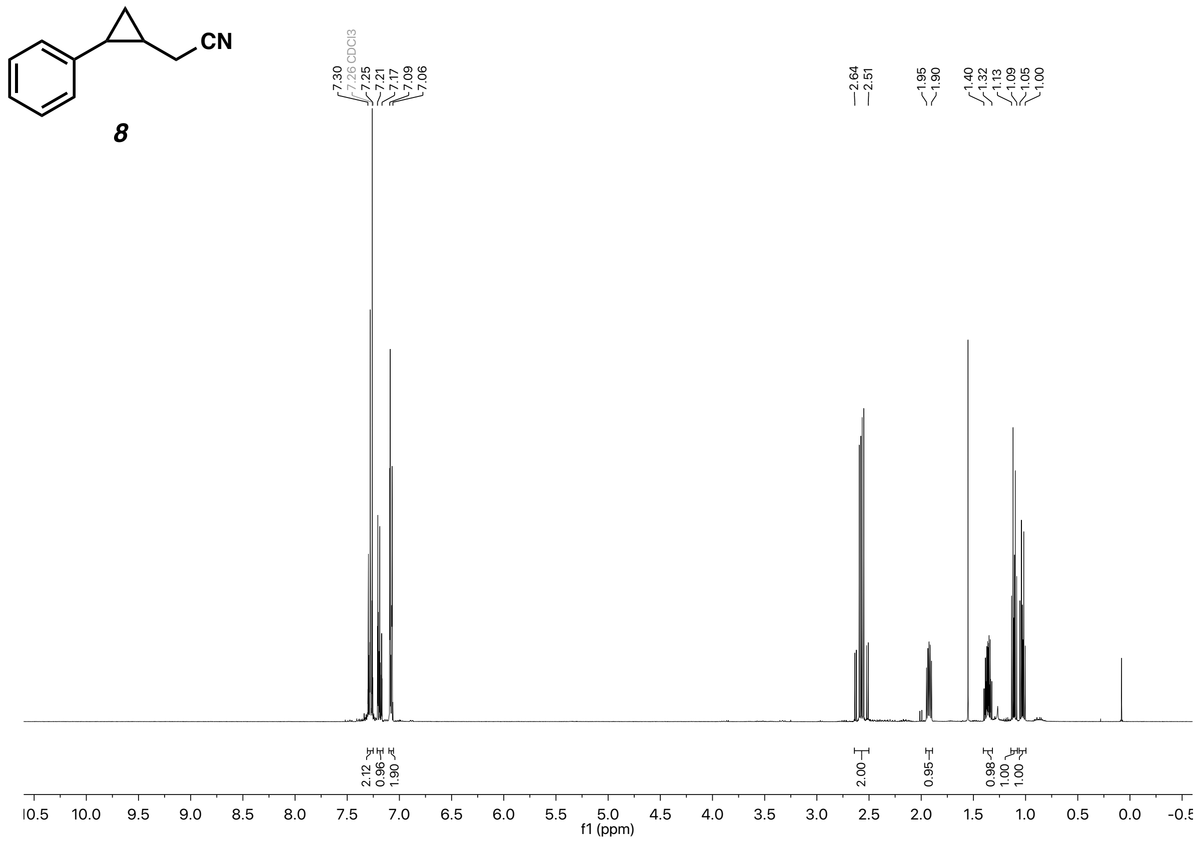

^{1}H NMR (400 MHz, $CDCl_3$) of compound **8.**

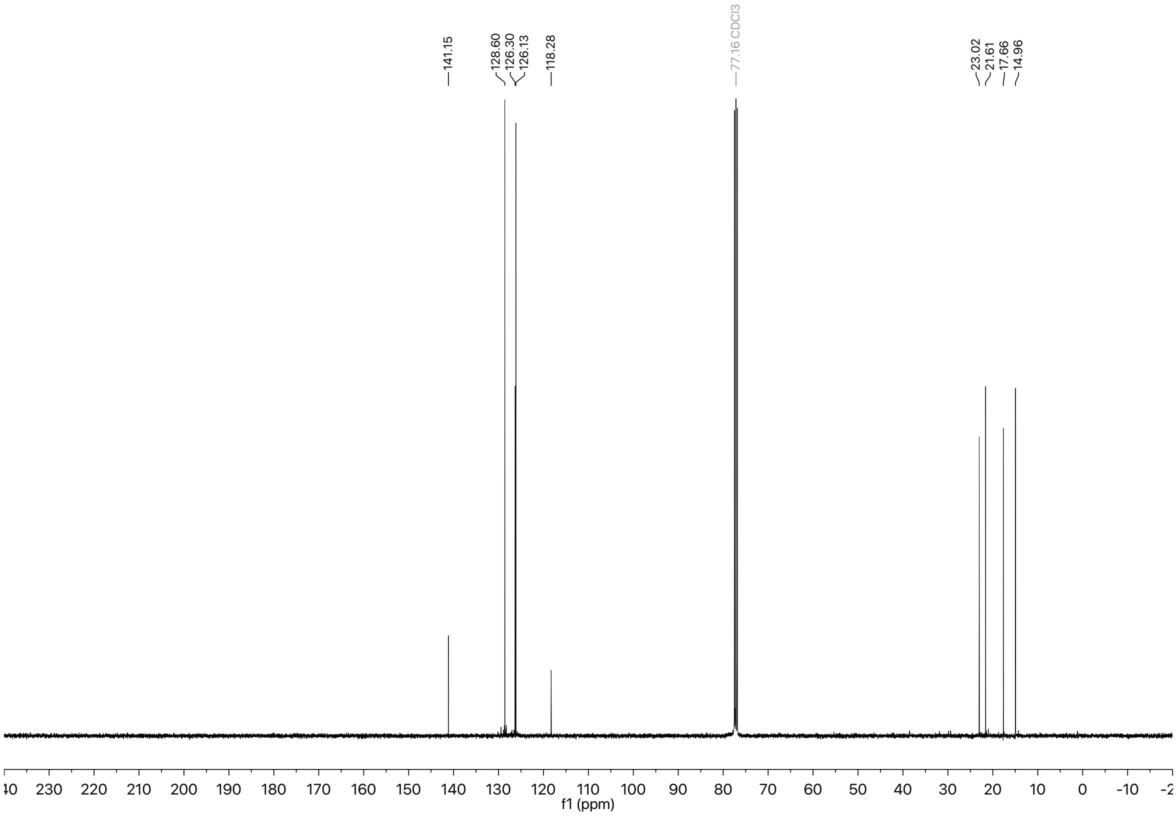

^{13}C NMR (100 MHz, $CDCl_3$) of compound **8.**
